# Identifying DNA mutations in *Sepia*, *White*, *Yellow*, and *Vestigial* genes in *D. melanogaster* - Integrating inquiry-based experiential learning into undergraduate Genetics Laboratory curriculum

**DOI:** 10.1371/journal.pone.0356998

**Published:** 2026-09-01

**Authors:** Krishna K. Rentachintala, Catherine A. K. Bricker, Parker R. Jain, Meera Nanjundan

**Affiliations:** Department of Molecular Biosciences, University of South Florida, Tampa, Florida, United States of America; University of Alabama at Birmingham, UNITED STATES OF AMERICA

## Abstract

Eye, body, and wing morphology in *D. melanogaster* are distinctly tied to genes that can easily be monitored to decipher the association between phenotype and genotype. Toward the goal of uncovering the DNA mutations responsible for these phenotypes, we share the development and implementation of a semester-long upper-level general Genetics laboratory course, in which an inquiry-based research component is integrated into the curriculum. The experimental design can be adaptable and covers essential molecular biology concepts as well as experimental procedures. Specifically, we obtained mutant and wild-type fly populations to uncover mutations that inhibit gene expression, protein production, and/or function of the *sepia*, *white*, *yellow*, and *vestigial* genes. We amplified regions of interest to identify novel and previously annotated DNA mutations that may be responsible for observed mutant phenotypes. Further analysis of these and other genes may advance our understanding of their function and regulation that mediate eye color, body pigment, and wing development. Program evaluation was performed by collecting student perspectives in an end-of-semester course survey, and responses were analyzed to identify that they valued learning hands-on protocols including amplifying and cloning DNA, learning scientific methods involving experimental design and data analysis, making connections to real-world applications, as well as sharing their reflection on career interests. In addition, flexible, sustainable, and creative extracurricular opportunities in research and curriculum development for undergraduate and graduate students are associated with the implementation of our General Genetics Laboratory course. Altogether, our curriculum development extends beyond the classroom and aims to support students in research experiences and future career advancements.

## Introduction

Connecting molecular biological concepts and principles that students learn in lecture to their hands-on work in a laboratory setting is vital to a well-rounded understanding and appreciation of established and emerging techniques in the field. Undergraduate research experiences support student’s career decisions [1]. Specifically, colleges and employers seek those who have engaged in career readiness competencies including skills developed through laboratory and research work, as well as participation in hands-on research which increase understanding of the field and contribute to interest in advanced degrees [2,3]. Therefore, providing students with opportunities for practical application of molecular biology topics may provide a foundational step in their research journey. This paper aims to describe a high enrollment undergraduate upper-level Genetics Laboratory course in which we develop and integrate an inquiry-based research project into the curriculum. In addition, undergraduate and graduate student extracurricular research findings that support experiential learning opportunities contributed to the successful curriculum development and implementation of this course.

*D. melanogaster*, used as a model organism incorporated into an undergraduate laboratory, offers students an opportunity to work with live animals. The low cost associated with obtaining and maintaining the flies, along with their rapid life cycle, makes it possible for students to follow multiple generations [4]. Additionally, distinct and easily observable phenotypes suitable to associate with their genotypes make *D. melanogaster* a versatile model for a Genetics laboratory course.

**Fig 1:**
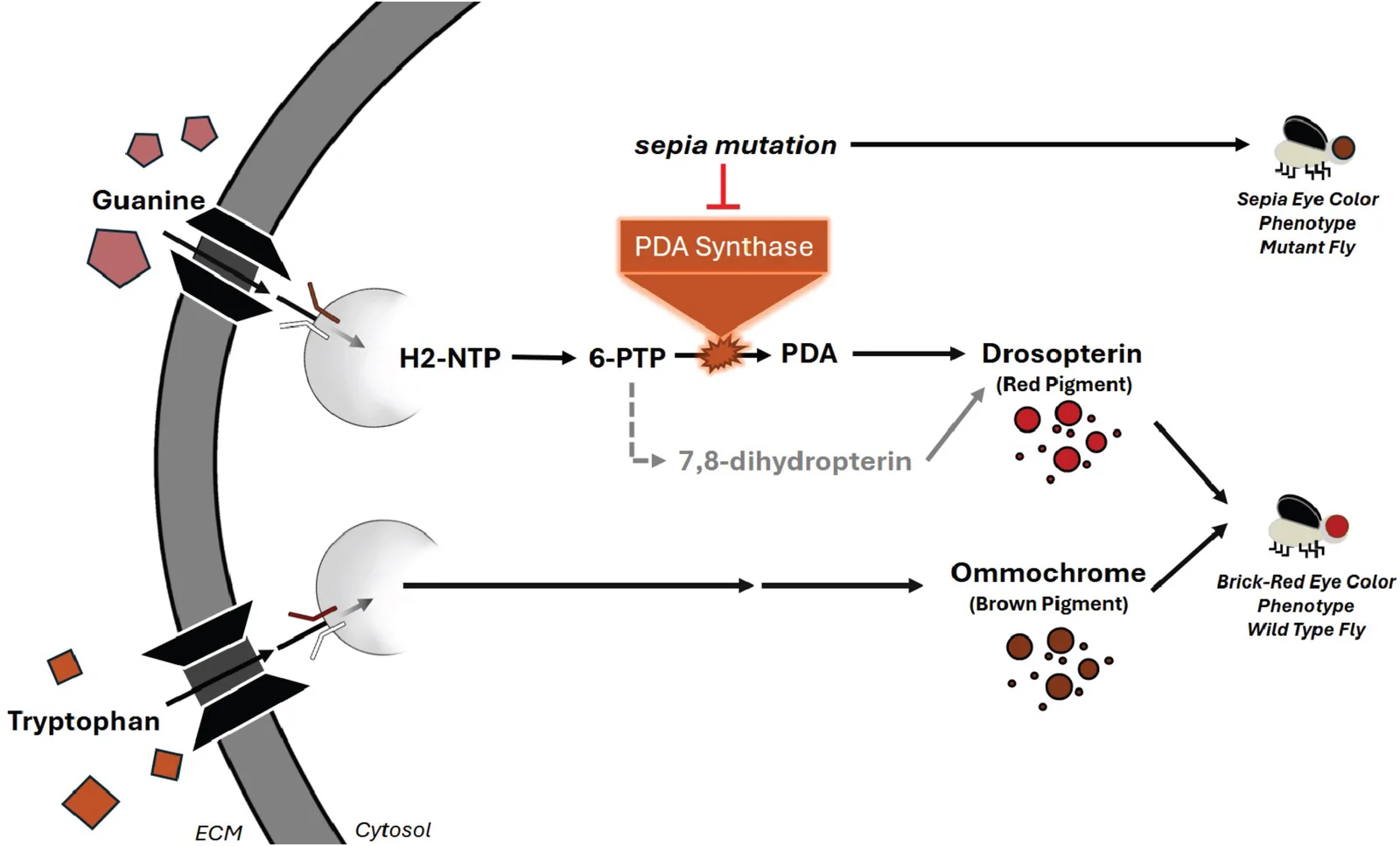
*Sepia* Gene Involvement in Biosynthesis of Eye Color Pigments. Red (drosopterin) and brown (ommochrome) pigments result in the brick-red eye color phenotype observed in wild-type flies. A *sepia* gene mutation in *D. melanogaster* resulting in a loss of PDA synthase function disrupts the production of red pigment, leading to a brown eye color phenotype [6,7].

**Fig 2:**
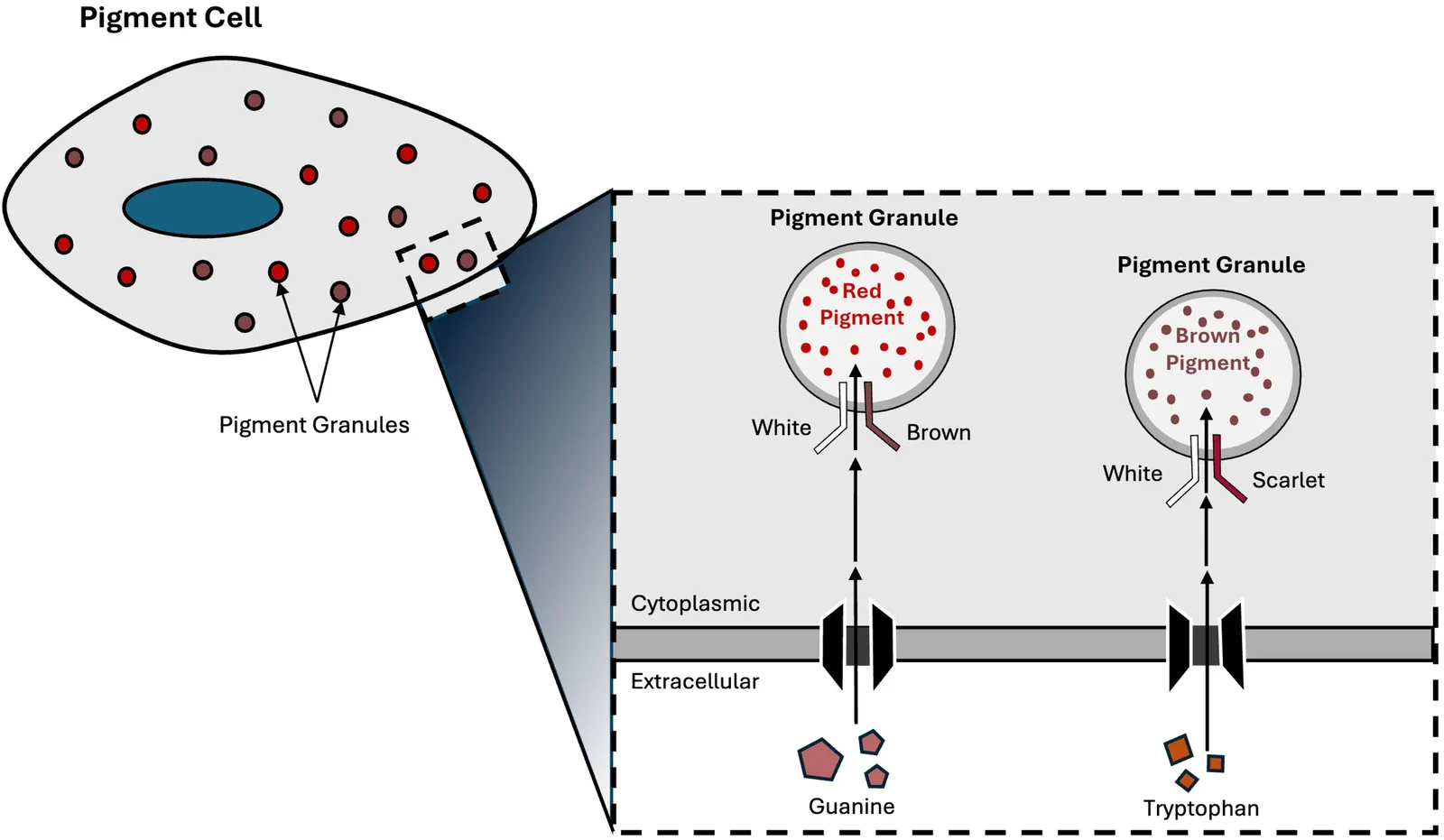
Role of White, Scarlet, and Brown Transport Proteins in Eye Pigment Production. In eye pigment cells, red and brown pigment granules contribute to the brick-red eye color phenotype observed in wild-type flies. The Brown transporter contributes exclusively to red pigment granules, while the Scarlet transporter contributes exclusively to brown pigment granules. The White transporter contributes to both brown and red pigment granules, and when non-functional, leads to an absence of both pigments and a white eye-color phenotype [8,9,12,13].

Eye color in the fly is determined by the formation of two types of pigments, ommochromes producing brown pigments and drosopterins producing red pigments [5,6]. The *sepia* gene (*se*, located on chromosome 3) encodes for the enzyme pyrimidodiazepine (PDA) synthase and impacts eye color in *D. melanogaster* as shown in Fig 1 [7]. PDA synthase produces a key intermediate in the drosopterin biosynthesis pathway, therefore, disruption of the *sepia* gene results in a mutant brown eye color phenotype due to the lack of red pigment production. However, the specific DNA mutation resulting in the sepia eye color phenotype is unknown for our fly population. The *white* gene (*w*, located on the X chromosome) encodes an ABC (ATP-binding cassette) transporter family protein which transports pigment precursors in the eye and was the first mutation described in *D. melanogaster* [5,8–13]. As shown in Fig 2, the loss of function of the *white* gene impacts both drosopterin and ommochrome pigment pathways in the fly, resulting in a mutant white eye phenotype. When amplifying genomic DNA to sequence and identify mutations responsible for the white-eyed phenotype, we discovered a promoter region insertion resembling a previously described *w1* mutant allele [14–17]. However, the exact location of the insertion is unclear. To elucidate this, we focus on investigating the 5’ and 3’ insertion junction sites in the 5’ untranslated region of the *white* allele.

**Fig 3:**
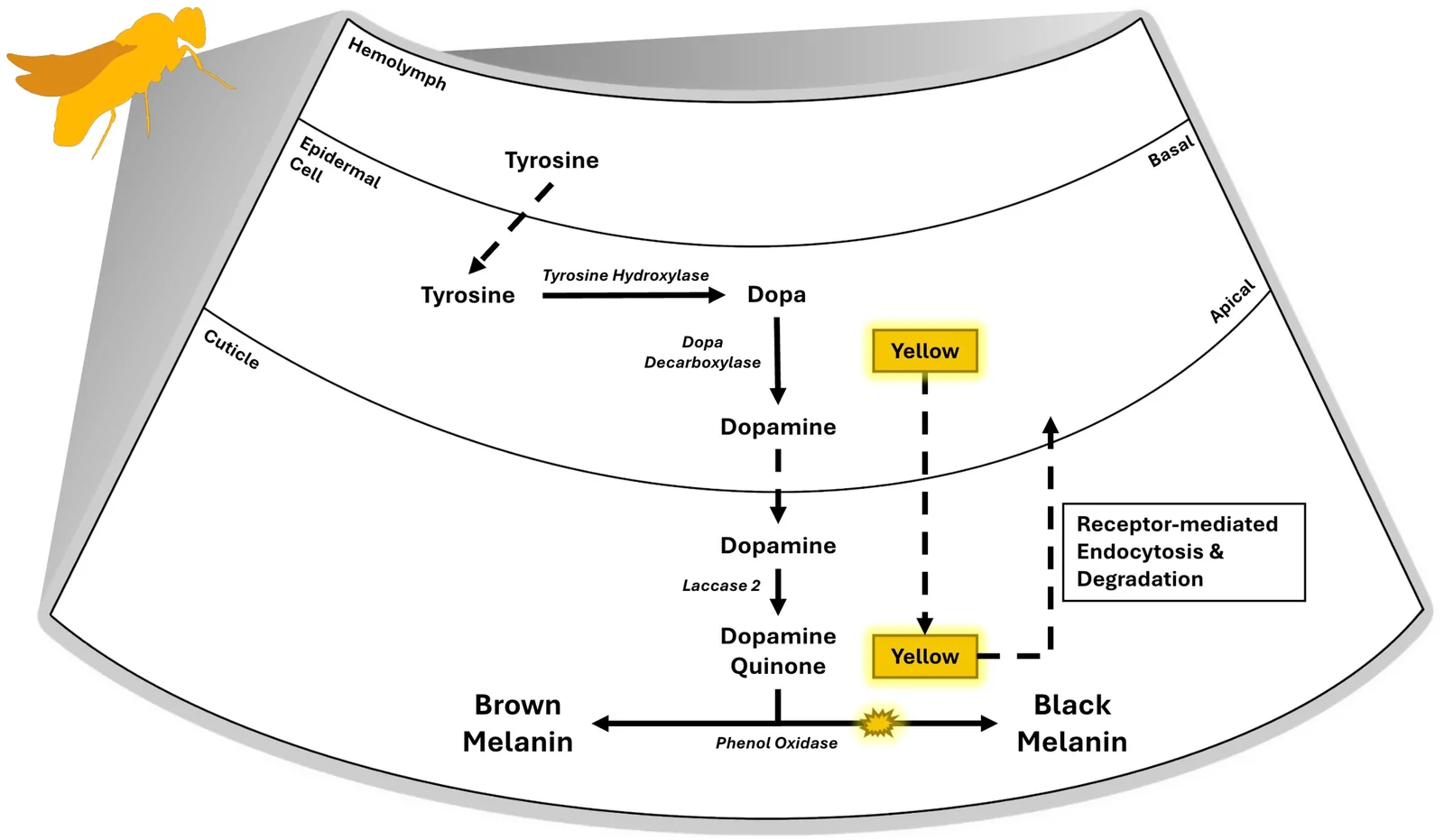
*Yellow* Gene Essential for Production of Black Melanin in Body Pigmentation Pathway. Black and brown melanin contribute to the wild-type body color phenotype. A mutation in the *yellow* gene involved in the black melanin pathway leads to a lack of black melanin production and a yellow body-color phenotype [19–21].

With respect to body pigmentation, it is determined by eumelanin (brown and black) and/or pheomelanin (yellow-tan or yellow-red) pigments [18]. In *D. melanogaster*, black melanin is produced by the Yellow protein and localized to the cuticle [19–21]. The *yellow* gene (*y*, located on chromosome X) is responsible for making the Yellow protein that is involved in body-color morphology. As shown in Fig 3, the Yellow protein is essential in producing black melanin, whereas a lack of Yellow protein results in a mutant yellow body color phenotype.

**Fig 4:**
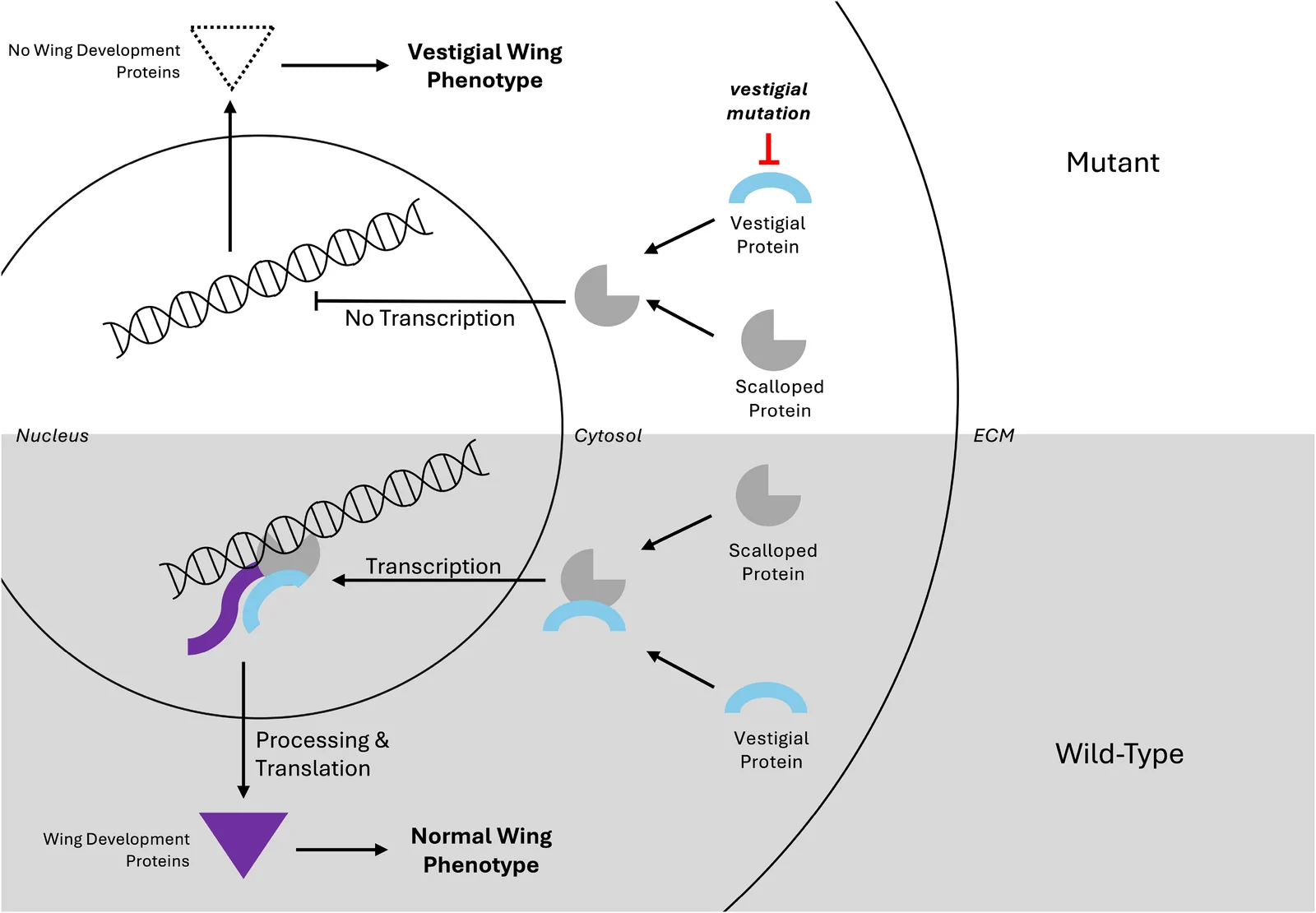
Vestigial Protein Regulates Wing Morphogenesis. The Vestigial protein, together with the Scalloped protein, forms a complex that translocates to the nuclear compartment to aid in the transcription and translation of wing development proteins for normal wing phenotype. A mutation in the **vestigial** gene leads to an absence of wing development proteins, resulting in a crumpled vestigial wing phenotype [22,24,25,29,30].

With respect to wing development, the *vestigial* gene (*vg*, located on chromosome 2) produces the Vestigial protein which forms a complex with the *scalloped* gene (*sd*) [22–25]. Both are essential contributors to wing development, as shown in Fig 4. When the *vestigial* gene is mutated, producing a non-functional Vestigial protein in *D. melanogaster*, it results in a crumpled wing vestigial phenotype. Apterous is a LIM-Homeodomain transcription factor that specifies dorsal and ventral identity important in early wing development in the fly [26–28]. A mutation in the *apterous* gene (*ap*, located on chromosome 2) leads to production of a non-functional Apterous protein and hence, a lack of wing development [29]. However, the specific mutation that contributes to this phenotype is not fully understood. This may be due to the large size of the Apterous protein, which may present challenges in amplifying the coding region. Therefore, the *apterous* gene remains a current project offering future research opportunities for undergraduate students interested in uncovering this genotype to phenotype connection.

**Fig 5:**
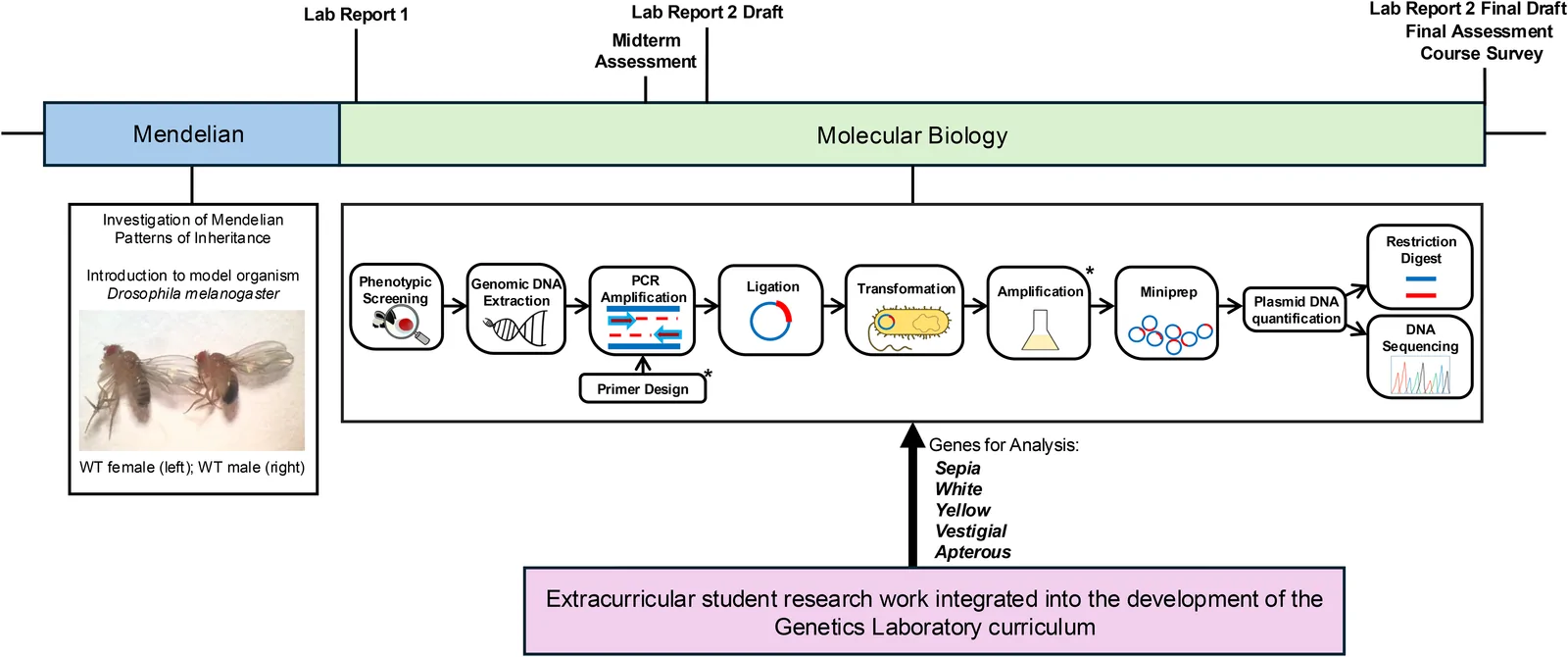
Genetics laboratory curriculum representation. A schematic representation of the Mendelian and Molecular Biology components of the Genetics Laboratory curriculum using *D. melanogaster* is depicted. The (*) indicates procedures that are not components of the course work but are performed by extracurricular research students as a part of their involvement in curriculum development.

In future iterations of the course, these above described genes (as well as others) will be applied on a rotational basis, while keeping the overall methods and techniques consistent each semester. For the project described in this paper, we placed emphasis on four genes in *D. melanogaster* that have been investigated by research students thus far, specifically *sepia*, *white*, *yellow*, and *vestigial*. At the beginning of each semester, students are introduced to *D. melanogaster* as a model organism. The students conduct phenotypic screening of a variety of mutant flies (i.e., *sepia*, *white*, *yellow*, *vestigial*, *apterous*, and *scalloped*) and engage in an experiment focused on Mendelian patterns of inheritance for the first 3–4 weeks of the course. During this part of the course, students learn how to handle, maintain, sex, and cross flies before transitioning into the Molecular Biology curriculum component. In the Molecular Biology component, from week 4 through the end of the course which represents greater than two-thirds of the course, students engage in uncovering DNA mutations responsible for mutant phenotypes in *D. melanogaster*. A visual summary of this curriculum is provided in a schematic presented in Fig 5. Our paper focuses on the Molecular Biology segment of this course, to report findings on the identity of DNA mutations in *sepia*, *white*, *yellow*, and *vestigial* genes, as well as to showcase the curriculum development that enhances the educational and research opportunities for students.

## Results

### Overall organization for curriculum development

**Fig 6:**
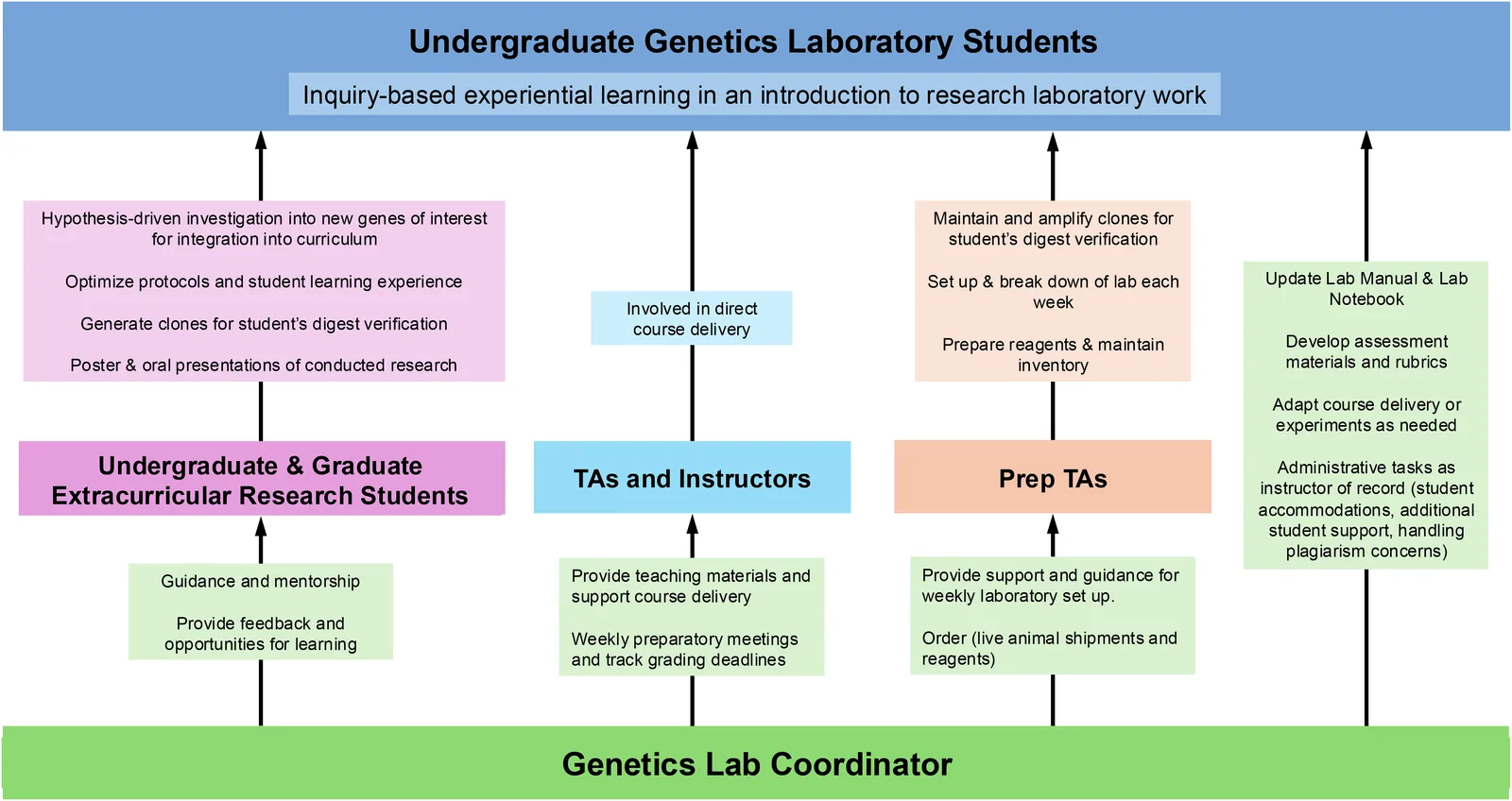
Roles in implementing curriculum updates. An overview of the roles involved in implementing curriculum updates for the undergraduate Genetics Laboratory students: Genetics Lab Course Coordinator, Extracurricular Research Students, Teaching Assistants (TAs) and Instructors, as well as Prep TAs.

To implement an inquiry-based experiential learning experience for students taking the undergraduate genetics laboratory at our institution, the Genetics Lab Coordinator worked with undergraduate and graduate extracurricular research students, TAs and Instructors, as well as Prep TAs (graduate students who are responsible for preparing reagents and getting the lab ready for each week) to update and improve the curriculum. This workflow is shown in Fig 6.

In addition to providing teaching materials, managing administrative tasks as instructor of record, and supporting course delivery, the work with extracurricular research students offered a unique opportunity to increase student involvement in curriculum development. Students interested in molecular biology research and education conducted hypothesis-driven investigations into new genes of interest for integration into the Genetics Laboratory course curriculum, optimized protocols and the student learning experience, as well as presented poster and oral presentations of conducted research.

Moving forward, the experimental approaches we (extracurricular research students, undergraduate Genetics Laboratory students, and the Genetics Lab Coordinator) performed, and their results are described in the sections below.

### Overall experimental approach

To uncover mutations that may inhibit gene expression, protein production, and/or function, we obtained mutant and wild-type *D. melanogaster* populations from Carolina Biological. We screened the flies for wild-type (WT) or mutant (MUT) phenotypes and harvested genomic DNA from each of these populations. When designing primers, we focused on amplifying the protein-coding regions for each gene of interest. For the *white* and *vestigial* genes, we designed primers for the 5’untranslated region (UTR) and some intronic sequence based on previously described mutations [14–17,24]. All original gel images are presented in S1 File. Concentration and purity of plasmid DNA isolated from the most recent *sepia*, *white*, and *vestigial* gene cloning experiments is presented in S2 File. Following sequencing of each gene, we gained further insight into the nature of the DNA mutations that may impact the phenotypic outcomes of the flies.

### Phenotyping and genomic DNA extraction

**Fig 7:**
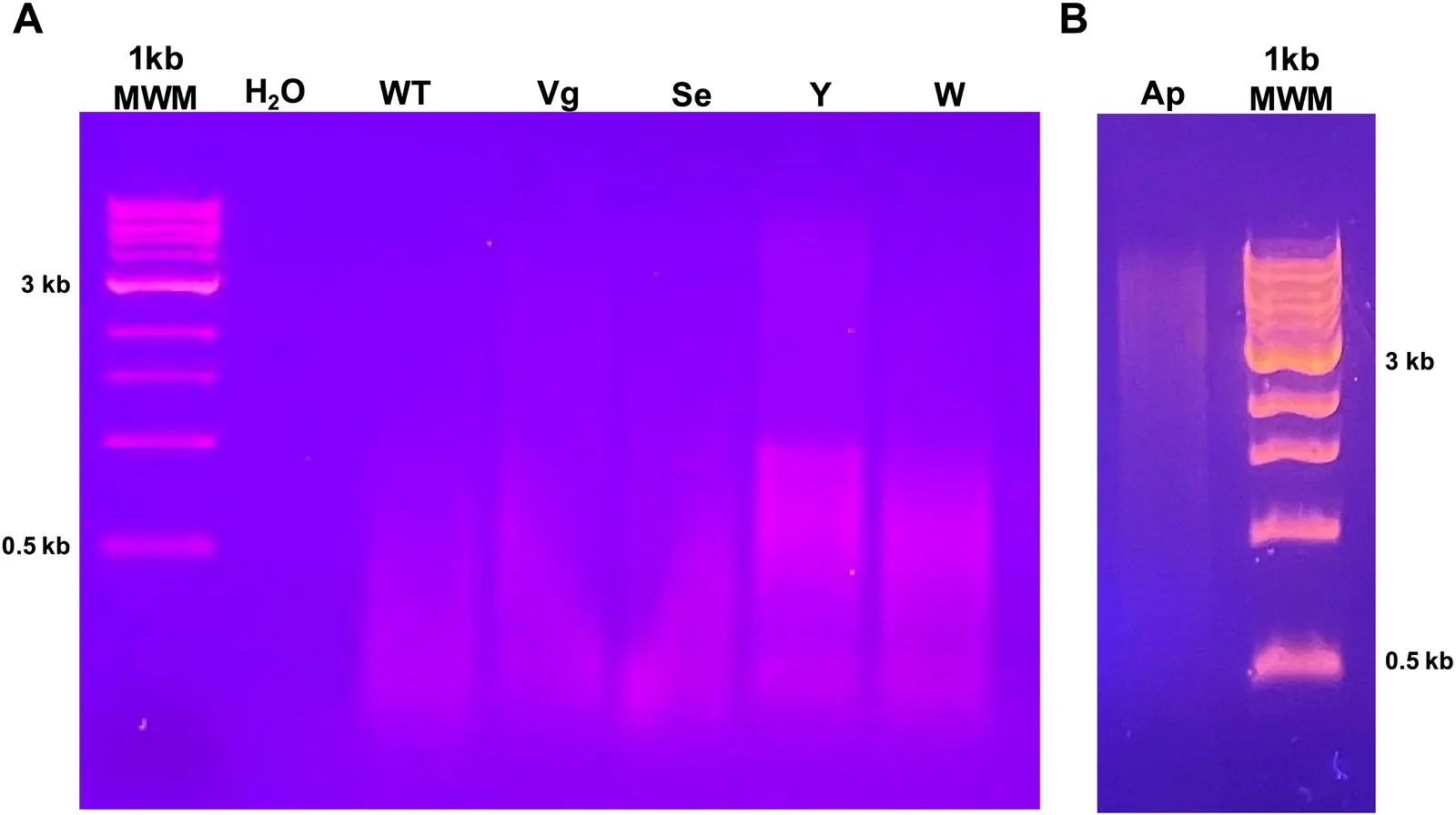
Isolation of genomic DNA. **A and B.** Verification of genomic DNA extracted from wild-type (WT) and mutant (Vg, Se, Y, W, Ap) flies is shown. All samples were treated with RNase. Molecular weight marker (MWM): Tri-Dye 1 kb DNA ladder is shown.

Genomic DNA extracted from WT and MUT (*sepia*, *white*, *yellow*, *vestigial*, or *apterous*) flies treated with ribonuclease (RNase) was confirmed via gel electrophoresis as shown in Fig 7.

### *Sepia* gene

**Fig 8:**
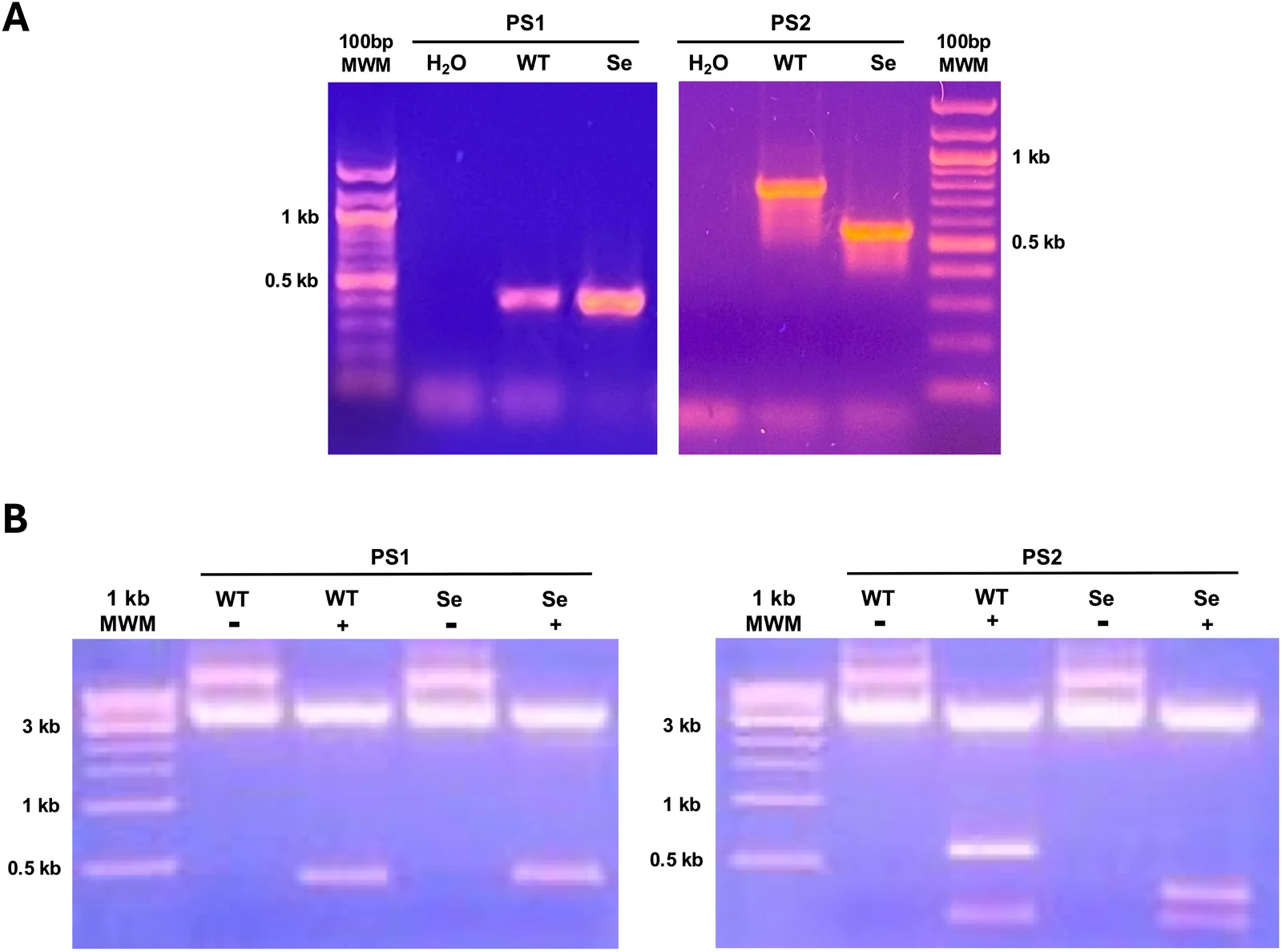
*Sepia* gene coding and digest gel. **A.** PCR amplification of *sepia* gene coding region from wild-type (WT) and MUT *sepia* (Se) genomic DNA. PS1 WT and MUT *sepia* (Se) fragments are expected sizes (412 bp). PS2 WT fragment shows the expected size (783 bp), while PS2 MUT *sepia* (Se) fragment shows a ~600 bp size band. **B.** EcoRI digest of TOPO Vector + PS1 insert (WT or Se PCR products shown above in A) displayed ~3 kb and ~400 bp bands. EcoRI digest of TOPO Vector + PS2 insert (WT PCR product shown above in A) displayed ~3 kb band as well as ~225 bp and ~558 bp bands (accounting for the internal cut site expected within the ~783 bp PCR product). TOPO + MUT *sepia* (Se) digested with EcoRI displayed a 3 kb band as well as ~225 bp and ~400 bp bands (accounting for the internal cut site and a smaller PCR product corresponding with the fragment size observed in A). H_2_O, WT, EcoRI cut (+), and EcoRI uncut (-) controls are shown. Molecular weight markers (MWM): Tri-Dye 100 bp and 1 kb DNA ladders are shown.

The genomic region targeted by primer set 1 (PS1) and primer set 2 (PS2), which span the coding region of the *sepia* gene, was PCR amplified, as shown in Fig 8A. PS1 showed expected sized PCR products for WT and *sepia* mutant (Se) samples at 412 bp. However, PS2 showed the expected sized PCR product for only the WT sample at ~783 bp. The *sepia* mutant (Se) sample using PS2 showed an approximately 600 bp sized PCR product across replicates (which is less than the expected ~783 bp) and was identified to be an appropriate target for genotyping this mutation in fly populations.

These resulting PCR products were ligated into TOPO vectors by extracurricular research students to generate plasmids for use in the laboratory course. The resulting recombinant plasmids were subjected to EcoRI restriction digestion. The results shown in Fig 8B verified the presence of the WT and *sepia* mutant inserts. There are no internal EcoRI cut sites for the PS1 expected product. In support, we observed that the recombinant plasmid digested with EcoRI displayed a ~400 bp band which represents the PS1 insert along with the ~3 kb band for the vector. For the PS2 expected product, there is one internal EcoRI cut site at ~225 bp within the insert. For PS2, the WT sample showed a ~558 bp band and a ~225 bp band to account for the insert (~783 bp) with the cut site, along with the ~3 kb band for the vector. However, the *sepia* mutant sample displayed a ~400 bp band (smaller by ~200 bp compared to the WT) and a ~225 bp band, along with the ~3 kb for the vector as shown in Fig 8B. This smaller fragment observed in PS2 for the *sepia* mutant aligns with the smaller than expected sized product observed from the PCR amplification described above.

**Fig 9:**
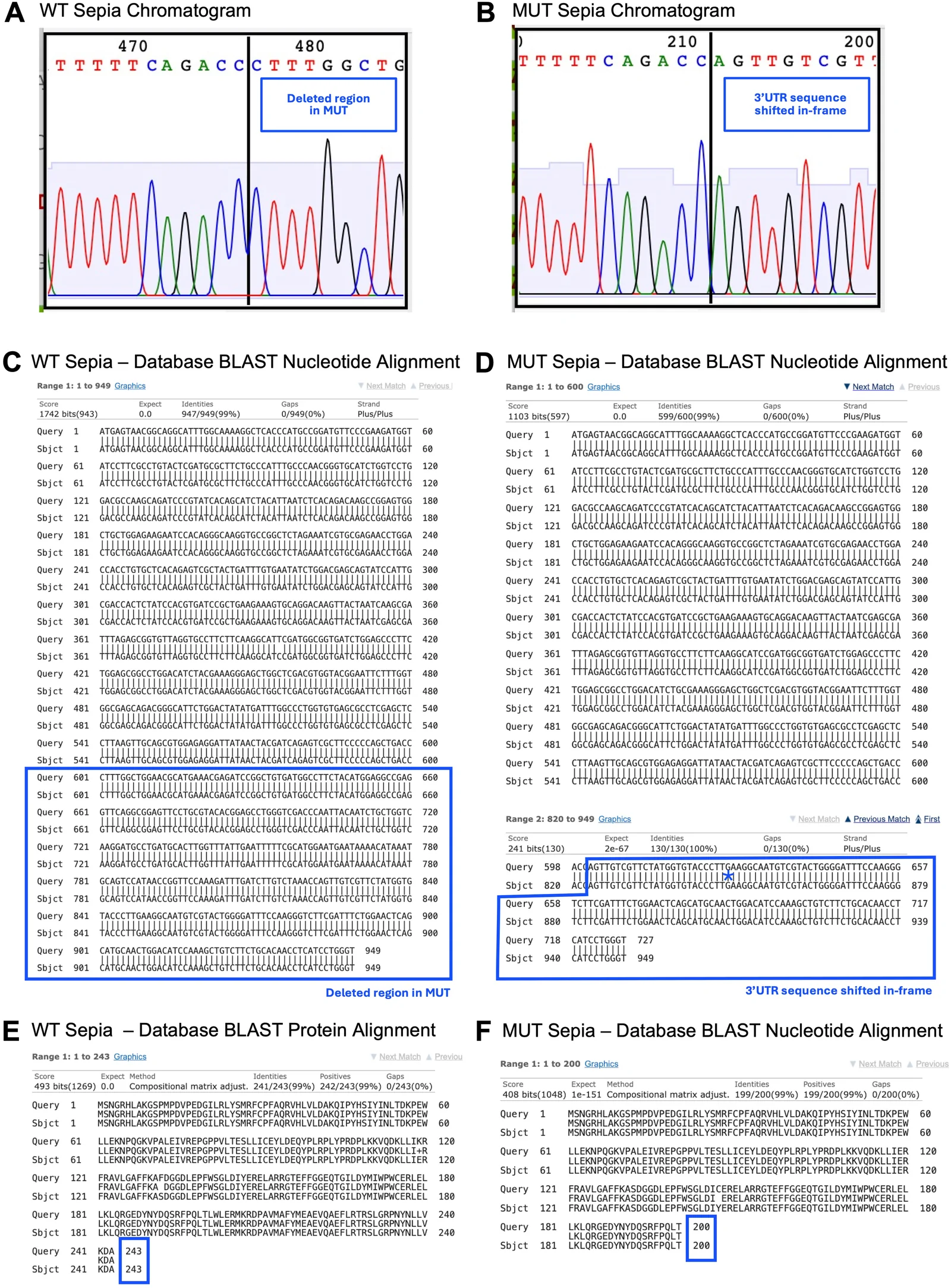
*Sepia* gene sequence analysis. **A.** WT *sepia* chromatogram at the deletion site indicated with the black line (sequence to the right is the deleted region in MUT). **B.** MUT *sepia* chromatogram at the deletion site indicated with the black line (sequence to the right from 3’UTR placed in-frame). **C.** Compiled WT *sepia* cDNA sequence aligned with database consensus sequence in BLAST nucleotide analysis, blue box highlighting the region that is deleted in the MUT *sepia* sequence. **D.** Compiled MUT *sepia* cDNA sequence aligned with database consensus sequence in BLAST nucleotide analysis showing the deleted region and a blue box following the truncation displaying the additional 126 nucleotides from 3’UTR sequence, with (*) indicating new stop codon. **E.** WT Sepia protein sequence aligned with database using BLAST protein analysis showing 243 amino acids for the full-length protein. **F.** MUT Sepia protein sequence aligned with database in BLAST protein analysis showing 200 amino acids aligned with the database sequence. The additional 8 amino acids (N-SCRSMVYP-C) placed in frame following the truncation are not visible in this alignment with database sequence.

Raw sequence results were analyzed from which a compiled complementary DNA (cDNA) sequence was generated for both WT *sepia* and MUT *sepia*, which is provided in S3 File. The WT *sepia* expected nucleotide sequence is shown to the right of the black line in Fig 9A. This sequence is not preserved in MUT *sepia*, resulting in 3’UTR sequence shifted in-frame shown to the right of the black line in Fig 9B. In Fig 9C, 348 nucleotides (#601–949, shown with a blue box in the WT *sepia* sequence) are absent in the compiled MUT *sepia* sequence in Fig 9D. Furthermore, in Fig 9D, an additional unique 126 nucleotides (#601–727, shown with a blue box in the MUT *sepia* sequence) were noted. This corresponds to the protein alignment in Fig 9E and Fig 9F, where the WT Sepia protein sequence contains the full expected 243 amino acids whereas the MUT Sepia protein sequence contains 208 amino acids. Of these 208 amino acids shown in Fig 9F, 200 amino acids are preserved from database consensus sequence obtained from Ensembl genome browser matching the expected WT Sepia sequence displayed in Fig 9E, and 8 amino acids are newly encoded [31]. These 8 newly encoded amino acids (N-SCRSMVYP-C) are from the 3’UTR sequence, and therefore they are not present in the database consensus sequence and do not appear in the alignment presented.

### *White* gene

**Fig 10:**
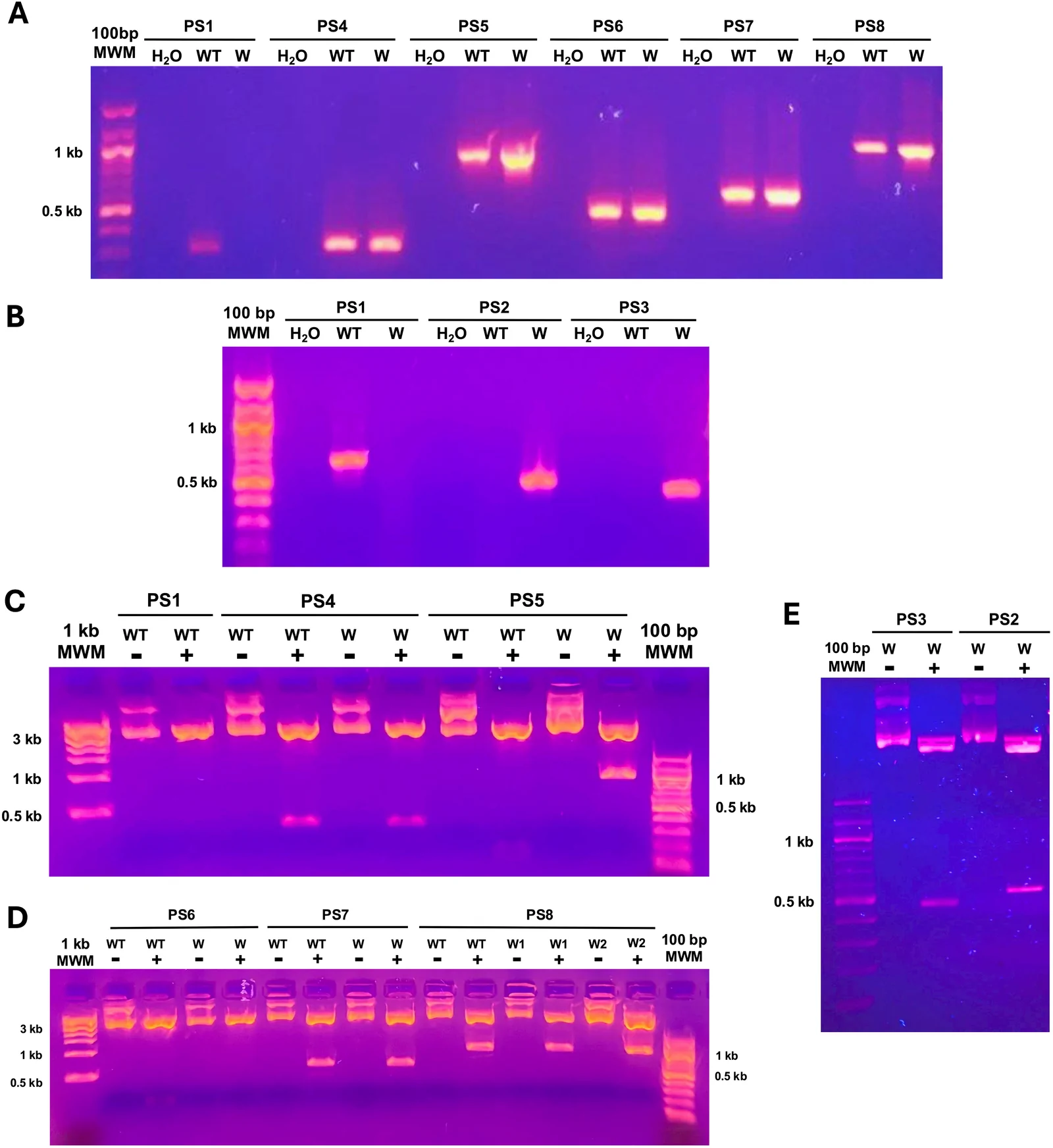
*White* gene coding and digest gel. **A.** PCR amplification of coding region from WT and MUT *white* genomic DNA shows absence of exon 1 (PS1) product from MUT *white* (W). Exon 4 fragment (PS6) results in ~600 bp band (larger than the expected ~435 bp size band) for both WT and MUT *white* (W). **B.** Amplification of the insertion junction site region for WT (PS1), 5’ junction for W (PS2) and 3’ junction site for W (PS3) display ~640 bp, ~500 bp, ~443 bp bands, respectively. **C, D, and E.** EcoRI digest of plasmids obtained by transformation/mini prep to verify presence of gDNA amplified inserts. H_2_O, WT, EcoRI cut (+), and EcoRI uncut (-) controls are shown. Molecular weight markers (MWM): Tri-Dye 100 bp and 1 kb DNA ladders are shown.

Primer set 1 (PS1) and primer sets 4–8 (PS4-PS8) spanning the coding region of the *white* gene were utilized to PCR amplify products as shown in Fig 10A. PS4-PS8 PCR products showed identical sized expected bands for both WT and *white* mutant (W) samples, with the exception of PS6. PS6, which amplifies exon 4, resulted in a larger (~600 bp) than expected size (435 bp, as noted in Table 1 in Materials and Methods) sized fragment for both WT and W. Primer set 1 (PS1) successfully amplified the WT sample and *white* mutant sample (W) as shown in both Fig 10A and Fig 10B. In Fig 10B, use of primer sets PS2 and PS3 covering the 5’ and 3’ insertion junction sites displayed an expected product for WT and W.

**Table 1:** Primer sets and sequences for *Sepia*, *White*, *Yellow*, and *Vestigial* genes.

***sepia* Gene**				
*Primer Set*	*Primers*	*Primers sequence (5’-3’)*	WT expected product size (bp)	MUT expected product size (bp)
PS1	*se* F1	CACTCGAATGACAATCGCTATC	412	783
	*se* R1	CAATGGATACTGCTCGTCCAG		
PS2	*se* F2	CTGTGCTCACAGAGTCGCTAC	412	783
	*se* R2	CTCAAGTGGCGCGATTTGATG		
***white* Gene**				
*Primer Set*	*Primers*	*Primers sequence (5’-3’)*	WT expected product size (bp)	MUT expected product size (bp)
PS1	*w* P1*	GTGCAAAGGTGGTCGAATTT	640	-
	*w* R1*	GTATGCACATGTACTACTCAC		
PS2	*w* P1*	GTGCAAAGGTGGTCGAATTT	-	500
	*w* docR1	CTGCACTTTACACTTTGTGT		
PS3	*w* docF1	GCCCAGCAATAAACTTATTAGGG	-	443
	*w* R1*	GTATGCACATGTACTACTCAC		
PS4	*w* F2	GAGATCCCCTATCATTGCAG	315	315
	*w* R2	CGACTGCGAATAGAAACTCAC		
PS5	*w* F3	CGGACACGCGGACTATTCTG	758	758
	*w* R3	ATTGTAGTTGGTAGGACACTG		
PS6	*w* F4	GACAAGATCCTTCTGATGGCC	435	435
	*w* R4	GTTTCCACTGGAACCACTCAC		
PS7	*w* F5	GAATGGGTACACCTACAAGGC	307	307
	*w* R5	GCGATAAAGTCGACTTCGGG		
PS8	*w* F6	TGGGTGTGATGAATATCAACG	848	848
	*w* R6	CACAAGTAGACTTTGGATTTG		
***yellow* Gene**				
*Primer Set*	*Primers*	*Primers sequence (5’-3’)*	WT expected product size (bp)	MUT expected product size (bp)
PS1	*y* F1	GCCGACATATTATGGCCACC	886	886
	*y* R1	CAATGCCATGCTATTGGCTTC		
PS2	*y* F2	GTTTGGCCCTGCTAATTCTCC	621	621
	*y* R2	GTCGATGACTTGCTAACGGAC		
PS3	*y* F3	GATGGTTATCGTACCCTGTAC	875	875
	*y* R3	ACCCAAGTACCGTGTGTAGG		
***vestigial* Gene**				
*Primer Set*	*Primers*	*Primers sequence (5’-3’)*	WT expected product size (bp)	MUT expected product size (bp)
PS1	*vg* F1	CCGAGCGGAGTTTTCATTCC	632	632
	*vg* R1	GTTCGCTCGAGGAGTTAGCC		
PS2	*vg* F2	GGGAGCGTGGTTAAAGAAGC	785	785
	*vg* R2	CTAGATCGCCATCATTAGGGATG		
PS3	*vg* F3	CGAAGCACTCGCATTCCAAAC	547	547
	*vg* R3	TCACAACTCACTGGAGCCAC		
PS4	*vg* F4	CGCCCTGTCTGATTTCCCAG	714	714
	*vg* R4	TGGTAAGCATGCTGATTATCCT		
PS5	*vg* F5**	CCCTCATTTGGCAATTGCAG	644	644
	*vg* R5	AACCCTGCTCATAGGCTCAC		
PS6	*vg* F6	GCCGGCTTCTCGTGTCTTAC	533	533
	*vg* R6**	GCCGAAGGACATCGGCGATC		
PS7	*vg* F5**	CCCTCATTTGGCAATTGCAG	1073	1073
	*vg* R6**	GCCGAAGGACATCGGCGATC		
PS8	*vg* ZF***	AAGAAGAGGACCTCATCGTG	409	409
	*vg* ZR***	TGGCCTAACTGCGTGCATAA		
PS9	*vg* ZF***	AAGAAGAGGACCTCATCGTG	766	-
	*vg* R9	CTGGGAAATCAGACAGGGCG		
PS10	*vg* ZF***	AAGAAGAGGACCTCATCGTG	823	-
	*vg* R10	CCGGAGGAGCAGGATGATTC		
PS11	*vg* ZF***	AAGAAGAGGACCTCATCGTG	825	-
	*vg* R11	CTCCAGTGCTGGCAGATTC		

*w* P1 and *w* R1 primers notated with (*) have been designed and reported on in previous studies [17]. *vg* F5 and *vg* R6 primers notated with (**) are used in multiple *vestigial* gene primer sets (PS5, PS6, and PS7). *vg* ZF and *vg* ZR notated with (***) have been designed and reported on in previous studies, originally named ‘vg exon 3’ and ‘LTR 412’, respectively [35].

**Fig 11:**
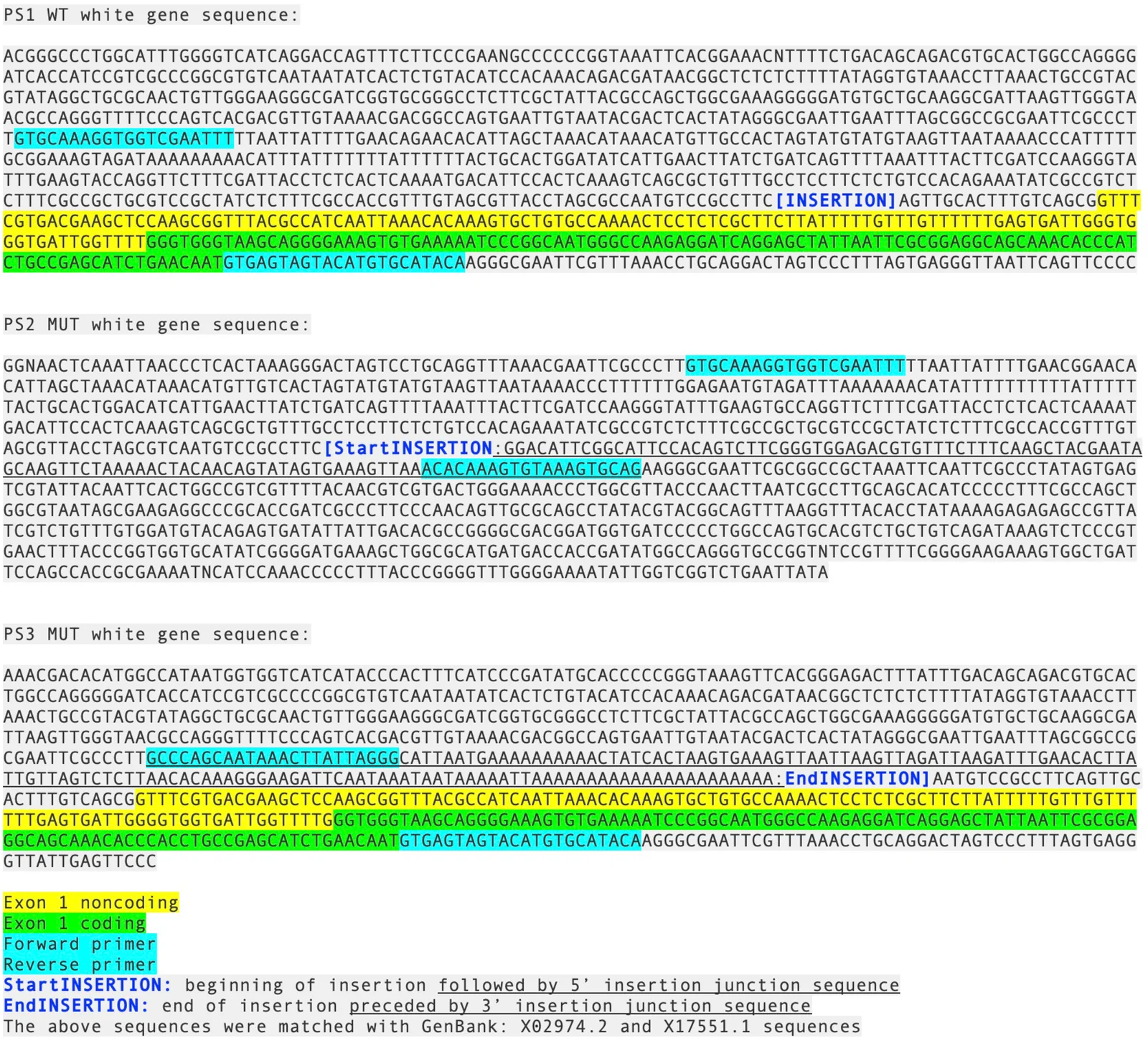
*White* Gene Sequence Analysis. Primer Set 1 (PS1) amplified and sequenced region of the WT *white* gene noting the region of insertion. Primer Set 2 (PS2) and Primer Set 3 (PS3) amplified and sequenced regions of the MUT *white* gene indicating the start and end of the Doc retrotransposon insertion, respectively.

The recombinant plasmids (i.e., amplified PCR products, using PS1-PS8 primer sets, cloned into TOPO vector) were digested with EcoRI to verify the presence of inserts. Fig 10C, Fig 10D, and Fig 10E display the results from EcoRI restriction digestion. For any samples not verified to contain appropriately sized inserts on these gels, additional replicates were digested until verified to send for sequencing. All raw sequences provided validation that the appropriate regions were amplified, with the exception of PS6. PS6, which targets the exon 4 region of the *white* gene, was not successfully cloned or sequenced. PS1, which covers the 5’UTR and exon 1 segment is compared to PS2 and PS3, which cover the 5’ and 3’ Doc retrotransposon junction sites, as shown in Fig 11.

### *Yellow* gene

**Fig 12:**
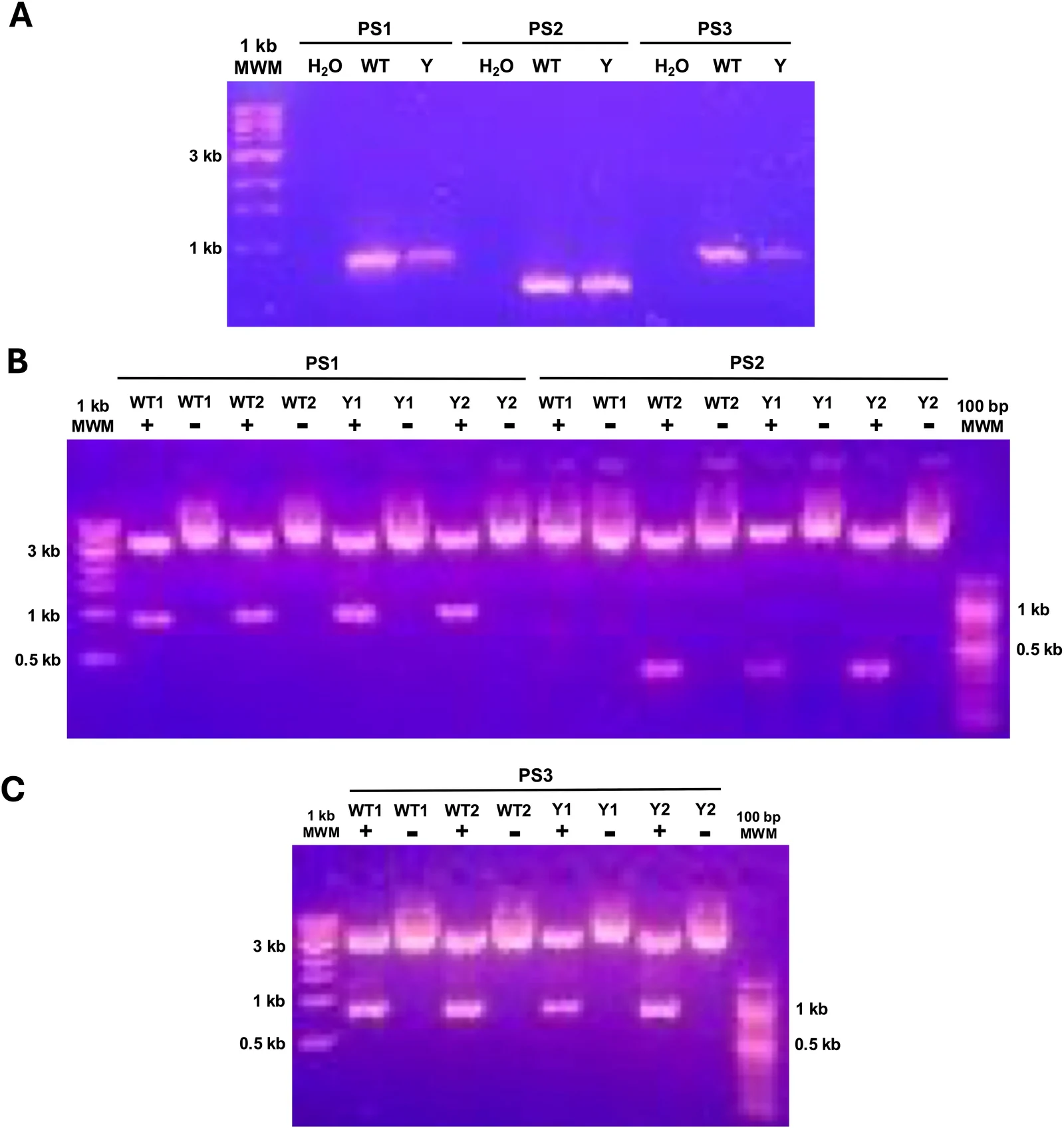
*Yellow* gene coding and digest gels. **A.** PCR amplification of the *yellow* gene coding region (PS1, PS2, and PS3), shows expected size WT and MUT *yellow* (Y) products for each primer set (886 bp, 621 bp, and 875 bp, respectively). **B and C.** EcoRI digest of samples verify expected inserts prior to sequencing. H_2_O, WT, EcoRI cut (+), and EcoRI uncut (-) controls are shown. Molecular weight markers (MWM): Tri-Dye 100 bp and 1 kb DNA ladders are shown.

Primer sets 1–3 (PS1-PS3) were utilized to amplify the coding region of the *yellow* gene as shown in Fig 12A. PCR amplified products displayed the expected sized fragments across all samples. Following cloning into the TOPO vector to generate recombinant plasmids, EcoRI digestion verified the presence of expected sized bands on agarose gels, as shown in Fig 12B and Fig 12C.

**Fig 13:**
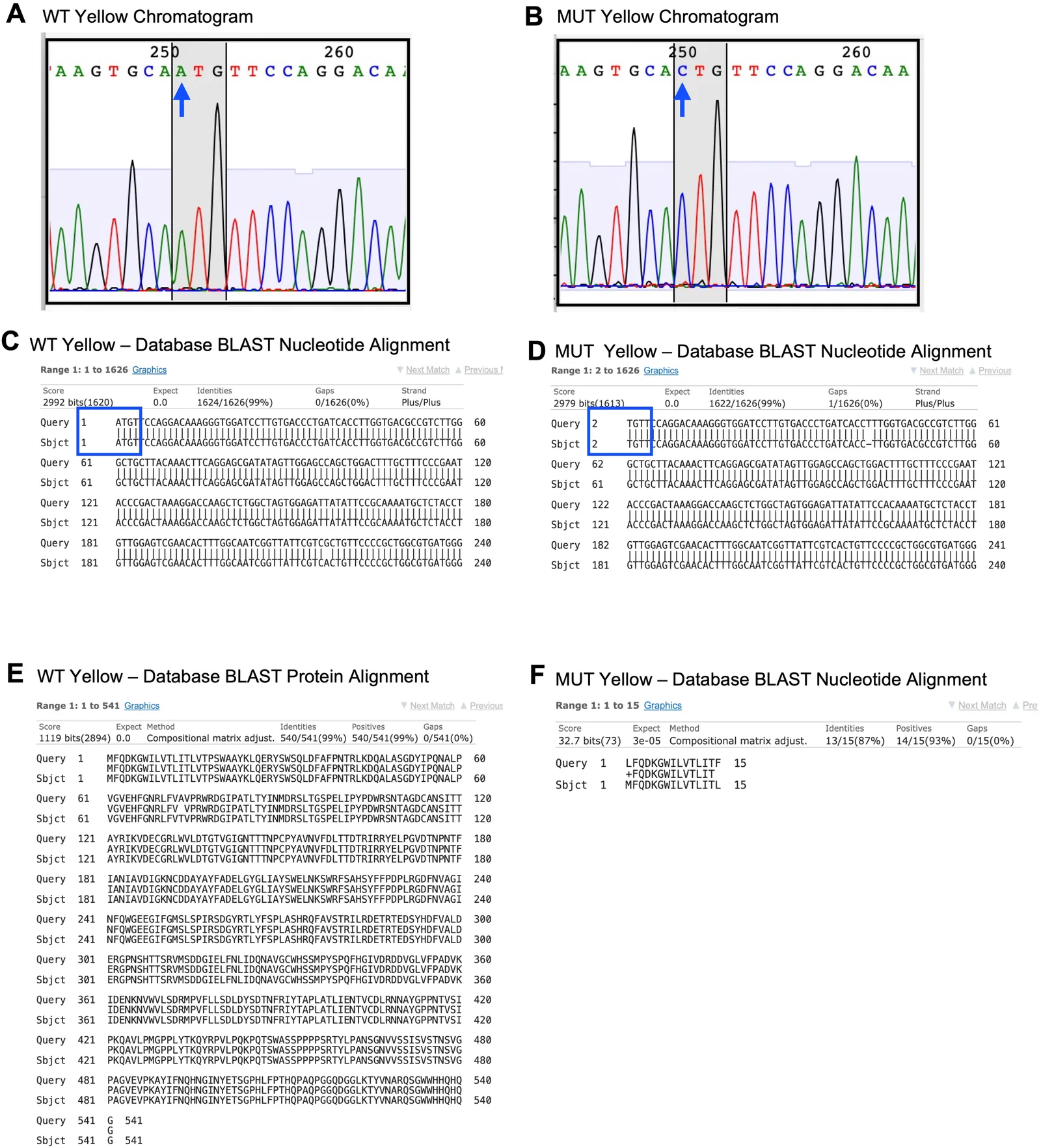
*Yellow* gene sequence analysis. **A.** WT *yellow* chromatogram showing the ATG start codon (highlighted). **B.** MUT *yellow* chromatogram showing the CTG (highlighted). Blue arrows indicate the A to C point mutation responsible for disrupting the start codon. **C.** Compiled WT *yellow* cDNA sequence aligned with database consensus sequence in BLAST nucleotide analysis, blue box indicating the alignment starting at Query and Sbjct #1 beginning at the ATG start codon. **D.** Compiled MUT *yellow* cDNA sequence aligned with database consensus sequence in BLAST nucleotide analysis, blue box indicating alignment starting at Query and Sbjct #2 due to the A to C point mutation. **E.** WT Yellow protein sequence aligned with database in BLAST protein analysis showing 541 amino acids for the full-length protein. **F.** MUT Yellow protein sequence aligned with database in BLAST protein analysis showing a lack of alignment with the database sequence.

Raw sequence results were compared to the database sequence obtained from Ensembl genome browser [31]. A compiled cDNA sequence was then generated for WT *yellow* and MUT *yellow*, as shown in S4 File. The chromatogram for WT *yellow* identifies the ATG start codon as shown in Fig 13A, while the MUT *yellow* chromatogram sequence shows an A to C point mutation in the start codon indicated by the blue arrow as shown in Fig 13B. The WT *yellow* and MUT *yellow* cDNA sequences were aligned to database consensus sequence derived from Ensembl genome browser as shown in Fig 13C and Fig 13D. From this analysis, the MUT *yellow* cDNA sequence shows the lack of an A at the query #1 position. In Fig 13E, Protein BLAST alignment results show the full 541 amino acids for WT, whereas the mutant protein BLAST shows a lack of alignment to the database protein sequence.

### *Vestigial* gene

**Fig 14:**
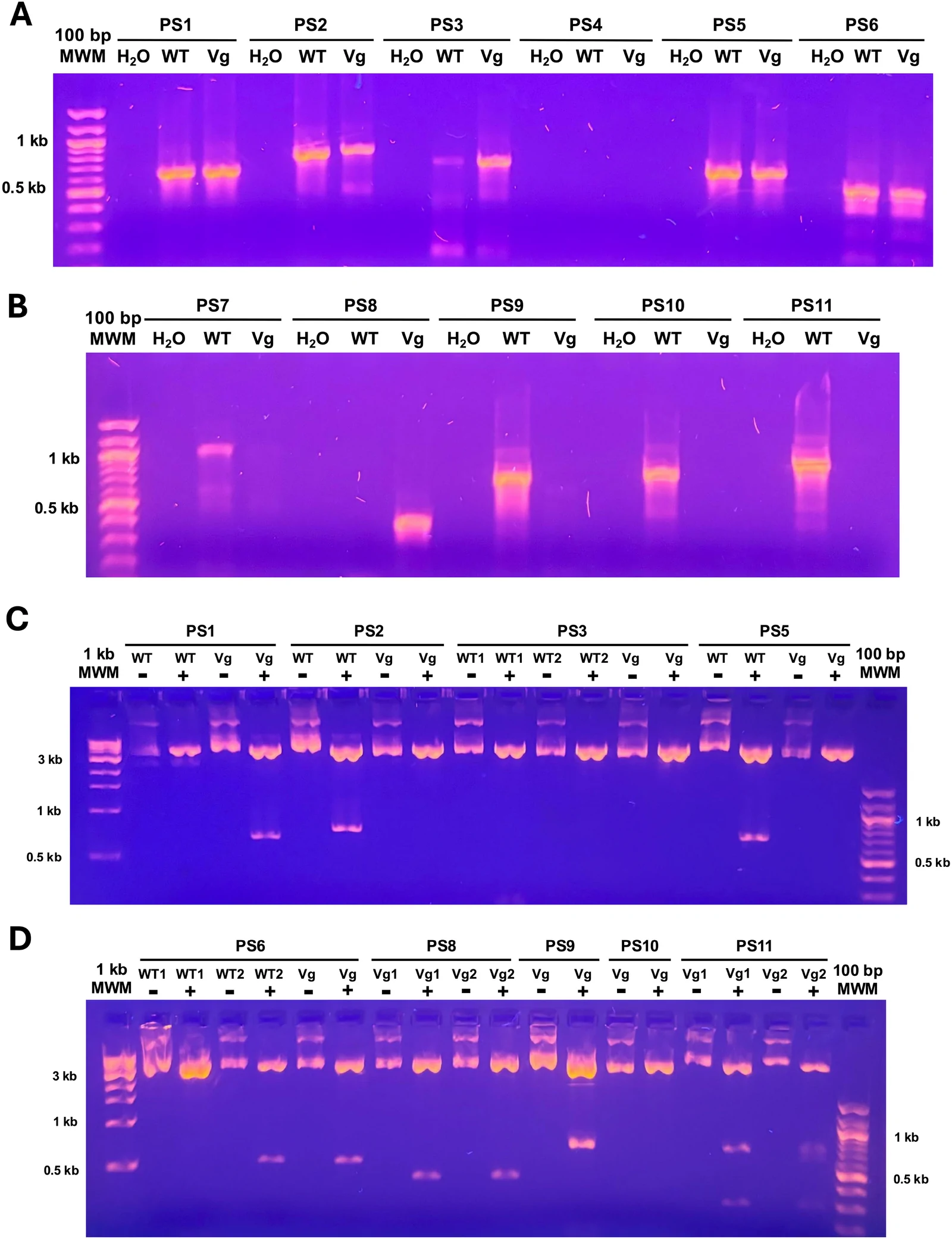
*Vestigial* coding and digest gels. **A and B.** PCR amplification of the *vestigial* gene coding region (PS1-PS11) shows the expected size fragments as noted in Table 1. PS4 was not successfully amplified and PS3 spanning the same region on the *vestigial* gene was used to move forward with the cloning process. PS8-PS11 targeting the insertion site in intronic sequence between exon 3 and 4 show expected size fragments (~409 bp, ~766 bp, ~823 bp, and ~825 bp, respectively). **C and D.** EcoRI digest shows verification of samples with PCR amplified inserts. H_2_O, WT, EcoRI cut (+), and EcoRI uncut (-) controls are shown. Molecular weight markers (MWM): Tri-Dye 100 bp and 1 kb DNA ladders are shown.

As shown in Fig 14A and Fig 14B, primer sets 1–7 (PS1-PS7) were utilized to amplify the coding region of the *vestigial* gene, whereas primer sets 8–11 (PS8-PS11) amplified the 412-retrotransposon insertion region. Primer set 4 (PS4) did not successfully amplify the exon 4 region. Primer set 3 (PS3), expected to improve success in amplifying the exon 4 region, initially showed nonspecific products (as displayed in Fig 14A). However, upon PCR optimization, the use of PS3 generated specific PCR products that enabled recombinant clone verification via restriction enzyme digest. While primer set 7 (PS7) displayed the expected size product (~1073 bp) for WT and *vestigial* mutant (Vg) samples, there was amplification of non-specific products as well. Primer set 5 (PS5) which is expected to PCR amplify a similar region of the *vestigial* gene as PS7 showed expected sized PCR products. Therefore, the entire coding region of the *vestigial* gene was amplified through multiple replicates, primer-pair combinations, and optimization of PCR settings.

**Fig 15:**
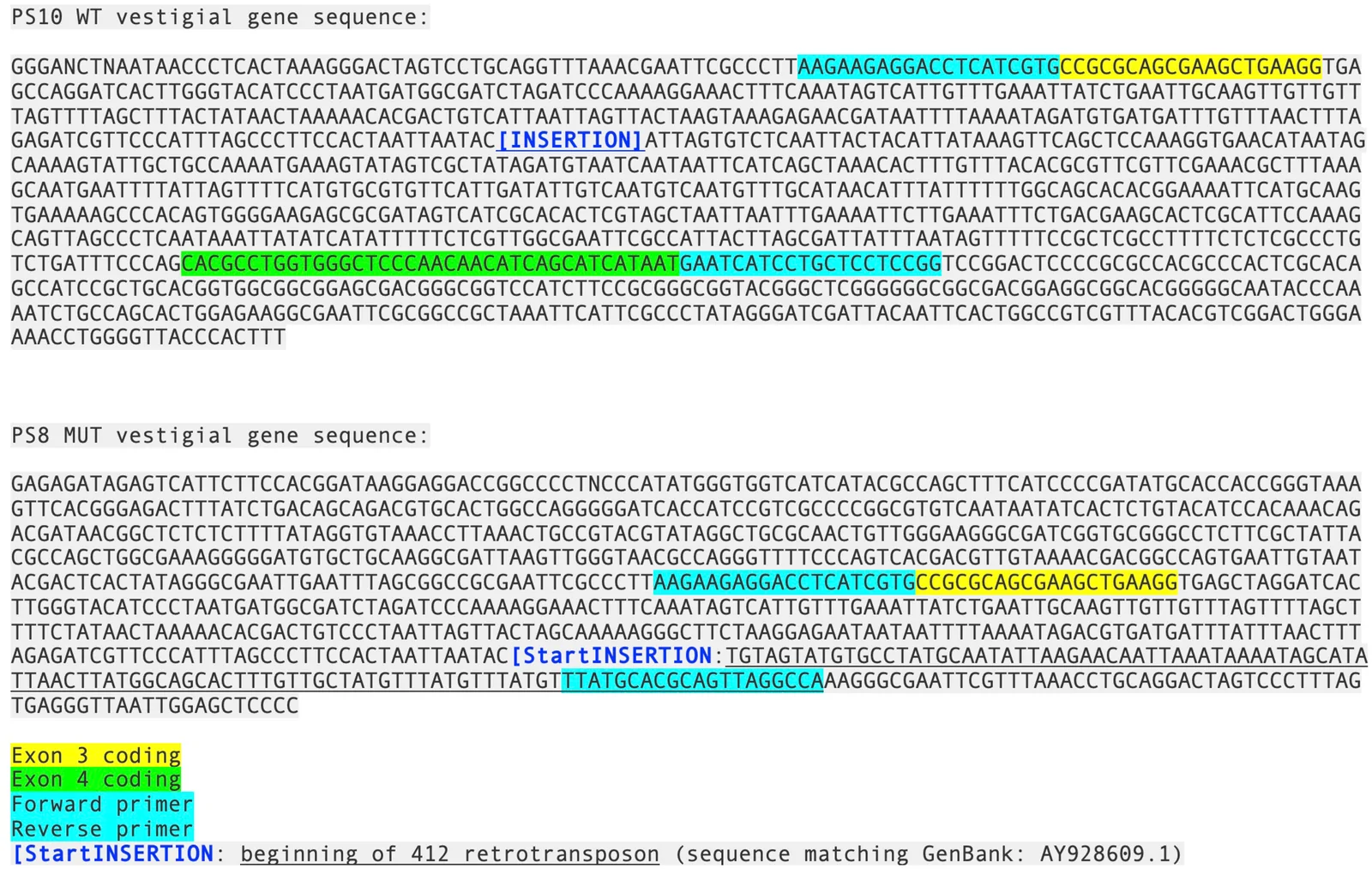
*Vestigial* gene sequence analysis. Primer Set 10 (PS10) amplified and sequenced region of the WT *vestigial* gene noting the region of insertion. Primer Set 8 (PS8) amplified and sequenced the 5’ insertion junction region in the MUT *vestigial* gene, displaying the beginning of the 412 retrotransposon.

As shown in Fig 14C and Fig 14D, recombinant TOPO plasmids digested with EcoRI displayed expected insert and vector band sizes, which enabled validation via sequencing. Due to the large number of samples, multiple replicates supported the sequencing of our regions of interest. PCR amplification utilizing primer set 10 (PS10), designed to bind to exon 3 and exon 4 of the *vestigial* gene near the predicted insertion region, displayed the expected size product for only the WT sample as anticipated. This data was compared to the raw sequencing data derived from the MUT *vestigial* gene, using primer set 8 (PS8), as shown in Fig 15. Comparison of these sequences identified similarity between WT *vestigial* and MUT *vestigial* in exon 4 up to the 5’ insertion junction region.

### Student engagement and outcomes in the genetics laboratory program

Students enrolled in the General Genetics laboratory course complete a Lab Report draft (including the title page, introduction, materials and methods, as well as references) three to four weeks into the Molecular Biology component of the curriculum as shown in Fig 5. After receiving feedback, students continue to work on updating their lab report as they conclude their experiments. At the end of the course, students submit a Full Lab Report which now includes their results and discussion sections. The draft and full lab report guidelines and rubrics are presented in S5 File.

In recent years, a maximum of 432 students each semester (24 students across 18 sections) engage in this experimental design. Student grade distributions from Fall 2023, Spring 2024, Summer 2024, and Fall 2024 revealed that greater than 95% students passed the course with a letter grade C or better, with a majority of students receiving a letter grade of B or better.

### Program evaluation – student perspectives of the genetics laboratory

**Fig 16:**
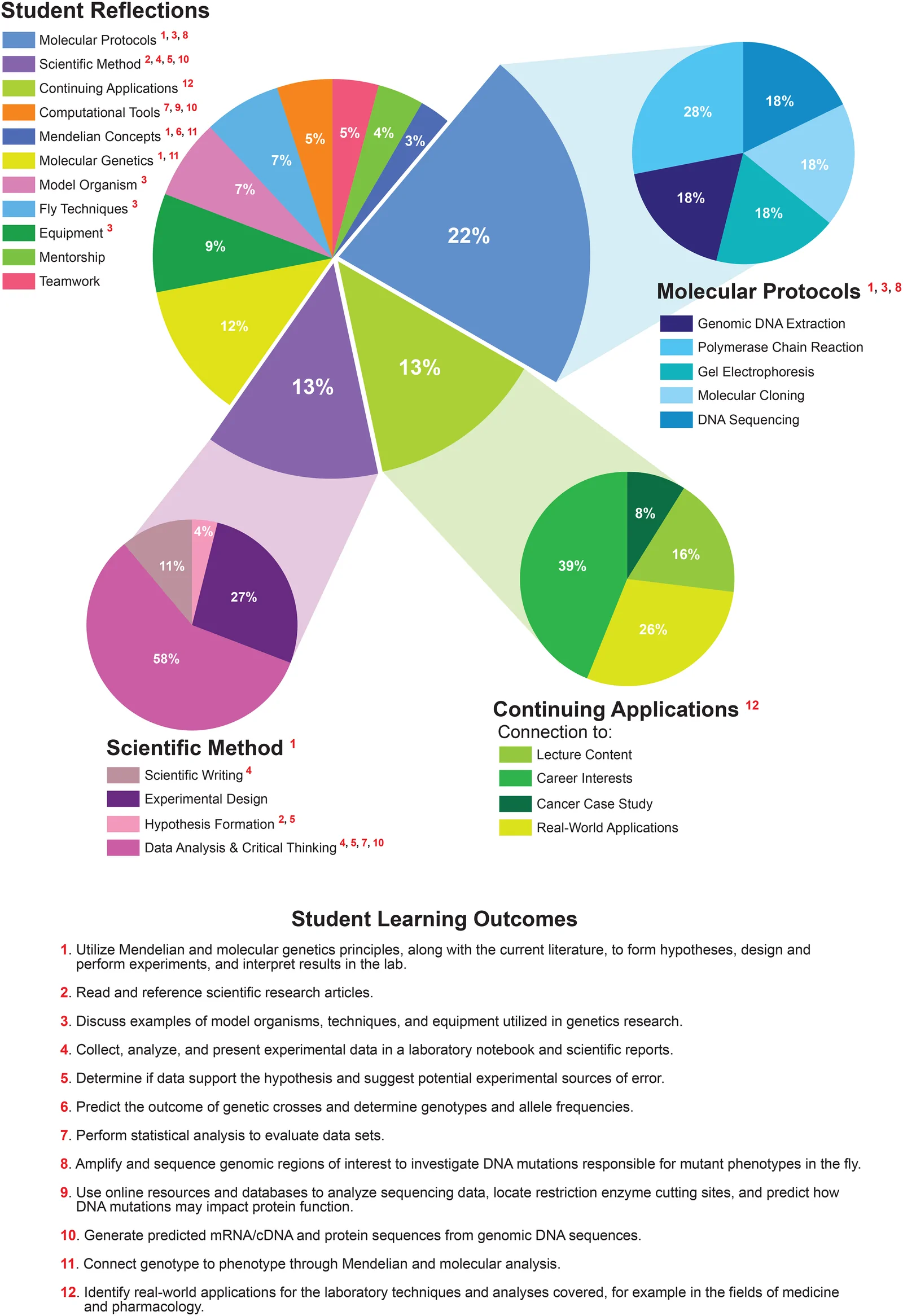
Student reflections – most valued genetics laboratory course concepts. Overall student course reflections highlighting what they valued in the course, with a more detailed look at their top three most valued concepts (Scientific Method, Continuing Applications, and Molecular Protocols), n = 296. The superscripts indicated for relevant topics connect to the student learning outcomes presented.

A course survey, as part of our program evaluation, was implemented to collect free responses from students for perspectives on the most valued laboratory course concepts, as shown in Fig 16. The highest valued components from the course, based on the survey analysis, were the hands-on molecular protocols (e.g., PCR, gel electrophoresis), scientific method (e.g., data analysis and critical thinking), and continuing applications (e.g., connection to their career interests and real-world applications). Student reflections were connected with the student learning outcomes as presented, indicated by the superscripts for relevant topics.

### Extracurricular student outcomes

Graduate and undergraduate research students were involved in the investigation of genes that are part of the curriculum described above, as shown in Fig 6. Students who previously engaged in the laboratory course, who shared their interest in research and education, have joined as extracurricular research students. This enabled them to provide valuable insight from their experiences in the course and contribute to investigation of new genes for integration into the course curriculum. Students engaged in oral and poster presentations at various conference localities (e.g., Undergraduate Research Colloquium and USF Health Research Day hosted by University of South Florida, as well as the Florida Undergraduate Research Conference) to showcase their efforts.

Furthermore, students had an opportunity to engage in manuscript preparation to publish their findings. However, authorship for such a large consortium is associated with some ethical issues, and we are cognizant of the publication challenges that may be associated with this [32]. The students may not merit authorship if they did not participate in the writing, but they may be referenced in acknowledgments where their valuable contributions can be acknowledged.

## Discussion

### Overview

In this paper, we have shared a model that could be adapted into an upper-level undergraduate Genetics Laboratory curriculum. This model focuses on the use of molecular biology techniques to uncover the connection between phenotype and genotype in *D. melanogaster*, with in-class laboratory and extra-curricular work incorporating inquiry-based and hypothesis-driven scientific inquiry, respectively. We discuss the findings for each gene and the extracurricular research opportunities this project provides for undergraduate and graduate students, who were involved in helping develop and optimize the curriculum and protocols for this course. A copy of the Fall, Spring, and Summer Lab Course Schedules are presented in S6 File. Curriculum updates have been implemented into our PCB3063L General Genetics Laboratory course at the University of South Florida over the past 26 semesters (~9 years), beginning in the summer 2017 term with the *sepia* gene project, followed by the *yellow* and *white* gene projects. With our future upcoming plan to integrate the *vestigial* gene into the curriculum, subsequent efforts include integrating the *apterous* and *scalloped* genes as future research projects.

A variety of learning models describing course-based undergraduate research experiences (CUREs) have shown to enhance student learning, many of which focus on small class sizes that offer research opportunities for undergraduate students [33]. Our proposed experiential learning curriculum has similar shared student gains, while also being unique from standard CUREs in that it can be applied and adapted to large-scale courses. This has value in being able to reach a large number of students while offering a similar introduction to research and collaborative learning environment.

Reproducibility and transparency have been a growing focus for the scientific community, with the US National Institutes of Health (NIH) outlining plans to enhance current processes in the biomedical research field [34]. Underscoring this importance begins at the classroom level, and the structure of our curriculum development design takes into consideration the reproducibility of student findings. The data generated by both the course students and extracurricular research students could be described as a consortium, which is a collaborative pool supporting the reproducibility of these findings. The robustness of the data generated by students is shown in replicates across the many sections and course semester iterations.

### *Sepia* gene findings

**Fig 17:**
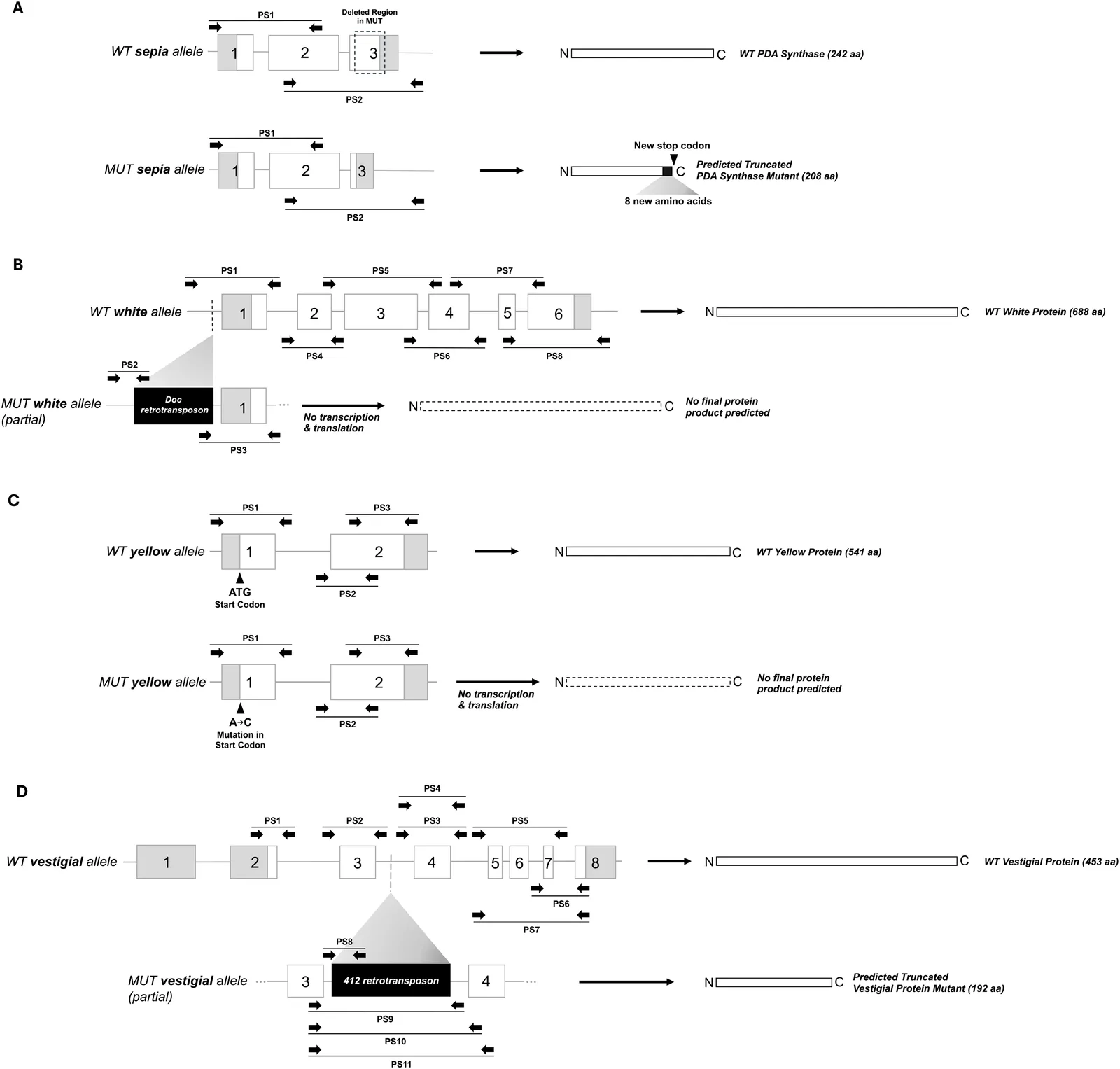
MUT Alleles and Predicted Translated Products. Database sequence from Ensembl Genome Browser was used to develop a map of the WT allele for each gene [31]. This figure contains components from Figure 18. **A.** WT *sepia* allele with the deleted region in MUT shown, with a full protein representation (PDA Synthase). MUT *sepia* allele is truncated in exon 3, with a predicted truncated PDA Synthase [6]. **B.** WT *white* allele showing the entire coding region and the full-length White protein is presented. A partial depiction of the MUT *white* allele showing the Doc retrotransposon insertion in the 5’UTR disrupting transcription/translation resulting in a predicted lack of White protein is shown [14–16]. **C.** WT *yellow* allele with the ATG start codon resulting in a full-length Yellow protein, and the MUT *yellow* allele showing the A to C point mutation disrupting transcription/translation resulting in a predicted lack of Yellow protein is shown [19–21]. **D.** WT *vestigial* allele showing the entire coding region and the full-length Vestigial protein is presented. A partial depiction of the MUT *vestigial* allele showing the 412 retrotransposon in intron 3 resulting in a predicted truncated Vestigial protein is shown [35].

We identified a novel *sepia* gene mutation, containing a 197 bp deletion (126 bp in exon 3 and 71 bp in the 3’UTR region of the *sepia* gene). Specifically, this deletion results in a loss of coding sequence in the *sepia* gene that encodes for 42 amino acids at the C-terminus of PDA synthase. In addition to this loss of coding sequence in exon 3, the deletion places sequence from the 3’ UTR region in frame, leading to the translation of 8 new amino acids at the C-terminus before reaching a stop codon as shown in Fig 17A. If the mutant *sepia* allele is successfully translated, it is expected to produce a truncated, non-functional protein [6]. PCR and sequencing results suggest the *sepia* mutant flies analyzed are homozygous recessive, with two copies of this mutant allele. Results are consistent with a loss-of-function mutation that disrupts PDA synthase expression and/or function, thus interfering with the drosopterin biosynthesis pathway to produce the observed mutant brown eye phenotype.

### *White* gene findings

Our mutant white-eyed flies contain a Doc retrotransposon insertion upstream of exon 1 as shown in Fig 17B, resembling a previously described *w1* allele [14–17]. This insertion likely results in a non-functional *white* allele by disrupting the promoter region that impacts gene transcription. Results are consistent with a homozygous recessive promoter region mutation responsible for the white eye phenotype. We determined the precise location of the insertion in the 5’UTR region of the *white* locus as shown in Fig 11. Exon 4 region of the *white* gene resulted in a larger than expected PCR amplified product for both mutant and WT samples. Further investigation into the exon 4 region is necessary to generate a complete cDNA sequence for the *white* gene to enable comparison with the database. However, preliminary analysis indicates that there is likely a disruption in this region of the *white* locus for both mutant and WT flies when compared to available database sequence. Phenotyping confirmed presence of eye-color pigments in WT flies; therefore, this suggests that the disruption observed in the exon 4 region may not impact White protein function, and further analysis is currently being conducted by research students.

### *Yellow* gene findings

The yellow body color mutant flies analyzed contain an A to C point mutation in the start codon of the *yellow* gene as shown in Fig 17C. This is consistent with a previously described *y1* mutant allele [36]. All WT flies analyzed in our population contained a functional ATG start codon. The findings are consistent with a homozygous recessive point mutation responsible for this yellow body color phenotype. We anticipate that this mutation (located at the start codon) disrupts transcription, likely resulting in a lack of Yellow protein production which is expected to disrupt the formation of black melanin in the cuticle of *D. melanogaster* [19–21].

### *Vestigial* gene findings

The mutant crumpled, vestigial winged flies analyzed contain a 412 retrotransposon insertion in intron 3 of the *vestigial* gene as shown in Fig 17D. This confirms a previously described *vg*^*BG*^ allele, and further work is needed to demonstrate that this mutation leads to the production of a truncated vestigial protein [24]. We predict this disruption to lead to the production of a truncated Vestigial protein mutant of 192 amino acids in length, presuming that the DNA sequence past exon 3 is not in frame or not translated due to the insertion. The findings are consistent with a homozygous recessive intronic region insertion mutation responsible for the vestigial wing phenotype.

### Coordinating research student work

A critical part of curriculum development and coordinating student research work involves clear and concise communication of each gene’s role and regulation (i.e., narrative) as well as optimizing the protocols to ensure their robustness for implementation into the lab coursework. Using one gene each semester as a framework for research student projects and ultimately for each semester’s molecular module curriculum allows for an adaptable model. The gene selected can be switched between semesters to offer variety in the coursework. The genes outlined in this paper (i.e., *sepia*, *white*, *yellow*, and *vestigial*), as well as other potential genes that are currently a work in progress (i.e., *apterous* and *scalloped*) offer undergraduate and graduate students an opportunity to work on each project from primer design to DNA sequencing and analysis. A potential follow-up course may be developed to expand on this project by investigating gene expression analysis or protein level analysis, to provide students with additional perspectives and approaches in which to understand foundational molecular biology concepts.

The time commitment for research coordination and curriculum development can vary for each part of the project. For example, the beginning and end of each gene narrative may require more involvement and mentorship for the research students. Providing a gene to investigate for each student and having a small group of 2–4 students to work in parallel can offer both a sense of independence and ownership in their project while simultaneously offering peer support. This beginning phase can involve 2–3 initial meetings, where students can familiarize themselves with the project, conduct literature review, and learn primer design or hands-on skills as needed. Testing primers and optimizing protocols can be completed based on the student’s schedules and typically require one lab meeting per week to address questions and provide guidance. Toward the end of the project, the cloning steps, troubleshooting, and DNA sequencing analysis can offer a valuable opportunity to teach research students detailed steps and molecular biology principles that may not always be possible within the usual time constraints in a lab course environment. Poster presentations have typically been produced by research students to be presented at research conferences and at lab meetings, which offer an opportunity to develop scientific writing, figure preparation, and presentation skills.

Typically, these projects have run based on the research student’s initiative and can have a broad scope based on their interest. With new pedagogical approaches placing emphasis on the value of STEAM which incorporates arts in STEM, graphic design offers an innovative avenue for students to connect with our research topics [37].These graphic design projects, such as those presented in Fig 1 through Fig 5 in this manuscript, gave our research students an opportunity to read current literature to understand what is known and unknown for each gene before putting together a scientifically accurate visual representation for our figures. Additionally, our lab manual cover pages have been prepared by research students that are interested in coupling art and science together as a part of their contribution to our curriculum development, an example of which is presented in S7 File.

### Curriculum outcomes

While academic “rigor” may have a range of definitions as perceived by students and teachers, it has been described as a hallmark of high-quality education [38,39]. From our perspective, scientific course rigor is meant to challenge students by providing an intellectually rich learning experience and help increase their self-efficacy by providing the support and guidance needed for students to be successful in their coursework. To better understand the student grade outcomes alongside the curriculum development integration where we prioritized maintaining a relevant and rigorous course, we examined student grade distributions from Fall 2023, Spring 2024, Summer 2024, and Fall 2024. This gave us insight that course rigor is not necessarily associated with student grade outcomes (e.g., where high grades may be associated with an easy course or vice versa). We hold that a course should provide both by appropriately challenging students to learn and apply course content at a high level while also providing them the tools and guidance necessary to be successful academically.

From student major distribution data gathered from Fall 2023 to Fall 2024, a variety of majors from diverse fields typically enroll in this course. While a large majority of students come from Biomedical Sciences, Biology, and Molecular Bioscience majors, students from an array of additional varied majors (e.g., Psychology, Health Sciences, and Marine Biology) are enrolled as well. This diversity in student majors reflects their background as well as their future career interests, which has been important in developing a course that provides valuable resources and learning experiences for all enrolled students. Furthermore, the undergraduate research student experience coupled with curriculum development has helped provide clarity to the level of academic background and understanding that students typically bring with them when entering this course. This has helped us provide appropriate level of support (via skill check activities or introduction of Peer Learning Assistants) needed to ensure the success of students in this lab course.

### Model organisms and budget considerations

We recommend *D. melanogaster* as the ideal model organism for this undergraduate Genetics Laboratory course curriculum, particularly to highlight the phenotype to genotype connections. As a cost-effective option, as well as to connect back to historical origins of Genetics, from Thomas Hunt Morgan’s initial investigation into the *white* gene of *Drosophila* to the evolution of modern Genetics throughout student’s introduction to Genetics lecture material, we believe this offers a unique learning opportunity for students in a Genetics Lab course [4].

In case of any budget or availability considerations, we suggest other potential model organisms as suitable for our proposed Molecular Biology experimental design (from phenotypic screening and gDNA extraction to DNA sequencing). If more readily available: (a) Mammalian cell culture – WT and mutant variants (YAC-1 lymphoma cells or similar) can potentially be obtained from a neighboring cancer research or mice facility, and the experimental design can be adapted to investigate cancer specific genes. This could offer an opportunity for more real-world connections to cancer case studies and allow students to follow important cancer biology pathways possibly relevant to their career interests; (b) Zebrafish embryos – If a zebrafish lab is available nearby, WT and MUT samples can be obtained. The experimental design can be adapted to target developmental biology and specific genes to investigate; and (c) Bacteria (*E. coli* or similar) – if easier to obtain, this can be a suitable option to investigate phenotypes that impact environmental persistence or resistance to antibiotics. While these may need to be modified to make safe for teaching laboratory use, they could provide a more applicable option (e.g., if gathered from nearby water sources to test for virulence), and be a more cost-effective alternative compared to the above options [40].

### Potential adaptations and companion courses

Thus far, we have focused on the curriculum design for the General Genetics Laboratory course at our institution. Primarily, this course curriculum covers DNA level analysis by offering students a specific project that allows students to follow their experiment from phenotypic screening and DNA extraction to DNA sequencing and analysis, ultimately to understand how DNA mutations may impact protein function. This course design can be adapted for lower-level introductory Biology laboratories as well as upper-level Molecular Biology laboratories, depending on the institution and course structure.

In addition to this DNA level analysis, we suggest potential companion courses that can perform investigations at the RNA and protein level. These courses could be taken concurrently or sequentially, to build upon the techniques and concepts learned in this course. For example, at our institution, three different courses are designed to cover DNA level analysis, RNA level analysis (including RNA extraction, primer design, gene expression analysis), and protein level analysis (including protein isolation, quantification, western blot, and functional analysis). Each of these courses is designed around an overarching experimental design that is followed by students throughout the course of one semester. For advanced courses, potential adaptations can focus on clinical relevance or perhaps molecularly mapping uncharacterized mutant lines and designing CRISPR/Cas9-generated alleles.

## Materials and methods

### Ethics statement

This paper presents work solely on Genetics curricular materials and the results of the program evaluation. Prior to conducting the anonymized survey for the end of course evaluation, our protocol was submitted to the University of South Florida Institutional Review Board (USF IRB), and a determination of Not Human Subjects Research was made.

### *D. melanogaster* information

Flies were obtained from Carolina Biological (WT-172100, Sepia-172575, White-172220, Yellow-172270, Vestigial-172460, Apterous-172320) and maintained at 25°C. FlyNap (Carolina, 173025) was utilized to anesthetize the flies prior to initiating the screening process to verify appropriate phenotypes for genomic DNA extraction.

### Genomic DNA extraction

In a 1.5 mL tube, 200 μL of lysis buffer (0.1% SDS, 10 mM Tris pH 8.0, 1 mM EDTA pH 8.0) was added to 10 flies of each phenotype (WT, s*epia*, w*hite*, y*ellow*, v*estigial* and a*pterous*), gently crushed with a pestle for 5 minutes and then centrifuged (ThermoScientific, 75002436) at 13,000 rpm for 1 minute. The supernatant was transferred to a fresh 1.5 mL tube and 200 μL of phenol/CHCl_3_/Isoamyl 25:24:1 (ThermoFisher, BP1752I-400) was added. This was then vortexed for 30 seconds followed by centrifugation for 5 minutes at 13,000 rpm. For each sample, 100 μL of the top aqueous layer was collected into a fresh 1.5 mL tube and 4 μL of 10 mg/mL RNase A (ThermoFisher, FEREN0531) was added, vortexed briefly, and incubated at room temperature (RT) for 30 minutes. Gel loading dye (10X stock concentration) was added to 5 μL of each RNase-treated gDNA sample and they were run on 1% agarose gels in 1X TBE (Tris-borate-EDTA) Buffer along with Tridye 100 bp ladder (NEB) at 110–125 volts and visualized with ethidium bromide under ultraviolet (UV) light.

### Primer design

**Fig 18:**
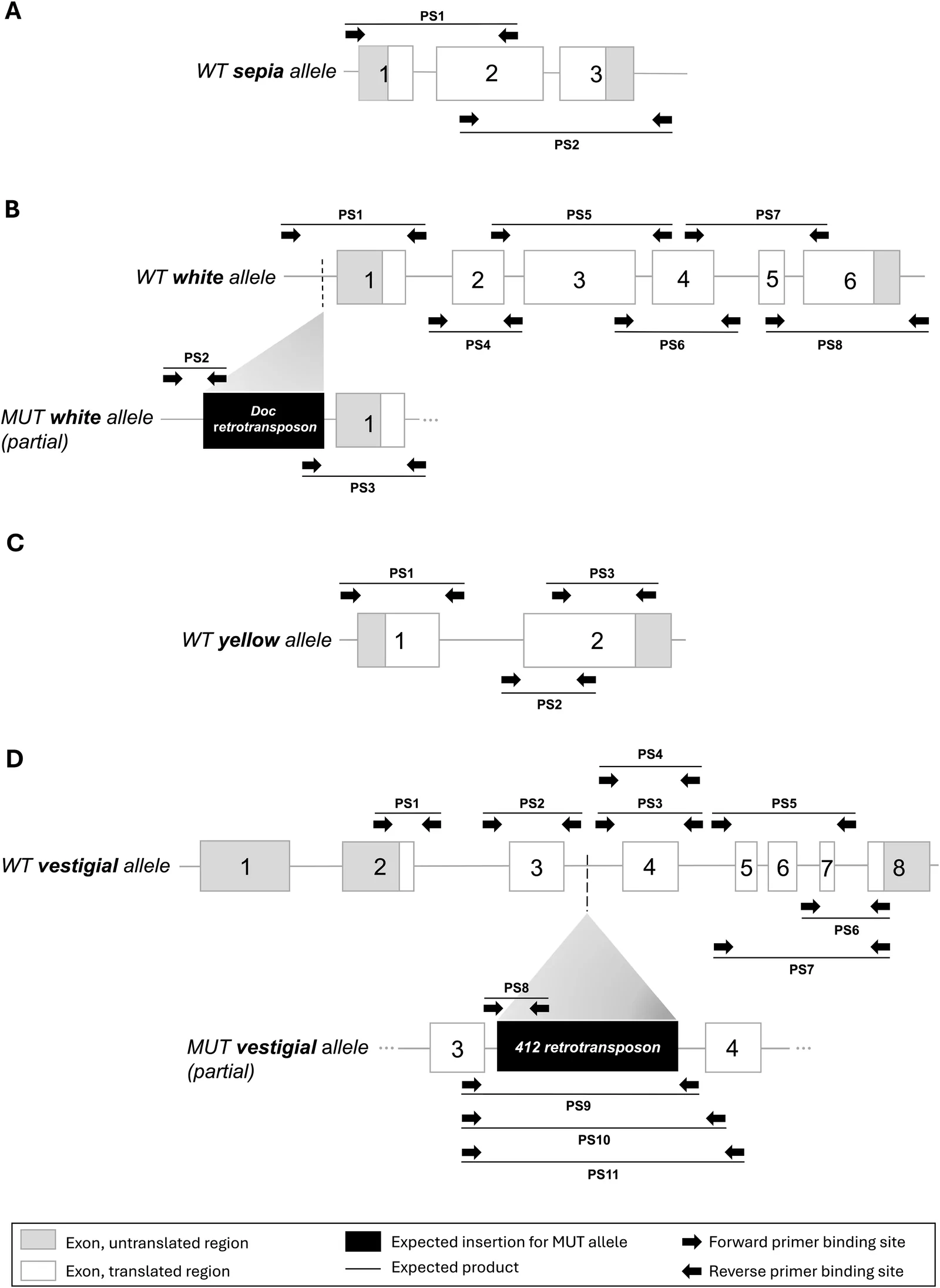
Allele representations for *Sepia*, *White*, *Yellow*, and *Vestigial* genes. A map of the WT allele for each gene was designed by referencing the database sequence from Ensembl Genome Browser [31]. This figure is expanded upon in Fig 17. **A.** The WT *sepia* allele shows two primer sets (PS1 and PS2) covering the coding region of the *sepia* gene. **B.** The WT *white* allele shows six primer sets (PS1, PS4-PS8) covering the coding region of the *white* gene. The MUT *white* allele shows PS2 and PS3 covering the 5’ and 3’ insertion junction sites of the Doc retrotransposon [14–16]. **C.** The WT *yellow* allele shows three primer sets (PS1-PS3) covering the coding region of the *yellow* gene. **D.** The WT *vestigial* allele shows seven primer sets (PS1-PS7) cover the coding region of the *vestigial* gene. PS8-PS11 cover the insertion 412 retrotransposon insertion in the *vestigial* mutant allele [35].

Primers were designed to cover selected regions of interest for each gene (i.e., *sepia*, *white*, *yellow*, and *vestigial*) using database sequences obtained from Ensembl genome browser [31]. As shown in Fig 18 and Table 1, multiple primer sets are utilized to amplify genomic DNA in manageable sizes for cloning and Sanger sequencing analysis.

### Polymerase chain reaction (PCR) amplification

For each PCR reaction, 2 μL dilutions (1:20) of each gDNA sample (for WT and *sepia*, *white*, *yellow*, or *vestigial* gene mutant flies) were added to 4 μL of Taq 5X Mix (New England Biolabs), 12 μL molecular grade water, and 1 μL each of forward and reverse 10 working stocks of gene-specific primers obtained from IDT (Table 1). For a negative control, 2 μL of molecular grade water was used in place of gDNA. These samples were run with optimized annealing temperatures and extension times (Table 2) for each gene. Gel loading dye (10X stock concentration) was added and the samples were run on 1% agarose gels in 1X TBE Buffer along with Tridye 100 bp ladder (NEB) at 110–125 volts and visualized with ethidium bromide under UV light.

**Table 2:** PCR thermal cycling details.

	*sepia*	*white*	*yellow*	*vestigial*
Initial denaturation, 1 min	95°C	95°C	95°C	95°C
Denaturation, 20 seconds	95°C	95°C	95°C	95°C
Annealing, 20 seconds, 1 min	59°C	55°C	55°C	58°C
Extension, 4 mins	90 seconds	90 seconds	120 seconds	90 seconds
Go to step 1, total cycles:	38 total cycles	38 total cycles	38 total cycles	38 total cycles
4°C	hold	hold	hold	hold

### Topoisomerase-mediated ligation, transformation, and cloning

PCR amplified products or PCR grade water for the no insert control (up to 4 μL) were added to Salt Buffer and pCR4-TOPO Vector (Invitrogen) followed by incubation at RT for 10 minutes. This reaction (4 μL) was added to ice-thawed One Shot TOP10 Chemically Competent *E. Coli* cells (Invitrogen) followed by gentle tapping and incubation on ice for 10 minutes. The cells were heat shocked for 30 seconds at 42°C and returned to ice briefly for recovery. Warm SOC (super optimal broth with catabolic repressor) medium (200 μL) was next added to each transformed sample and placed in a shaker for 40 minutes at 37°C. Samples (up to 150 μL) were spread on Lysogeny Broth/Ampicillin (LB/Amp) plates, and incubated overnight at 37°C. A single colony from each clone LB/Amp plate was selected for inoculation into a 2 mL LB/Amp liquid culture and then placed in the shaker set at 250 rpm for 18 hrs at 37°C.

### Plasmid DNA extraction using Qiagen Spin Miniprep Kits

For each sample (WT, *sepia*, *white*, *yellow*, and *vestigial*), liquid culture (1.5 mL) was centrifuged at 8,000 rpm for 1 minute, and the supernatant was discarded. The pelleted bacterial cells were resuspended in 250 μL Buffer P1 with RNase and vortexed until no cell clumps remained. Then Buffer P3 (250 μL) was added and samples were inverted gently six times to mix. Within 5 minutes of lysis, Buffer N3 (350 μL) was added and inverted gently six times to avoid localized precipitation. Each sample was centrifuged at 13,000 rpm for 10 minutes and the supernatant was collected to add to spin columns with a collection tube. This was next centrifuged for 1 minute at 13,000 rpm. After the flow through was discarded, Buffer PE (750 μL) was added and samples were centrifuged again for 1 minute at 13,000 rpm as a wash step. After discarding the flow through, each sample was centrifuged again for 1 minute at full speed to remove residual liquid. To elute the plasmid DNA, nuclease-free water (50 μL) was added to the center of each column, followed by a 1 minute incubation and centrifugation for 1 minute at 13,000 rpm. The isolated plasmid DNA was quantified by NanoDrop UV Spectrophotometer (Fisher Scientific).

### Restriction enzyme digestion of plasmid DNA

**Fig 19:**
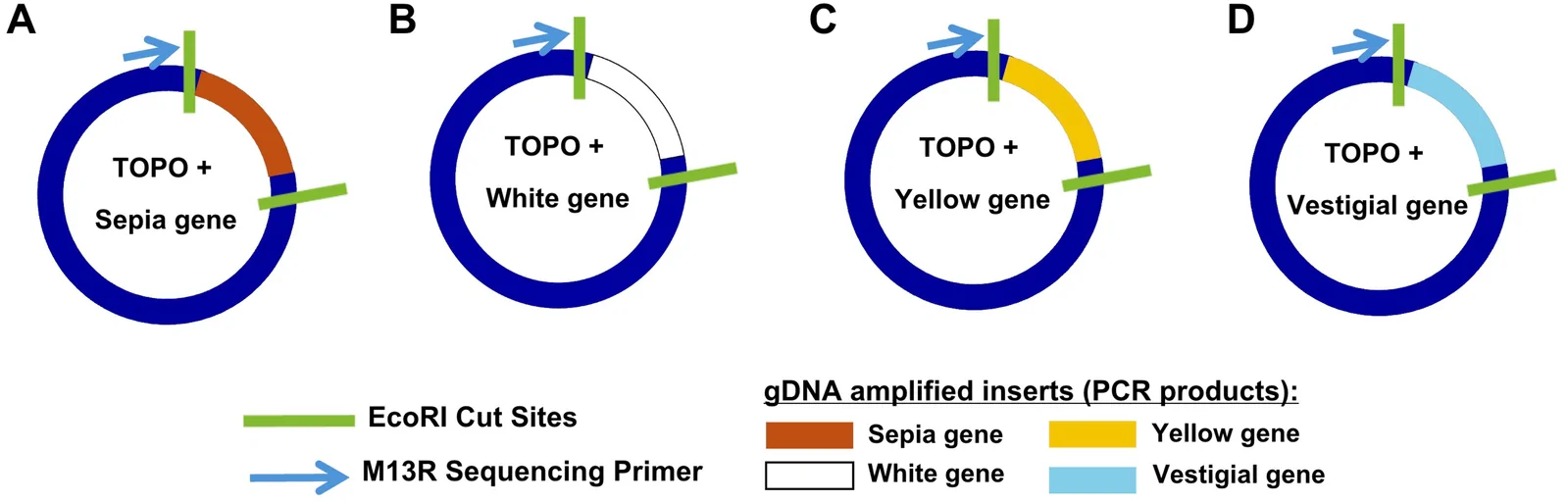
Restriction digest representation. PCR amplified regions for each gene (inserts) were ligated into the TOPO vector to generate the plasmids shown. Following transformation and miniprep, isolated plasmid DNA was digested with EcoRI to verify presence of inserts.

For each sample (WT, *sepia*, *white*, *yellow*, and *vestigial*), isolated plasmid DNA (0.2-1 μg) was digested with EcoRI enzyme (1 μL, New England Biolabs) with 10X EcoRI Reaction Buffer (2 μL) for 50 minutes at 37°C. Digested and undigested control samples were run on 1.5% agarose gels in 1X TBE Buffer, along with 1 kb and 100 bp Tridye ladders (ThermoFisher) at 110–125 volts. Bands were then visualized with ethidium bromide under UV light. NEB Cutter (https://nc3.neb.com/NEBcutter) was used to check for EcoRI cut sites within the PCR inserts. A visual representation of the plasmids (PCR fragments ligated into TOPO vector) with the EcoRI restriction sites is presented in Fig 19.

### DNA sequencing

The binding location for the primer used (M13R) for the sequencing methods described herein is presented for reference as a schematic in Fig 19. For shipping out to Eurofins, 10 μL of plasmid DNA for each sample was sent to Eurofins (SimpleSeq, stored at −20°C prior to shipping), after verifying the presence of insert by restriction enzyme digestion, validation of sample purity (~1.8 A260/280 value), and determining concentration (40–150 ng/ μL).

For in-house sequencing of each sample, an ABI3130 Sequencer (Applied Biosystems) was utilized after running a sequencing reaction (Thermofisher, BigDye Terminator Kit) in the thermal cycler (program settings are summarized in Table 3) for each sample. The sequencing reaction was as follows: 150 ng-300 ng plasmid DNA (10 μL), 1.6 μM M13R primer stock (~10 ng/ μL, 2 μL), Reaction Master Mix (2 μL), 5x Buffer (3 μL), molecular grade water (3 μL).

**Table 3:** Thermal cycling program for cycle sequencing reaction.

Initial denaturation, 1 min	96°C
Denaturation, 20 seconds	96°C
Annealing, 20 seconds, 1 min	50°C
Extension, 4 mins	60°C
Go to step 1, total cycles:	29 total cycles
4°C	hold

### Computational sequence analysis

ApE (A plasmid editor, https://jorgensen.biology.utah.edu/wayned/ape) was used to view, trim, and annotate raw sequence and chromatogram files. Raw sequences were analyzed using NCBI (National Center for Biotechnology) Nucleotide BLAST (Basic Local Alignment Search Tool) using the following settings: (a) RefSeq Reference Genomes; organism; (b) organism: *Drosophila melanogaster*; and (c) optimize for: megablast; https://blast.ncbi.nlm.nih.gov/Blast.cgi). The following steps were applied to generate a compiled cDNA sequence file for WT and MUT sequences for the *sepia* and *yellow* genes which are presented in S3 and S4 Files: (a) sequences in reverse orientation were reverse complemented, (b) sequence from overlapping regions of primer sets was removed to represent combined consensus sequence, (c) 5’UTR was removed, (d) intronic sequences and 3’UTR were removed where appropriate, and (e) consensus sequences were generated from data obtained by following highest quality chromatogram reads across greater than 50 replicate sequence data (for fall or spring semesters) per fragment of interest. The compiled cDNA sequences were compared to database sequences by using NCBI nucleotide BLAST, and the ExPASy (Expert Protein Analysis System) translate tool (https://web.expasy.org/translate) followed by protein BLAST. T-Coffee (Tree-based Consistency Objective Function for Alignment Evaluation) Multiple Sequence Alignment Server (https://tcoffee.crg.eu/apps/tcoffee/do:regular) was used to compare all three sequences (database, WT, and MUT).

### Glycerol stocks of clones

Sequence verified plasmid DNA samples were inoculated into liquid culture (LB/Amp) and incubated in a shaker overnight at 37°C. Glycerol stocks were prepared by adding 500 μL of 50% glycerol to 500 μL of the overnight culture and stored at −80°C.

### Figure preparation

Figures were prepared using Microsoft PowerPoint, Procreate, and Adobe Illustrator. Raw gel images did not undergo any manipulation beyond cropping excess gel (e.g., unused lanes) and minimally increasing contrast to visualize all bands present.

### Student learning outcomes and assessments

Student learning outcomes for the General Genetics Laboratory course, which are presented in S8 File focus on foundational molecular genetics concepts, scientific inquiry from literature search and hypothesis design to interpreting results, as well as hands-on lab techniques and protocols. Designed to follow an experiment throughout the entire semester, rather than isolated labs each week on independent topics, this offers a more involved experience for students where they can follow their data and build on each week’s content to form a bigger picture. This also allows students to obtain a practical and representational idea of a scientific research timeline in terms of experimental design. Troubleshooting steps for PCR and preparing additional samples to account for potential low yield or purity in DNA extraction or plasmid DNA isolation have been integrated to account for situations where expected results are not attained. Students complete a total of 3 lab reports, a partial lab report at the end of the Mendelian module, a rough draft submitted for feedback partway through the Molecular module, and the final full lab report for the Molecular module at the end of the semester. A schematic representing the curriculum is provided in Fig 5, and further details of the course schedule, design, and assessments are provided in S6 and S8 Files. Students are assessed on their understanding of the underlying concepts rather than successful completion of procedures, and emphasis is placed on analysis and data interpretation.

### Curriculum design

Instructors are welcome to reach out to us for any additional course related components and materials.

With respect to the inquiry-based aspect of the course, students are encouraged to review existing literature and form three drafts of their hypothesis statement. They are provided feedback in class and on their lab report rough draft as they continue to generate their working hypothesis based on information they have gathered and the specific details for each lab section. This process offers the instructors to check student understanding and correct misconceptions, while giving students an opportunity to think carefully about the experimental design. We expect the hypothesis to change slightly across course iterations, between sections and student groups, as students collectively work toward uncovering the DNA mutations responsible for the phenotypes under investigation for their population of flies. Instructors facilitate hypothesis formation by ensuring that students are addressing the research question, that the hypothesis is testable, clear, concise, has sufficient rationale, that students have access to fundamental information that is known about the project, and that they know what remains unknown.

In each lab section, the laboratory layout consists of 24 students with 6 groups of 4 students each, with one gene narrative under investigation for a given semester. When implementing a gene narrative into the coursework, larger genes that have many primer sets to span the coding region and regions of insertion pose a time challenge for how many primer sets can be examined by the students through their lab course work (spanning only one semester). For this reason, focusing on having a maximum of 2–3 primer sets of interest integrated into the coursework per semester can be helpful. When following one gene narrative, the focus is on allowing a student group to follow one of two or three primer sets for that semester, and with 6 groups in a lab section this can allow for at least one replicate in another group so they can gather the necessary data for the whole class to analyze. For example, the whole coding region can be covered for smaller gene narratives like *sepia* and *yellow*. The *sepia* gene’s two primer sets are split so 3 groups are following a primer set each, and the *yellow* gene’s three primer sets are split so 2 groups are following a primer set each. For the larger genes like *white* and *vestigial*, we focused on following the regions of insertion for the class to have a total of 2 or 3 primer sets to follow in a similar vein. Specifically for the *white* gene, primer sets 1–3 have been incorporated into the classes, and we plan to incorporate primer sets 8 and 10 for the *vestigial* gene. The primer design component of research student work is indicated by an (*) in Fig 5, detailed in Table 1, and further details are provided in S9, S10, and S11 Files.

Due to the nature of once-a-week lab meetings and the variability in successful ligation into TOPO and selecting positive colony transformants, along with potential lab space limitations where the necessary space in the shaker may not be available for hundreds of student samples, inoculating bacterial cultures for all student plated samples poses restrictions. To address this, and to introduce some standardization to account for instances where the colony picked may be an empty vector, glycerol stocks of clones that have been sequence verified by research students are saved for each gene and used by the Genetics Prep TA to inoculate liquid bacterial cultures. Further, preparing glycerol stocks of each verified clone allows for us to monitor for culture density (concentration of bacterial cells) based on culture turbidity and spectrophotometer readings for culture density, which can be hard to predict on a large scale for individual samples. This step is critical in determining the concentration of plasmid DNA recovered by students in the plasmid extraction procedure needed to support the restriction digestion and sequencing segments. This step is outlined as the ‘amplification’ component of research student work indicated with an (*) as shown in Fig 5. Making glycerol stocks of clones is especially helpful for shorter summer lab schedules, which run similarly to shorter semesters due to any weather-related events (i.e., hurricanes, tropical storms, winter storms), that may not fully complete the cloning and transformation procedures, to enable progression with plasmid extraction procedure (i.e., mini prep). In addition to this, there are typically fewer sections in the summer semesters, which makes it an ideal time to test and begin integration of a new gene narrative into the curriculum.

### Program evaluation – course feedback design and analysis

A program evaluation that incorporated an anonymous end of the semester survey was conducted to obtain student reflections and feedback pertaining to the molecular module of the laboratory course. The two questions posed were open-ended and designed to elicit open, thoughtful responses from students based on their experiences throughout the course, see S12 File. Major themes in the course design (i.e., student learning outcomes, major techniques) and any other frequently noted themes identified in the student answers were tagged and analyzed, with details provided in S13 File.

## Supporting information

S1 File Original gel images. Raw original gel images are presented.
(PDF)

S2 File Quantification data. Concentration (ng/ μL) and purity (A260/280) values for our most recent *sepia*, *white*, and *vestigial* plasmid DNA samples prior to sequencing is presented.
(XLSX)

S3 File Compiled *Sepia* cDNA sequences. Primer set 1 (PS1) and Primer set 2 (PS2) data is shown, which has been combined for WT and *sepia* MUT samples to generate a cDNA sequence that can be translated for further analysis.
(PDF)

S4 File Compiled *Yellow* cDNA sequences. Primer set 1 (PS1), Primer set 2 (PS2), and Primer set 3 (PS3) data is shown, which has been combined for WT and *yellow* MUT samples to generate a cDNA sequence that can be translated for further analysis.
(PDF)

S5 File Lab report guidelines and rubric. The general lab report guidelines, along with lab report 2 guidelines and rubric pertaining to the Molecular component of the curriculum are presented.
(PDF)

S6 File PCB3063L fall, spring, and summer laboratory course schedules. The laboratory course schedules with the experiment details for each week are presented. Fall and Spring semesters are typically longer semesters. To account for the shorter time allotted for summer semesters, ligation/transformation procedures are limited to the concepts being covered in a brief lecture after the midterm exam.
(PDF)

S7 File Lab manual cover page – research student artwork. Cover Art by Parker Jain, as a part of the undergraduate research experience, is shown.
(PDF)

S8 File PCB3063L genetics laboratory fall syllabus copy. Student learning outcomes, general course description, class format, and detail on course components are detailed.
(PDF)

S9 File Annotated *Sepia* gene sequence. *D. melanogaster sepia* gene (*se* FBgn0086348, location: 3L:8,520,552−8,521,489) annotated with primer binding sites underlined in the database sequence taken from Ensembl genome browser is presented.
(PDF)

S10 File Annotated *Yellow* gene sequence. *D. melanogaster yellow* gene (*y* FBgn0004034, location: Chromosome X: 356,509−361,245) annotated with primer binding sites underlined in the database sequence taken from Ensembl genome browser is presented.
(PDF)

S11 File Annotated *White* gene sequence. *D. melanogaster white* gene (*w* FBgn0003996, location: Chromosome X: 2,790,599−2,796,466) annotated with primer binding sites underlined in the database sequence taken from Ensembl genome browser is presented.
(PDF)

S12 File PCB3063L fall 2024 student survey copy. As a means of evaluating the program, an anonymous end of the semester survey was conducted to obtain student reflections on the course.
(PDF)

S13 File Visual representation of tags assigned for course feedback analysis. A figure depicting tags and the breakdown for each category served as a rubric to maintain consistency across the student responses analyzed is shown.
(PNG)

## Abbreviations

ABC transporter protein: ATP-binding cassette transporter protein
Amp: Ampicillin
cDNA: Complementary DNA
ExPASy: Expert Protein Analysis System
LB: Lysogeny Broth
LIM-HD protein family: named after the first three gene in which this type of homeodomain was identified: Lin-11, Islet-1, and Mec 3
MUT: Mutant
MWM: Molecular weight marker
NCBI BLAST: National Center for Biotechnology Information Basic Local Alignment Search Tool
PCR: Polymerase Chain Reaction
PDA synthase: Pyrimidodiazepine synthase
pDNA: Plasmid DNA
RNase: Ribonuclease
SOC: Super optimal broth with catabolic repressor
TBE: Tris-borate-EDTA
T-Coffee: Tree-based Consistency Objective Function for Alignment Evaluation
UTR: untranslated region
UV: Ultraviolet light
WT: Wild-type
