# Supplementary material for: Identifying DNA mutations in Sepia, White, Yellow, and Vestigial genes in D. melanogaster - Integrating inquiry-based experiential learning into undergraduate Genetics Laboratory curriculum
Source: PLoS One. 2026 Sep 1;21(9):e0356998. doi: 10.1371/journal.pone.0356998 (PMC13533379; doi:10.1371/journal.pone.0356998)

Original Gel Images

Figure 7. Isolation of Genomic DNA. A

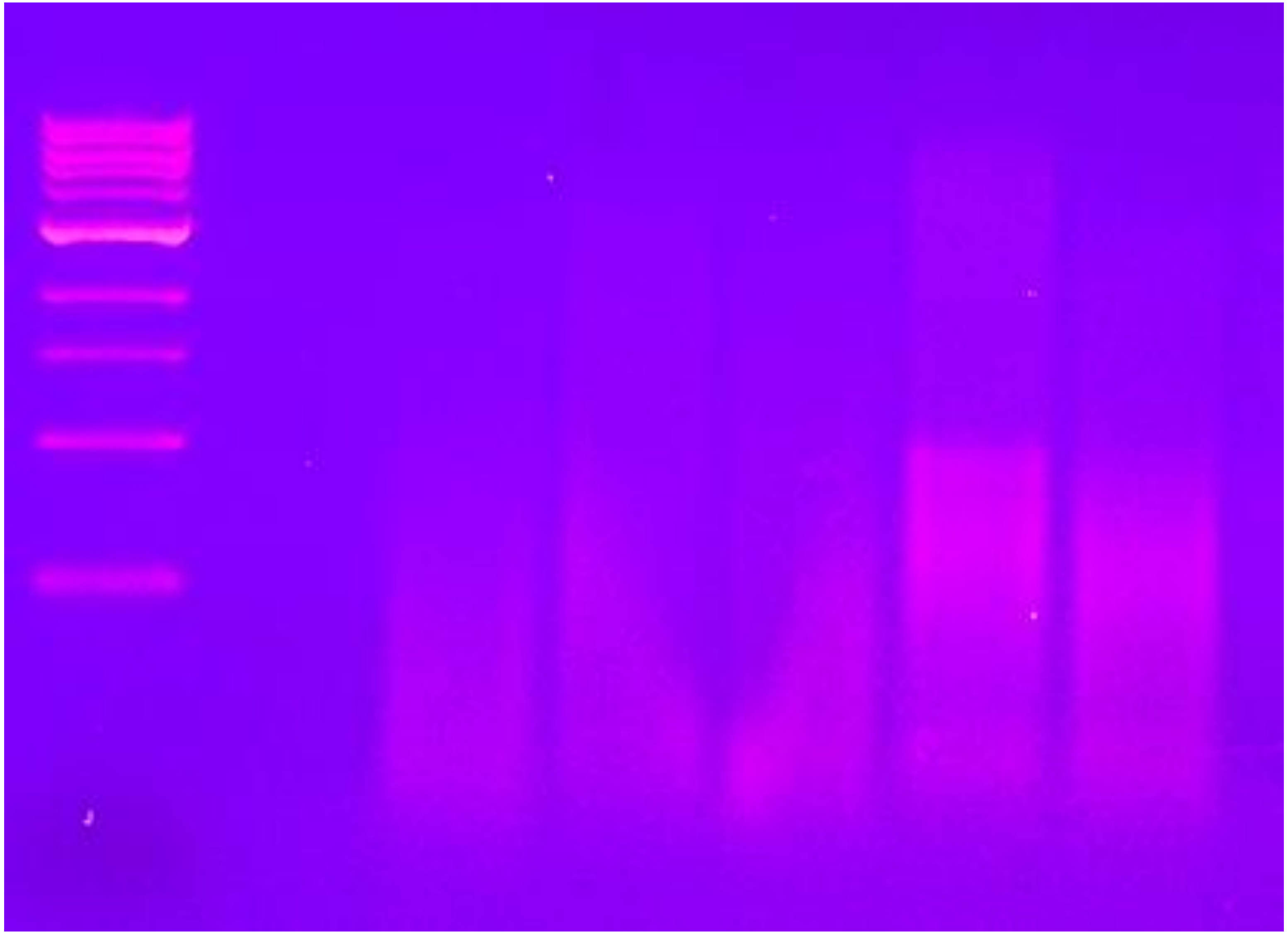

Figure 7. Isolation of Genomic DNA. B

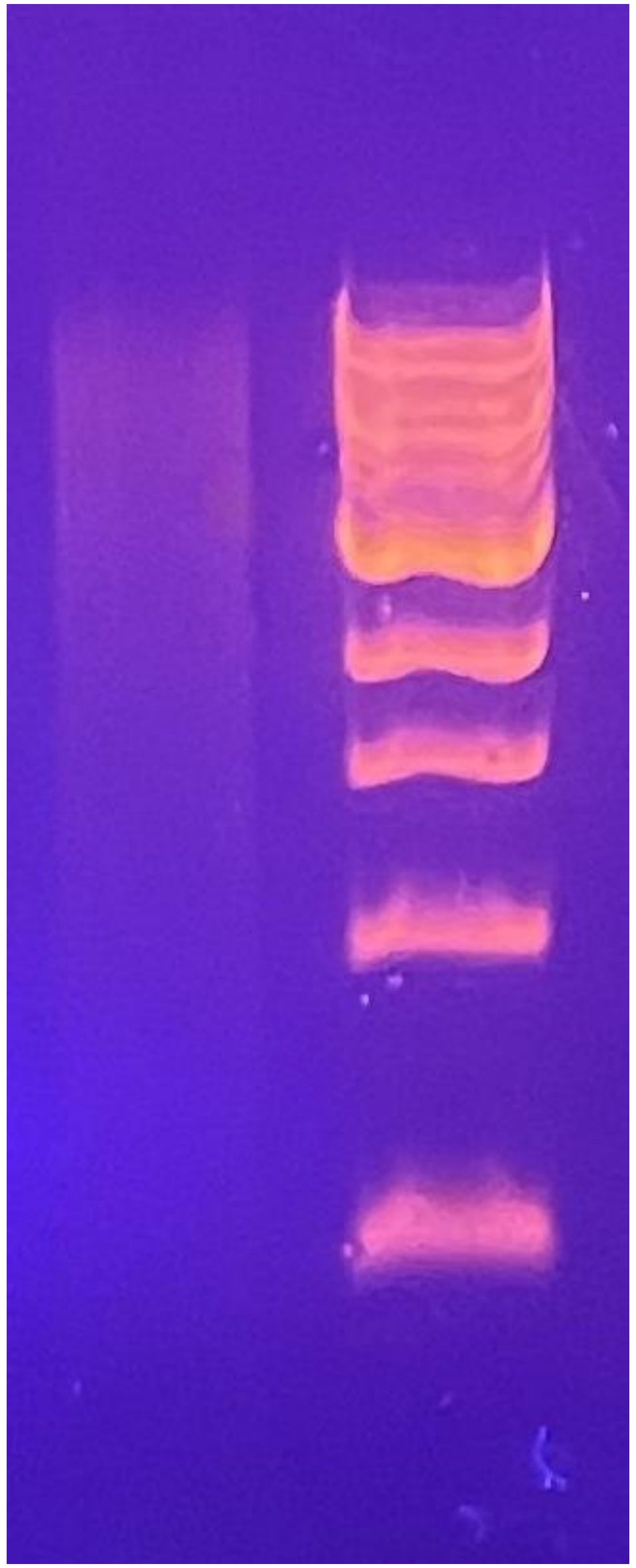

Figure 8. Sepia Gene Coding and Digest Gel. A.

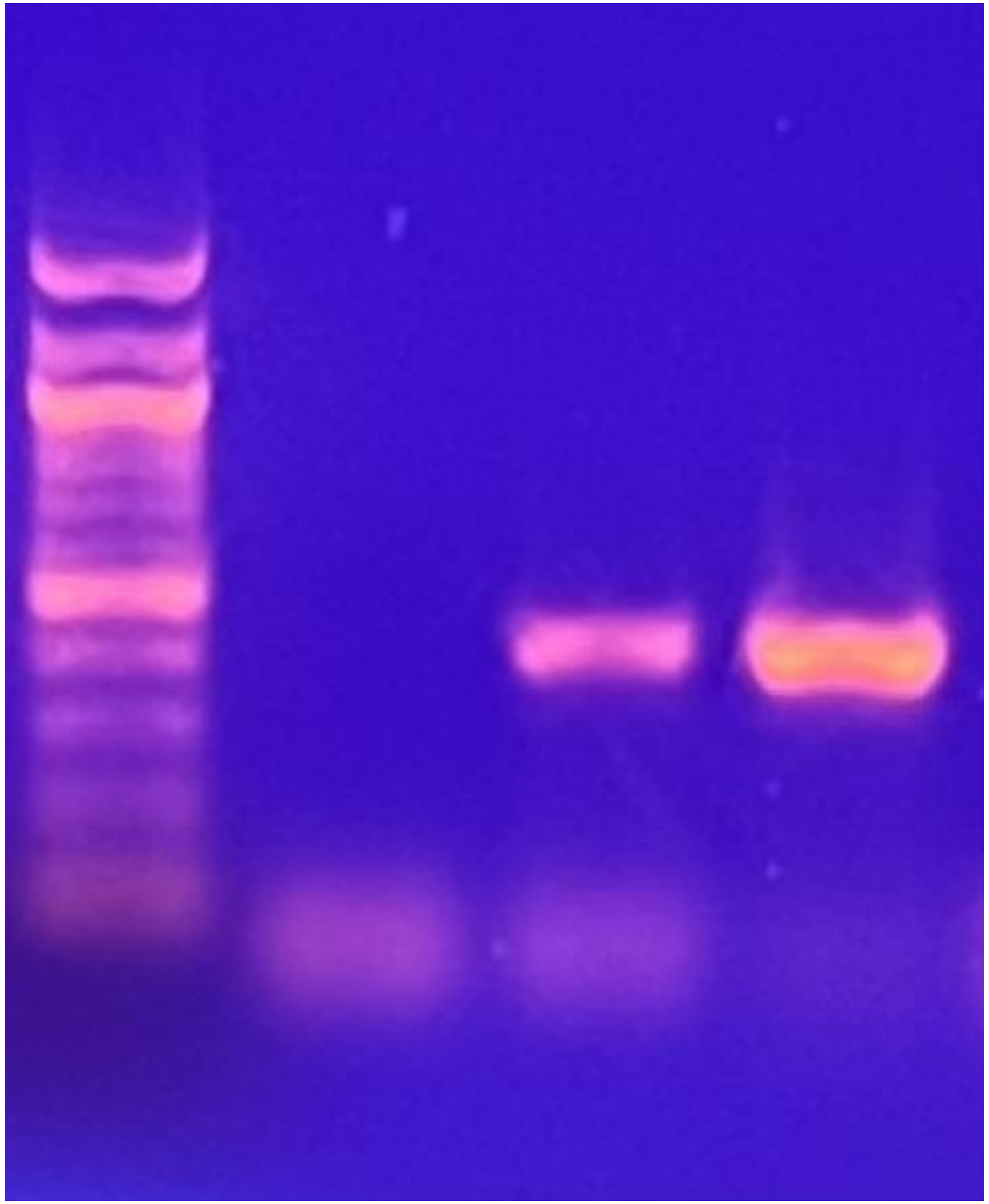

Figure 8. Sepia Gene Coding and Digest Gel. A.

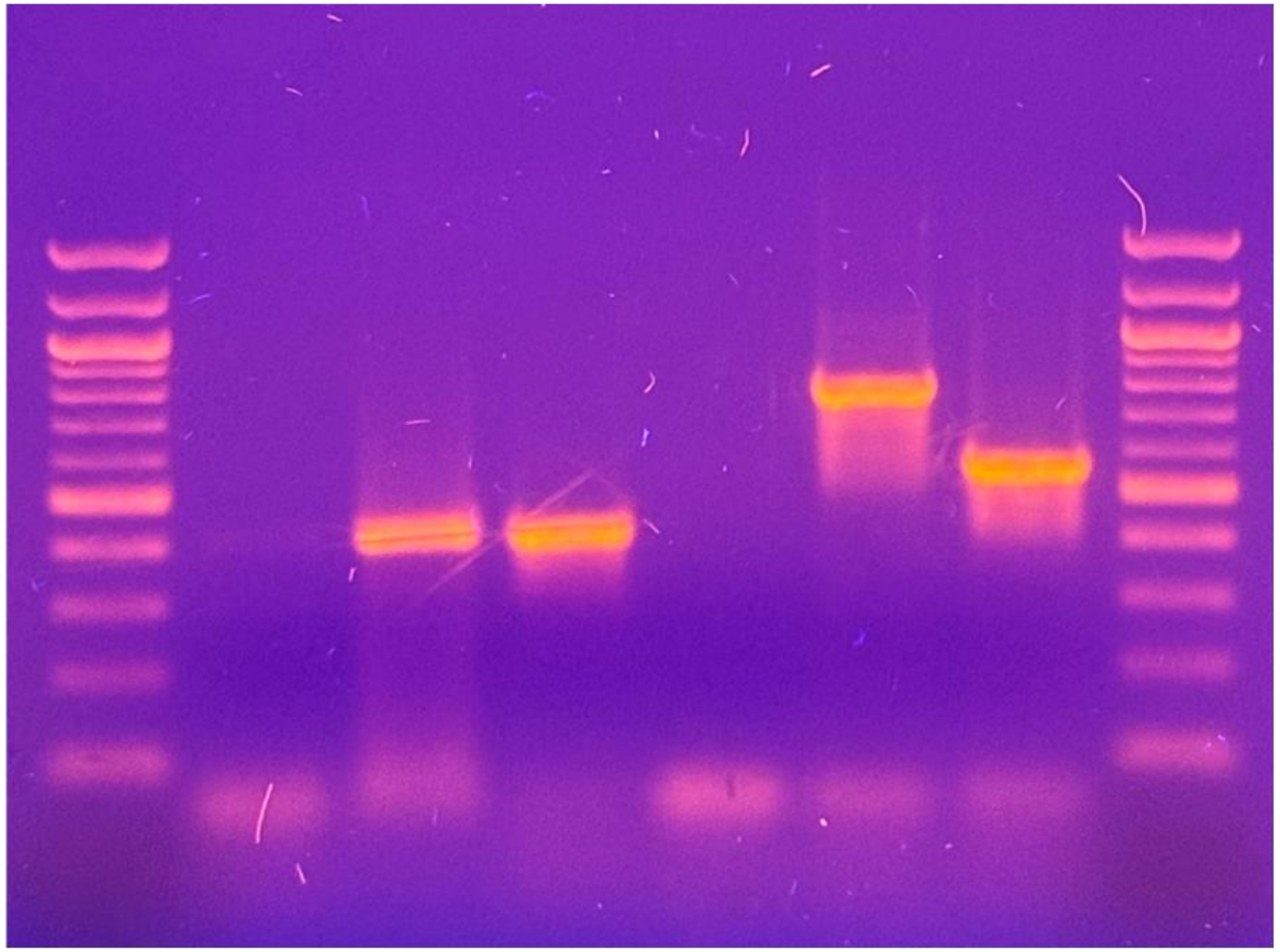

Figure 8. Sepia Gene Coding and Digest Gel. B.

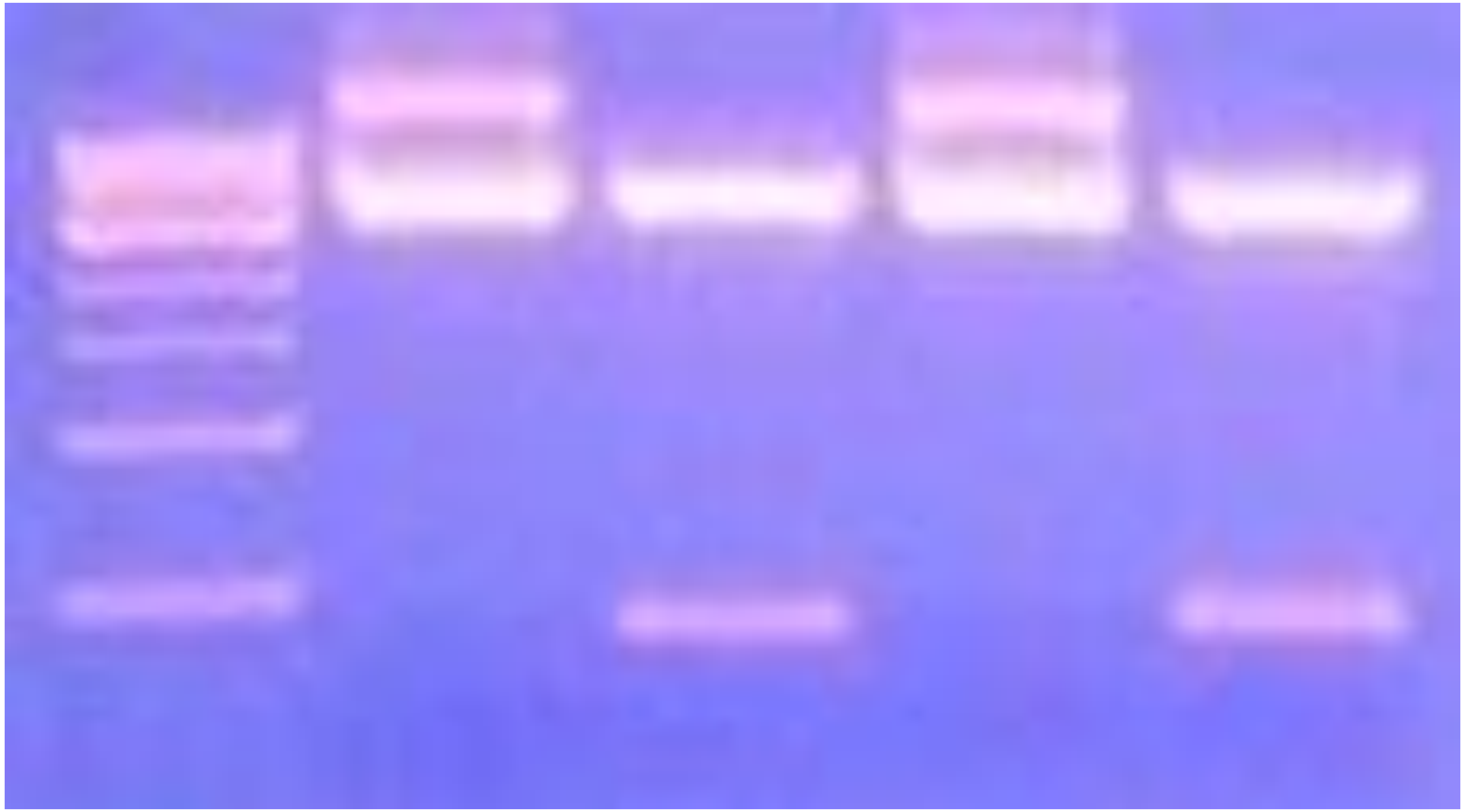

Figure 8. Sepia Gene Coding and Digest Gel. B.

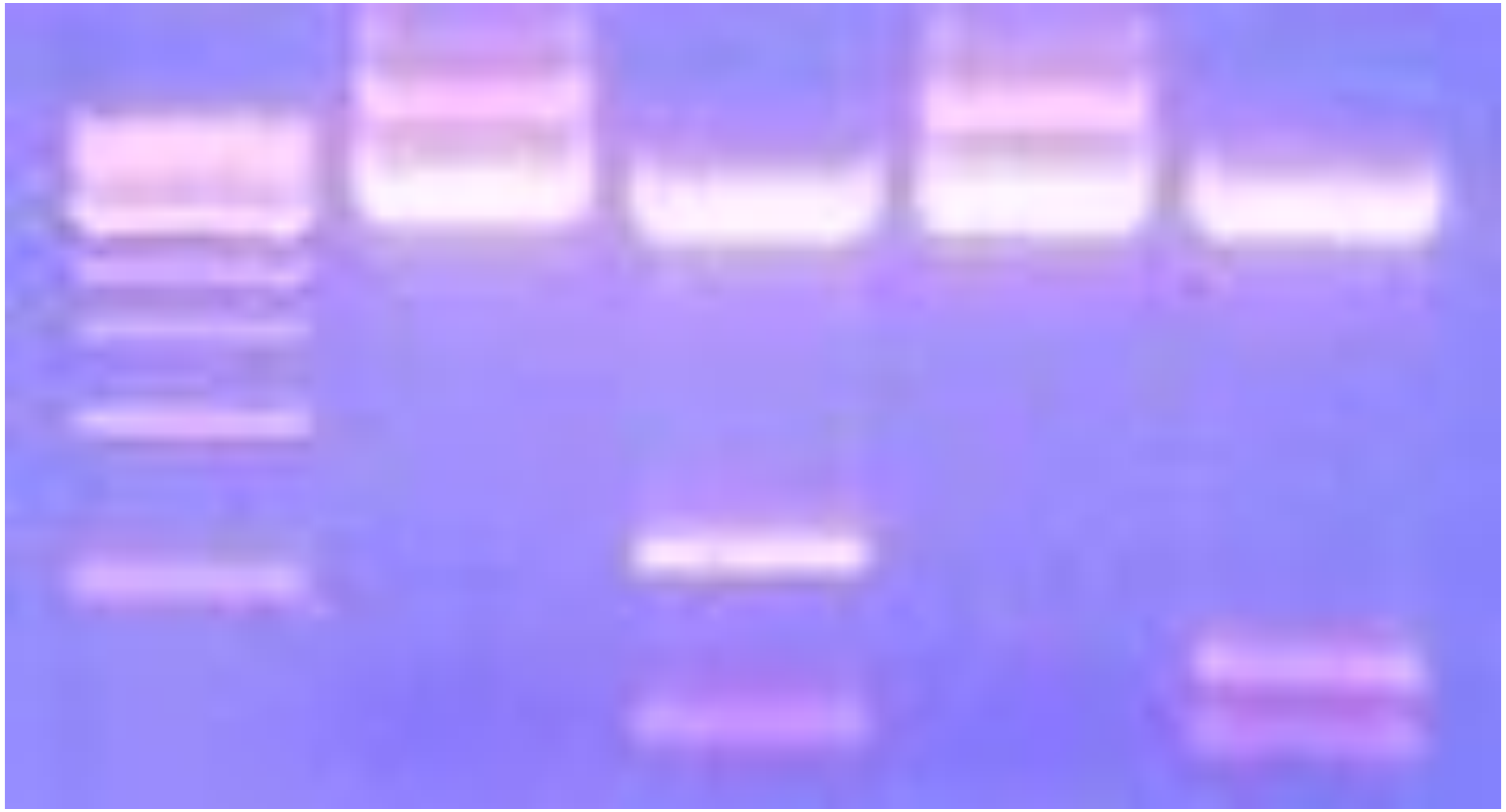

Figure 10. White Gene Coding and Digest Gel. A.

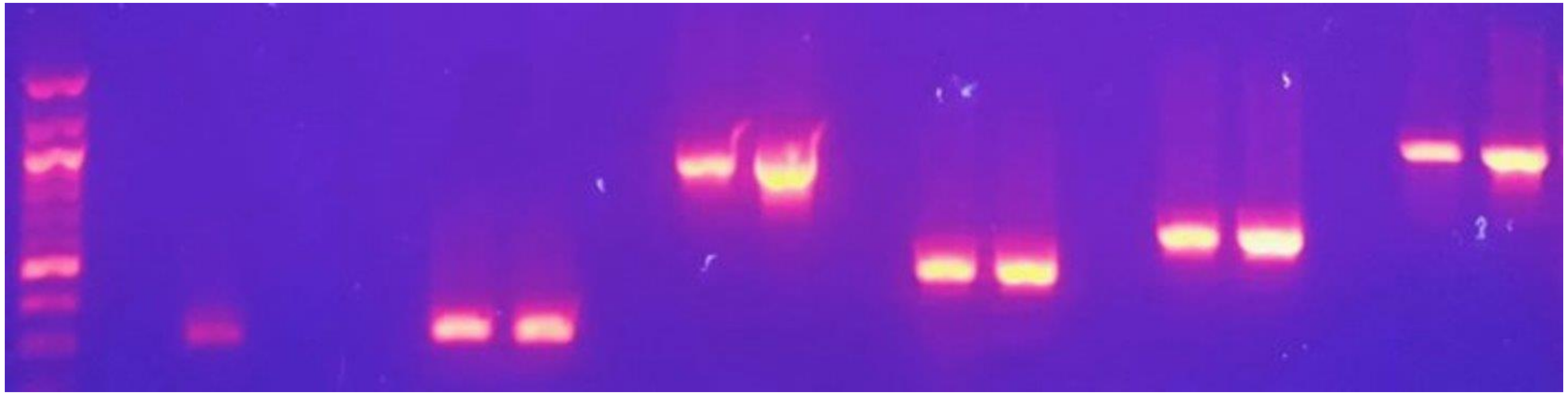

Figure 10. White Gene Coding and Digest Gel. B.

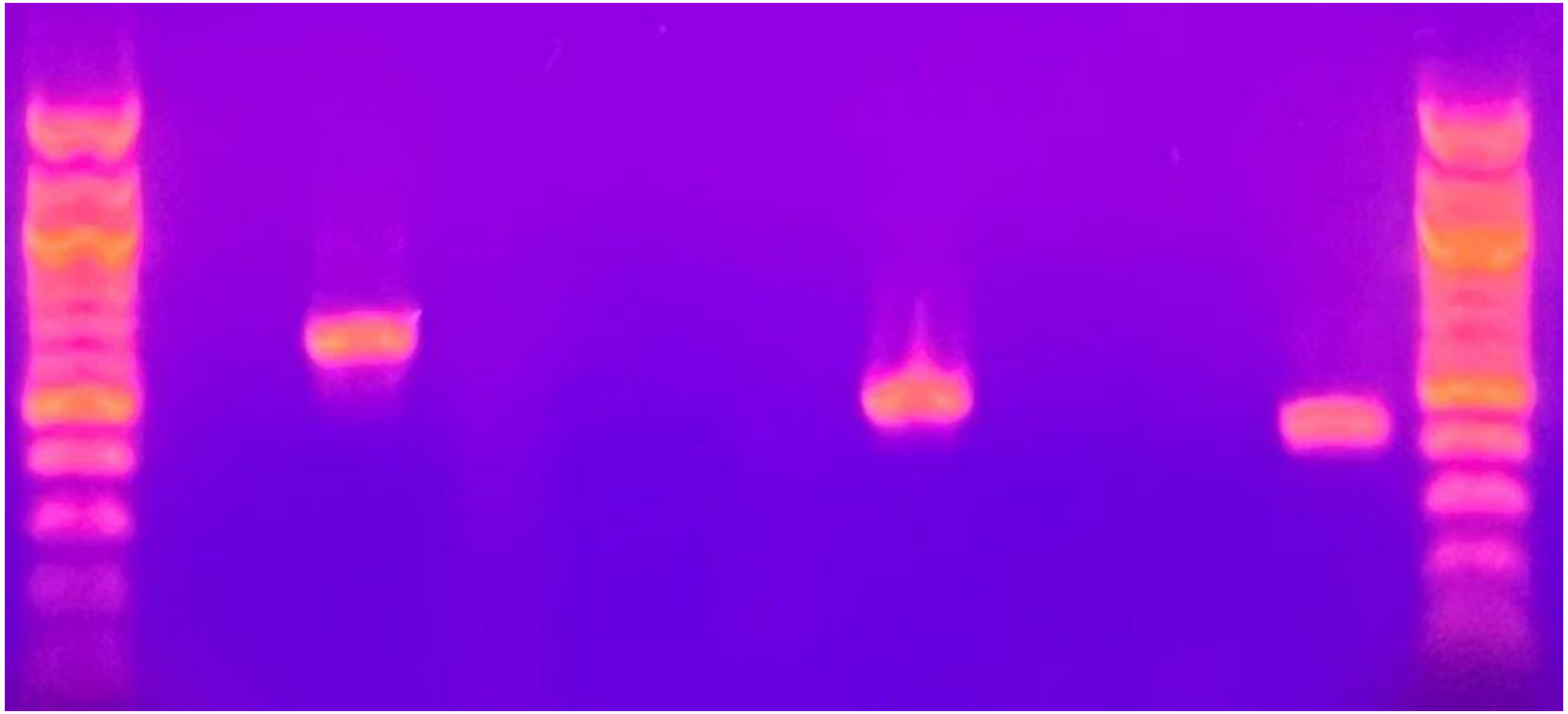

Figure 10. White Gene Coding and Digest Gel. C and D.

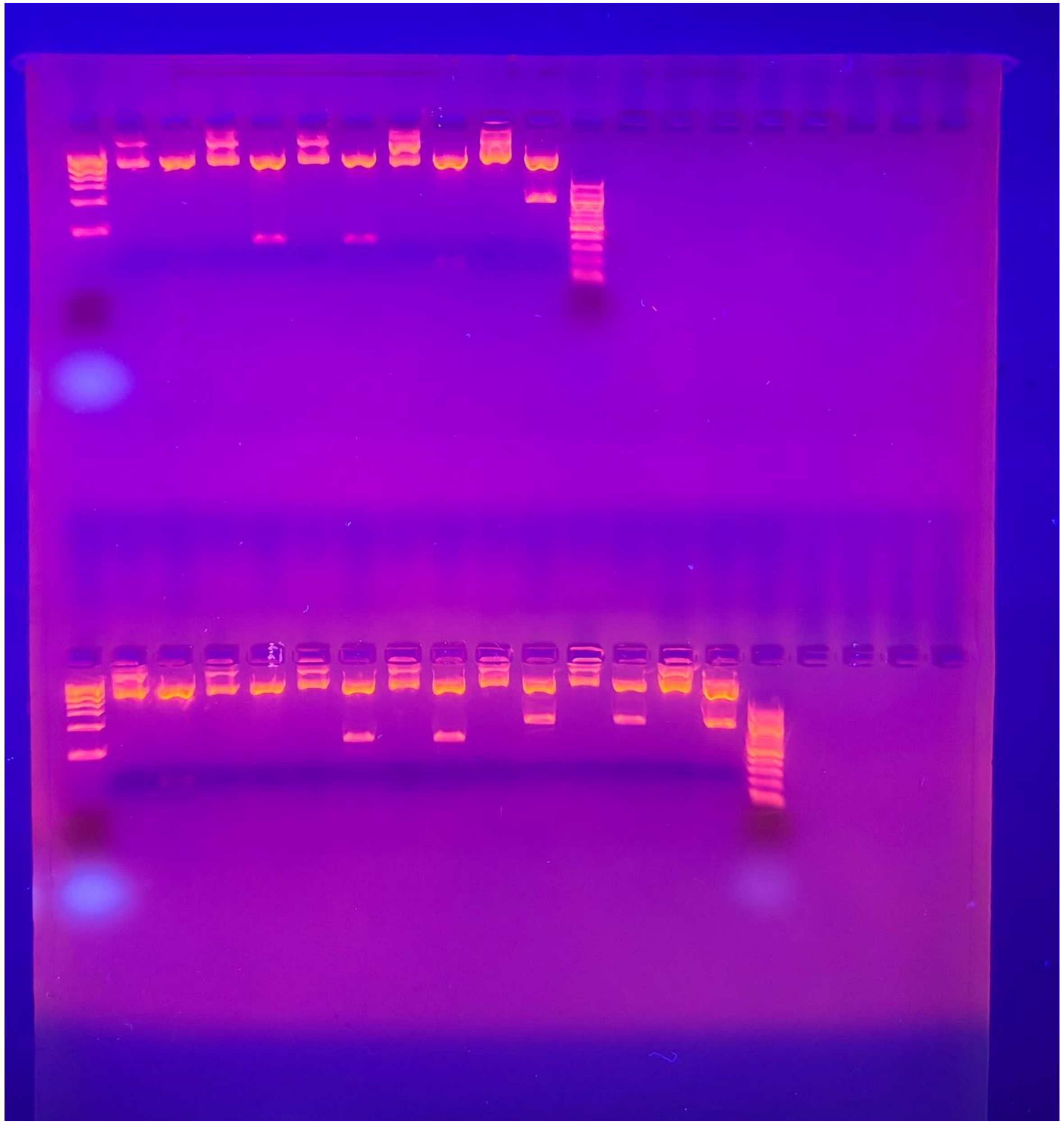

Figure 10. White Gene Coding and Digest Gel. E.

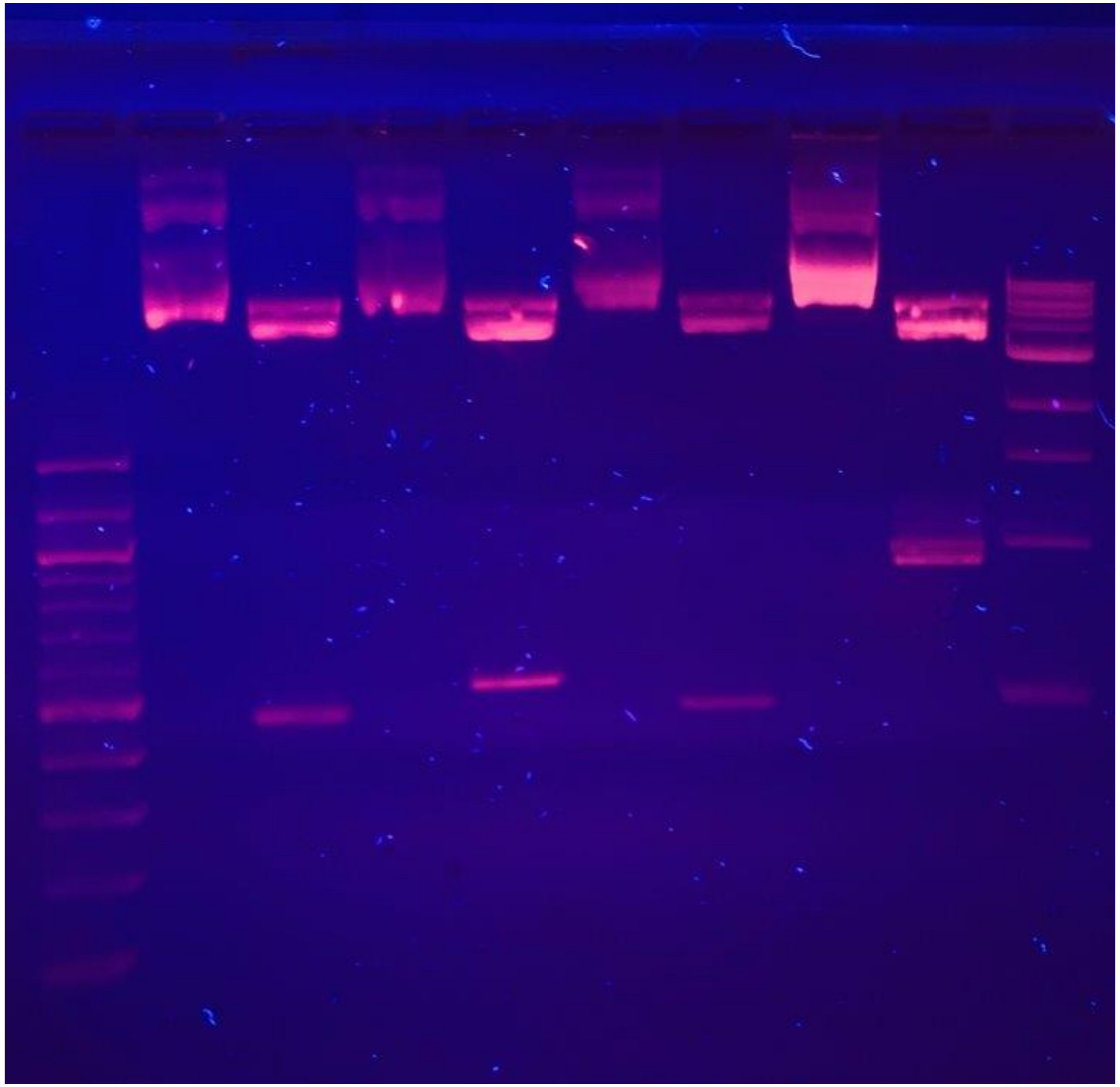

Figure 12. Yellow Gene Coding and Digest Gels. A.

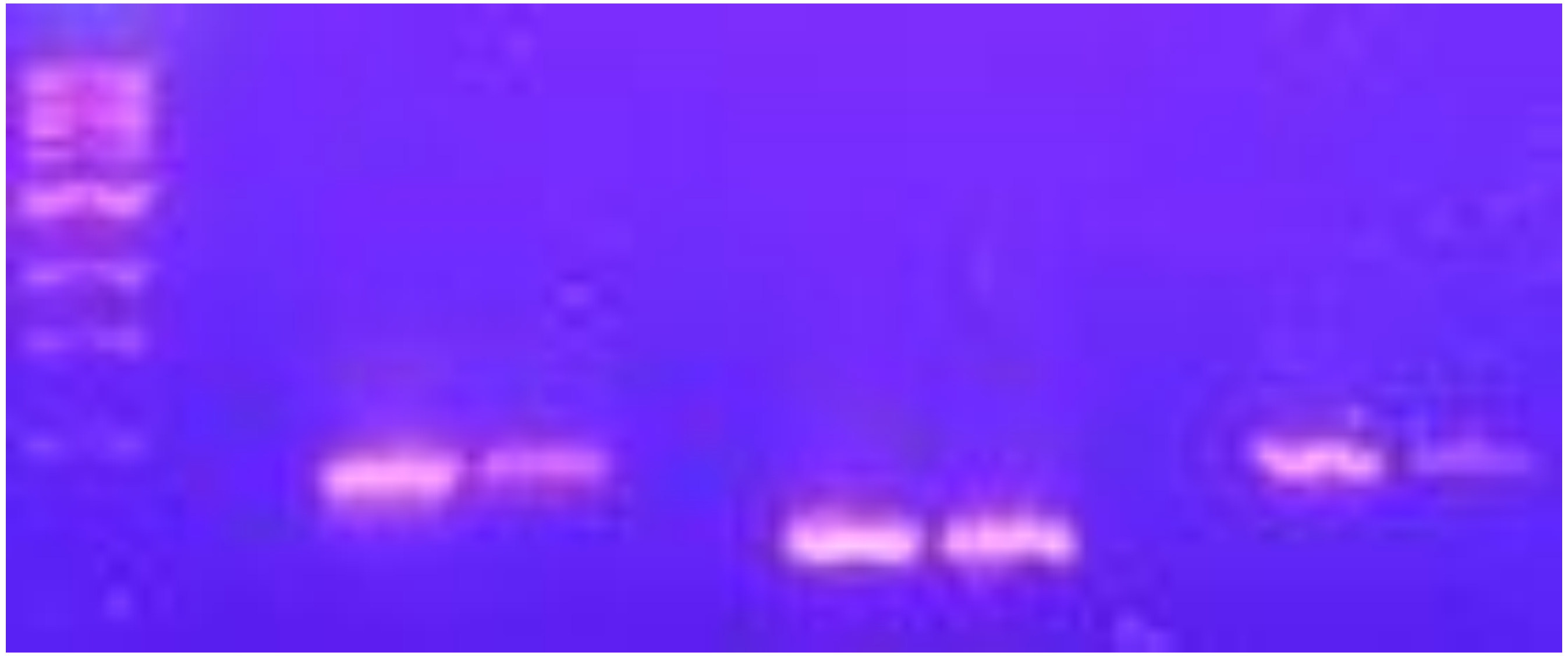

Figure 12. Yellow Gene Coding and Digest Gels. A.

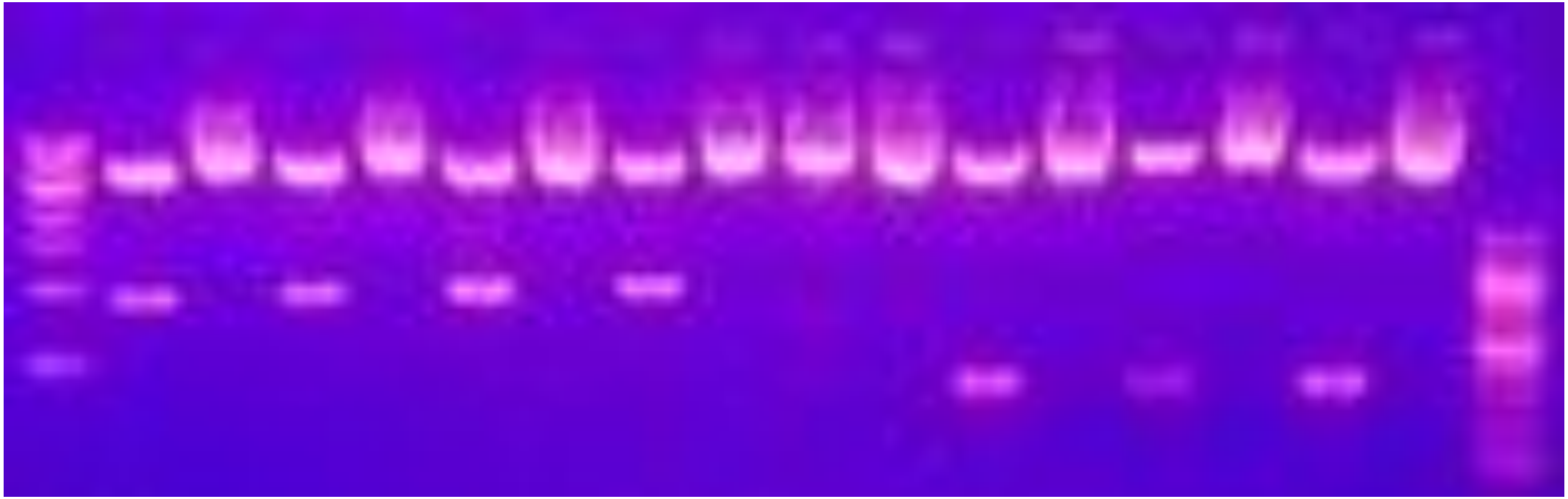

Figure 12. Yellow Gene Coding and Digest Gels. C.

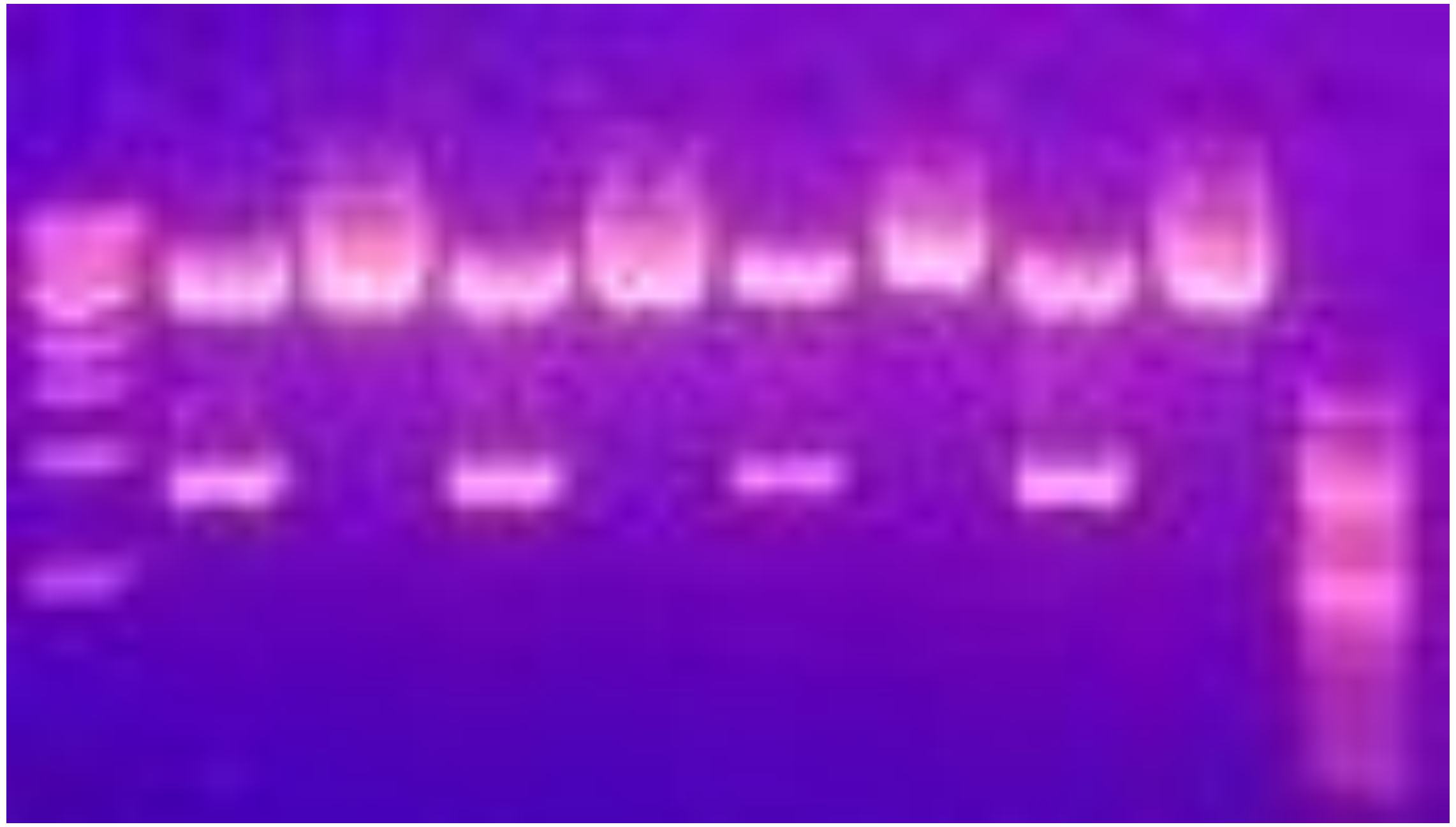

Figure 14. Vestigial Coding and Digest Gels. A and B.

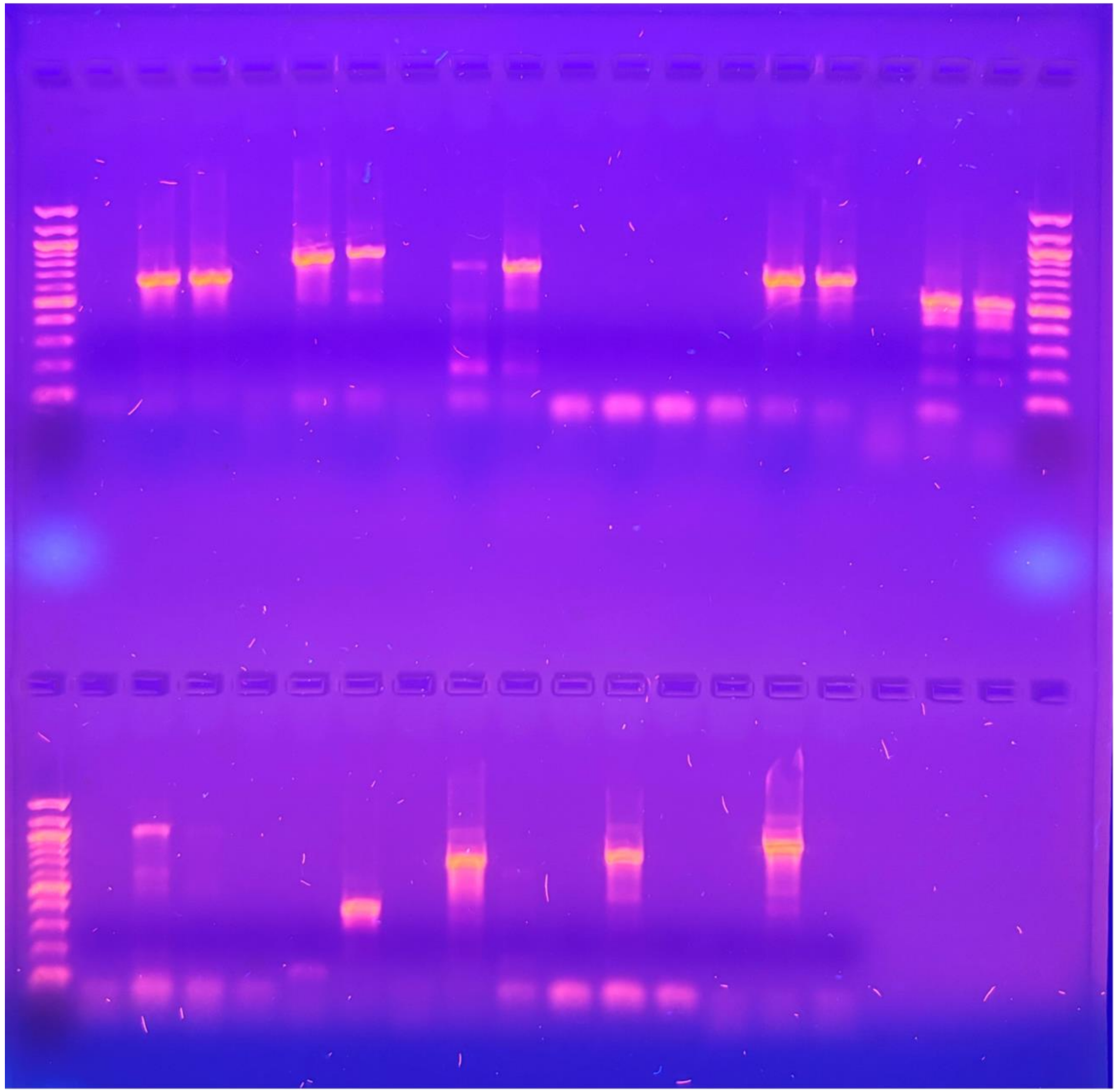

Figure 14. Vestigial Coding and Digest Gels. C and D.

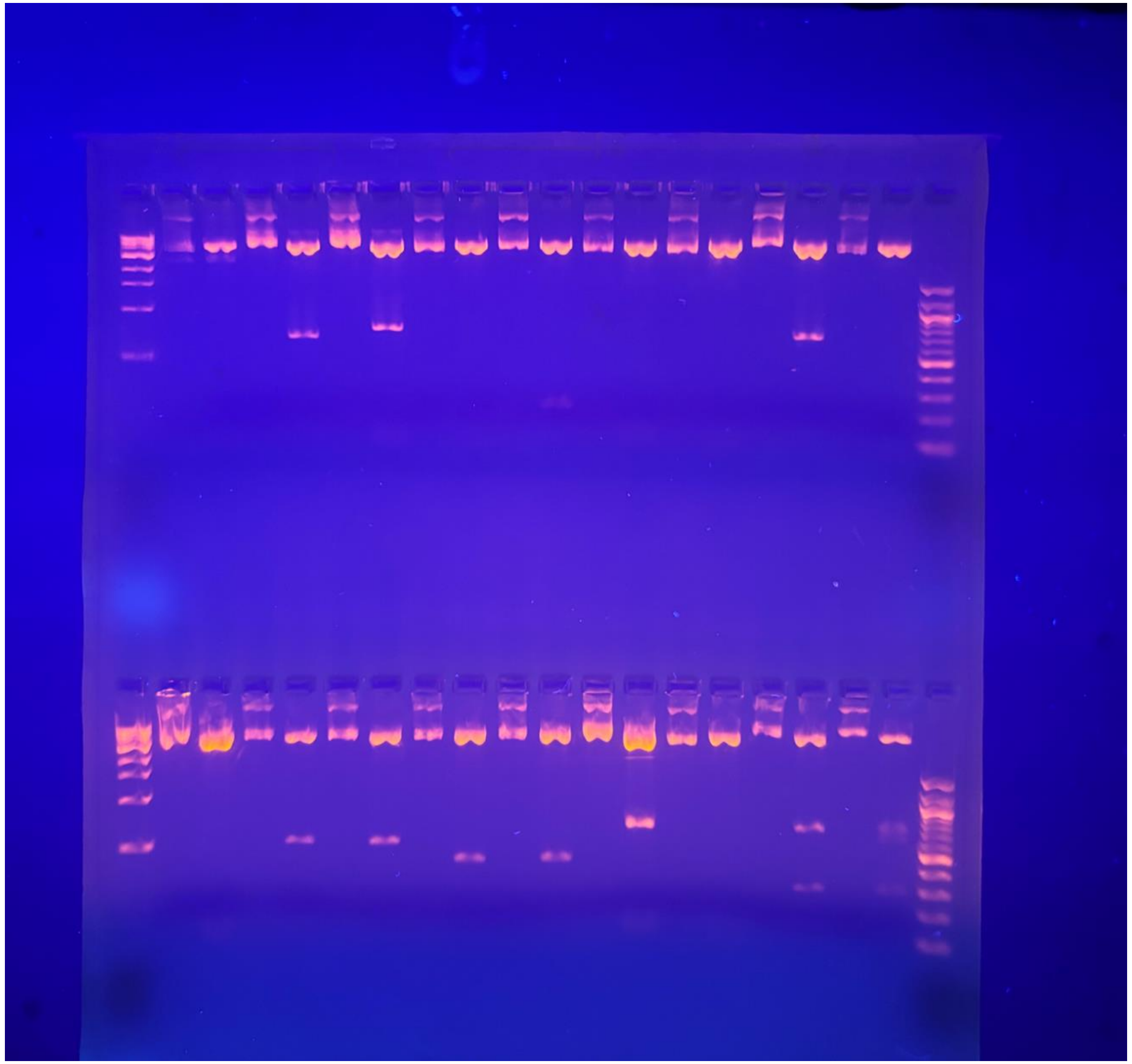

Supplement: Supplementary file 1 [file pone.0356998.s001.pdf]
