# Supplementary material for: Identifying DNA mutations in Sepia, White, Yellow, and Vestigial genes in D. melanogaster - Integrating inquiry-based experiential learning into undergraduate Genetics Laboratory curriculum
Source: PLoS One. 2026 Sep 1;21(9):e0356998. doi: 10.1371/journal.pone.0356998 (PMC13533379; doi:10.1371/journal.pone.0356998)
Supplement: Supplementary file 2 [file pone.0356998.s003.pdf]

Sepia compiled cDNA sequences

Replica sequencing analyses within the lab section were compared to determine consensus sequence for each of 4 inserts examined: WT primer set 1 product, WT primer set 2 product, MUT primer set 1 product, MUT primer set 2 product

- Any sequences in reverse orientation were reverse complemented
- If any discrepancies were found within replica sequence data, sequence results with highest quality reads (chromatogram) were utilized to generate "consensus" sequence from data obtained

- Where quality reads were obtained: Sequencing data from primer sets 1 and 2 were combined for WT; Sequencing data from primer sets 1 and 2 were combined for MUT
  - Overlapping sequence from primer sets 1 and 2 is represented here as combined consensus
- 5' UTR sequence removed (each file starts with Methionine ATG start codon)
- Intronic sequences removed (Introns 1 and 2)
- 3' UTR and sequence through to Primer set 2 reverse primer binding location were retained where possible
- Partial cDNA sequences may be provided if any coding regions were not successfully sequenced

Primer set 1 data   Primer set 2 data

Consensus sequence from database:

>Database
ATGAGTAACGGCAGGCATTTGGCAAAAGGCTCACCCATGCCGGATGTTCCCGAAGATGGTATCCTTCGCCTGTACTCGATGCG
CTTCTGCCCATTTGCCCAACGGGTGCATCTGGTCCTGGACGCCAAGCAGATCCCGTATCACAGCATCTACATTAATCTCACAGA
CAAGCCGGAGTGGCTGCTGGAGAAGAATCCACAGGGCAAGGTGCCGGCTCTAGAAATCGTGCGAGAACCTGGACCACCTGT
GCTCACAGAGTCGCTACTGATTTGTGAATATCTGGACGAGCAGTATCCATTGCGACCACTCTATCCACGTGATCCGCTGAAGAA
AGTGCAGGACAAGTTACTAATCGAGCGATTTAGAGCGGTGTTAGGTGCCTTCTTCAAGGCATCCGATGGCGGTGATCTGGAGC
CCTTCTGGAGCGGCCTGGACATCTACGAAAGGGAGCTGGCTCGACGTGGTACGGAATTCTTTGGTGGCGAGCAGACGGGCAT
TCTGGACTATATGATTTGGCCCTGGTGTGAGCGCCTCGAGCTCCTTAAGTTGCAGCGTGGAGAGGATTATAACTACGATCAGA
GTCGCTTCCCCCAGCTGACCCTTTGGCTGGAACGCATGAAACGAGATCCGGCTGTGATGGCCTTCTACATGGAGGCCGAGGTT
CAGGCGGAGTTCCTGCGTACACGGAGCCTGGGTCGACCCAATTACAATCTGCTGGTCAAGGATGCCTGATGCACTTGGTTTAT
TGAATTTTTCGCATGGAATGAATAAAACATAAATGCAGTCCATAACCGGTTCCAAAGATTTGATCTTGTCTAAACCAGTTGTCG
TTCTATGGTGTACCCTTGAAGGCAATGTCGTACTGGGGATTTCCAAGGGTCTTCGATTTCTGGAACTCAGCATGCAACTGGACA
TCCAAAGCTGTCTTCTGCACAACCTCATCCTGGGT

WT sepia results:

>WT
ATGAGTAACGGCAGGCATTTGGCAAAAGGCTCACCCATGCCGGATGTTCCCGAAGATGGTATCCTTCGCCTGTACTCGATGCG
CTTCTGCCCATTTGCCCAACGGGTGCATCTGGTCCTGGACGCCAAGCAGATCCCGTATCACAGCATCTACATTAATCTCACAGA
CAAGCCGGAGTGGCTGCTGGAGAAGAATCCACAGGGCAAGGTGCCGGCTCTAGAAATCGTGCGAGAACCTGGACCACCTGT
GCTCACAGAGTCGCTACTGATTTGTGAATATCTGGACGAGCAGTATCCATTGCGACCACTCTATCCACGTGATCCGCTGAAGAA
AGTGCAGGACAAGTTACTAATCAAGCGATTTAGAGCGGTGTTAGGTGCCTTCTTCAAGGCATTCGATGGCGGTGATCTGGAGC
CCTTCTGGAGCGGCCTGGACATCTACGAAAGGGAGCTGGCTCGACGTGGTACGGAATTCTTTGGTGGCGAGCAGACGGGCAT
TCTGGACTATATGATTTGGCCCTGGTGTGAGCGCCTCGAGCTCCTTAAGTTGCAGCGTGGAGAGGATTATAACTACGATCAGA
GTCGCTTCCCCCAGCTGACCCTTTGGCTGGAACGCATGAAACGAGATCCGGCTGTGATGGCCTTCTACATGGAGGCCGAGGTT
CAGGCGGAGTTCCTGCGTACACGGAGCCTGGGTCGACCCAATTACAATCTGCTGGTCAAGGATGCCTGATGCACTTGGTTTAT

TGAATTTTTCGCATGGAATGAATAAAACATAAATGCAGTCCATAACCGGTTCCAAAGATTTGATCTTGTCTAAACCAGTTGTCG
TTCTATGGTGTACCCTTGAAGGCAATGTCGTACTGGGGATTTCCAAGGGTCTTCGATTTCTGGAACTCAGCATGCAACTGGACA
TCCAAAGCTGTCTTCTGCACAACCTCATCCTGGGTCATCAAATCGCGCCACTTGAG

MUT sepia (Se) results:

>MUT
ATGAGTAACGGCAGGCATTTGGCAAAAGGCTCACCCATGCCGGATGTTCCCGAAGATGGTATCCTTCGCCTGTACT
CGATGCGCTTCTGCCCATTTGCCCAACGGGTGCATCTGGTCCTGGACGCCAAGCAGATCCCGTATCACAGCATCTA
CATTAATCTCACAGACAAGCCGGAGTGGCTGCTGGAGAAGAATCCACAGGGCAAGGTGCCGGCTCTAGAAATCGT
GCGAGAACCTGGACCACCTGTGCTCACAGAGTCGCTACTGATTTGTGAATATCTGGACGAGCAGTATCCATTGCGA
CCACTCTATCCACGTGATCCGCTGAAGAAAGTGCAGGACAAGTTACTAATCGAGCGATTTAGAGCGGTGTTAGGT
GCCTTCTTCAAGGCATCCGATGGCGGTGATCTGGAGCCCTTCTGGAGCGGCCTGGACATCTGCGAAAGGGAGCTG
GCTCGACGTGGTACGGAATTCTTTGGTGGCGAGCAGACGGGCATTCTGGACTATATGATTTGGCCCTGGTGTGAG
CGCCTCGAGCTCCTTAAGTTGCAGCGTGGAGAGGATTATAACTACGATCAGAGTCGCTTCCCCCAGCTGACCAGTT
GTCGTTCTATGGTGTACCCTTGAAGGCAATGTCGTACTGGGGATTTCCAAGGGTCTTCGATTTCTGGAACTCAGCA
TGCAACTGGACATCCAAAGCTGTCTTCTGCACAACCTCATCCTGGGTCATCAAATCGCGCCACTTGAG
