# Supplementary material for: Identifying DNA mutations in Sepia, White, Yellow, and Vestigial genes in D. melanogaster - Integrating inquiry-based experiential learning into undergraduate Genetics Laboratory curriculum
Source: PLoS One. 2026 Sep 1;21(9):e0356998. doi: 10.1371/journal.pone.0356998 (PMC13533379; doi:10.1371/journal.pone.0356998)
Supplement: Supplementary file 3 [file pone.0356998.s004.pdf]

Yellow compiled cDNA sequences

- Sequencing data from primer set 1, 2, and 3 combined
    - Any sequences in reverse orientation were reverse complemented
    - Overlapping sequence from primer set 2 & 3 product data is represented in the full cDNA file as combined consensus
- 5' UTR sequence has been removed
- 3' UTR sequence, beyond the stop codon, has been removed
- Intronic sequences have been removed (Intron 1)

Primer set 1 data (1-238) Primer set 2 data (239-802) Primer set 3 data (803-1626)

Consensus sequence from database:
The start codon sequence here has been updated to ATG as the y1 mutant allele (A to C mutation in start codon) was used as the reference strain in the current database

>Database
ATGTTCCAGGACAAAGGGTGGATCCTTGTGACCCTGATCACCTTGGTGACGCCGTCTTGGGCTGCTTACAAACTTCAGGAGCG
ATATAGTTGGAGCCAGCTGGACTTTGCTTTCCCGAATACCCGACTAAAGGACCAAGCTCTGGCTAGTGGAGATTATATTCCGCA
AAATGCTCTACCTGTTGGAGTCGAACACTTTGGCAATCGGTTATTCGTCACTGTTCCCCGCTGGCGTGATGGGATTCCGGCCAC
TCTGACCTATATAAACATGGACCGCAGTTTGACGGGTTCACCGGAGCTAATTCCGTATCCAGATTGGCGCTCAAATACAGCTGG
AGATTGCGCCAACAGTATTACCACTGCCTACCGCATTAAAGTGGATGAGTGTGGTCGGCTGTGGGTTTTGGACACTGGAACCG
TGGGCATCGGCAATACCACCACTAATCCGTGCCCCTATGCGGTAAATGTCTTTGACTTGACCACGGATACGCGAATTCGGAGAT
ACGAGCTACCTGGCGTGGACACAAATCCAAATACTTTCATAGCTAACATTGCCGTGGATATAGGCAAAAATTGCGATGATGCA
TATGCCTATTTTGCCGATGAATTGGGATACGGCTTGATTGCTTACTCCTGGGAACTGAACAAGTCCTGGAGATTCTCGGCACAT
TCGTATTTTTTCCCCGATCCATTGAGGGGCGATTTCAATGTCGCTGGTATTAACTTCCAATGGGGCGAGGAGGGTATATTTGGT
ATGTCCCTTTCGCCCATTCGATCGGATGGTTATCGTACCCTGTACTTTAGTCCGTTAGCAAGTCATCGACAATTTGCCGTATCCA
CGAGGATTTTGAGGGATGAAACCAGGACGGAAGATAGCTATCATGACTTTGTTGCCTTAGATGAACGGGGTCCAAACTCCCAT
ACCACTTCACGTGTGATGAGCGATGATGGAATTGAGCTGTTCAATTTAATAGATCAAAATGCAGTGGGTTGCTGGCACTCATC
AATGCCGTACTCACCGCAATTTCATGGCATTGTGGATCGCGATGACGTTGGCTTAGTTTTTCCGGCCGATGTGAAAATTGATGA
GAACAAAAACGTTTGGGTTCTATCCGATAGGATGCCCGTTTTCTTGCTGTCTGACTTGGATTATTCAGATACTAATTTCCGAATT
TACACGGCTCCCTTGGCCACTTTAATTGAGAATACTGTGTGTGATTTGAGGAATAACGCCTATGGGCCGCCAAATACCGTTTCA
ATACCAAAACAAGCCGTTTTGCCAATGGGTCCACCGTTATATACGAAACAATATCGTCCTGTCTTGCCACAGAAACCTCAGACC
AGCTGGGCTTCCTCGCCGCCTCCTCCAAGTCGCACTTATTTGCCCGCCAATTCAGGCAATGTAGTCTCCAGTATTAGTGTCTCTA
CAAATTCTGTGGGTCCTGCAGGAGTGGAGGTGCCAAAGGCCTATATTTTCAACCAGCACAACGGCATAAATTACGAGACAAGT
GGTCCCCATCTATTTCCCACCCATCAACCCGCCCAACCGGGTGGCCAGGATGGTGGGTTAAAAACTTATGTGAATGCCCGCCAA
TCTGGGTGGTGGCATCATCAGCATCAAGGTTAACATAAT

WT yellow results:
ATGTTCCAGGACAAAGGGTGGATCCTTGTGACCCTGATCACCTTGGTGACGCCGTCTTGGGCTGCTTACAAACTTCAGGAGCG
ATATAGTTGGAGCCAGCTGGACTTTGCTTTCCCGAATACCCGACTAAAGGACCAAGCTCTGGCTAGTGGAGATTATATTCCGCA
AAATGCTCTACCTGTTGGAGTCGAACACTTTGGCAATCGGTTATTCGTCGCTGTTCCCCGCTGGCGTGATGGGATTCCGGCCAC
TCTGACCTATATAAACATGGACCGCAGTTTGACGGGTTCACCGGAGCTAATTCCGTATCCAGATTGGCGCTCAAATACAGCTGG
AGATTGCGCCAACAGTATTACCACTGCCTACCGCATTAAAGTGGATGAGTGTGGTCGGCTGTGGGTTTTGGACACTGGAACCG
TGGGCATCGGCAATACCACCACTAATCCGTGCCCCTATGCGGTAAATGTCTTTGACTTGACCACGGATACGCGAATTCGGAGAT
ACGAGCTACCTGGCGTGGACACAAATCCAAATACTTTCATAGCTAACATTGCCGTGGATATAGGCAAAAATTGCGATGATGCA
TATGCCTATTTTGCCGATGAATTGGGATACGGCTTGATTGCTTACTCCTGGGAACTGAACAAGTCCTGGAGATTCTCGGCACAT
TCGTATTTTTTCCCCGATCCATTGAGGGGCGATTTCAATGTCGCTGGTATTAACTTCCAATGGGGCGAGGAGGGTATATTTGGT

ATGTCCCTTTCGCCCATTCGATCGGATGGTTATCGTACCCTGTACTTTAGTCCGTTAGCAAGTCATCGACAATTTGCCGTATCCA
CGAGGATTTTGAGGGATGAAACCAGGACGGAAGATAGCTATCATGACTTTGTTGCCTTAGATGAACGGGGTCCAAACTCCCAT
ACCACTTCACGTGTGATGAGCGATGATGGAATTGAGCTGTTCAATTTAATAGATCAAAATGCAGTGGGTTGCTGGCACTCATC
AATGCCGTACTCACCGCAATTTCATGGCATTGTGGATCGCGATGACGTTGGCTTAGTTTTTCCGGCCGATGTGAAAATTGATGA
GAACAAAAACGTTTGGGTTCTATCCGATAGGATGCCCGTTTTCTTGCTGTCTGACTTGGATTATTCAGATACTAATTTCCGAATT
TACACGGCTCCCTTGGCCACTTTAATTGAGAATACTGTGTGTGATTTGAGGAATAACGCCTATGGGCCGCCAAATACCGTTTCA
ATACCAAAACAAGCCGTTTTGCCAATGGGTCCACCGTTATATACGAAACAATATCGTCCTGTCTTGCCACAGAAACCTCAGACC
AGCTGGGCTTCCTCACCGCCTCCTCCAAGTCGCACTTATTTGCCCGCCAATTCAGGCAATGTAGTCTCCAGTATTAGTGTCTCTA
CAAATTCTGTGGGTCCTGCAGGAGTGGAGGTGCCAAAGGCCTATATTTTCAACCAGCACAACGGCATAAATTACGAGACAAGT
GGTCCCCATCTATTTCCCACCCATCAACCCGCCCAACCGGGTGGCCAGGATGGTGGGTTAAAAACTTATGTGAATGCCCGCCAA
TCTGGGTGGTGGCATCATCAGCATCAAGGTTAA

<u>MUT yellow (Y) results</u>:

>MUT
CTGTTCCAGGACAAAGGGTGGATCCTTGTGACCCTGATCACCTTTGGTGACGCCGTCTTGGGCTGCTTACAAACTTCAGGAGC
GATATAGTTGGAGCCAGCTGGACTTTGCTTTCCCGAATACCCGACTAAAGGACCAAGCTCTGGCTAGTGGAGATTATATTCCAC
AAAATGCTCTACCTGTTGGAGTCGAACACTTTGGCAATCGGTTATTCGTCACTGTTCCCCGCTGGCGTGATGGGATTCCGGCCA
CTCTGACCTATATAAACATGGACCGCAGTTTGACGGGTTCACCGGAGCTAATTCCGTATCCAGATTGGCGCTCAAATACAGCTG
GAGATTGCGCCAACAGTATTACCACTGCCTACCGCATTAAAGTGGATGAGTGTGGTCGGCTGTGGGTTTTGGACACTGGAACC
GTGGGCATCGGCAATACCACCACTAATCCGTGCCCCTATGCGGTAAATGTCTTTGACTTGACCACGGATACGCGAATTCGGAG
ATACGAGCTACCTGGCGTGGACACAAATCCAAATACTTTCATAGCTAACATTGCCGTGGATATAGGCAAAAATTGCGATGATG
CATATGCCTATTTTGCCGATGAATTGGGATACGGCTTGATTGCTTACTCCTGGGAACTGAACAAGTCCTGGAGATTCTCGGCAC
ATTCGTATTTTTTCCCCGATCCATTGAGGGGCGATTTCAGTGTCGCTGGTATTAACTTCCAATGGGGCGAGGAGGGTATATTTG
GTATGTCCCTTTCGCCCATTCGATCGGATGGTTATCGTACCCTGTACTTTAGTCCGTTAGCAAGTCATCGACAATTTGCCGTATC
CACGAGGATTTTGAGGGATGAAACCAGGACGGAAGATAGCTATCATGACTTTGTTGCCTTAGACGAACGGGGTCCAAACTCCC
ATACCACTTCACGTGTGATGAGCGATGATGGAATTGAGCTGTTCAATTTAATAGATCAAAATGCAGTGGGTTGCTGGCACTCAT
CAATGCCGTACTCACCGCAATTTCATGGCATTGTGGATCGCGATGACGTTGGCTTAGTTTTTCCGGCCGATGTGAAAATTGATG
AGAACAAAAACGTTTGGGTTCTATCCGATAGGATGCCCGTTTTCTTGCTGTCTGACTTGGATTATTCAGATACTAATTTCCGAAT
TTACACGGCTCCCTTGGCCACTTTAATTGAGAATACTGTGTGTGATTTGAGGAATAACGCCTATGGGCCGCCAAATACCGTTTC
AATACCAAAACAAGCCGTTTTGCCAATGGGTCCACCGTTATATACGAAACAATATCGTCCTGTCTTGCCACAGAAACCTCAGAC
CAGCTGGGCTTCCTCGCCGCCTCCTCCAAGTCGCACTTATTTGCCCGCCAATTCAGGCAATGTAGTCTCCAGTATTAGTGTCTCT
ACAAATTCTGTGGGTCCTGCAGGAGTGGAGGTGCCAAAGGCCTATATTTTCAACCAGCACAACGGCATAAATTACGAGACAAG
TGGTCCCCATCTATTTCCCACCCATCAACCCGCCCAACCGGGTGGCCAGGATGGTGGGTTAAAAACTTATGTGAATGCCCGCCA
ATCTGGGTGGTGGCATCATCAGCATCAAGGTTAA
