# Supplementary material for: Identifying DNA mutations in Sepia, White, Yellow, and Vestigial genes in D. melanogaster - Integrating inquiry-based experiential learning into undergraduate Genetics Laboratory curriculum
Source: PLoS One. 2026 Sep 1;21(9):e0356998. doi: 10.1371/journal.pone.0356998 (PMC13533379; doi:10.1371/journal.pone.0356998)
Supplement: Supplementary file 4 [file pone.0356998.s005.pdf]

Genetics Lab Report Guidelines

Although general discussion of the experiments among students is acceptable and encouraged and some experimental data will be shared, **the writing of all sections of reports and figure labels/legends must be done individually**. Please refer to the Undergraduate Handbook & the syllabus for the consequences of plagiarism. ALWAYS **SUMMARIZE IN YOUR OWN WORDS AND APPROPRIATELY REFERENCE SOURCES UTILIZED.** If you have any questions or concerns, please discuss with your TA/Instructor ***before turning in your report***. If you took this class previously, be sure to completely re-write all reports.

Formatting: Lab reports must be typed, double-spaced & in 11-12 point font as a word doc. Please spell-check and proofread your work as writing and mechanics is assessed in the grading rubric for all lab reports. Use headings for each section. Formal writing should not mirror casual conversation. Use the past tense to describe experiments that were done. Refer to the individual grading rubrics and instructions for more specific directions pertaining to each report. In addition, please follow these general guidelines for all reports:

General Guidelines:

* NO QUOTATIONS. Text quotations are typically not utilized in scientific writing. **Always restate in your own words (paraphrase), AND reference the source of information (in-text and in reference section).**
* Only utilize credible sources. Examples include: text books, your lab manual, and scientific journal articles. If you are uncertain about a particular source, ask your TA before you start writing your report. The lab manual materials provided include some basic background, but students are expected to use additional resources, properly cited.
* You MUST include citations throughout the text of your report. Failure to include in-text citations (e.g. Smith *et al*., 2013) **is a form of plagiarism**!
* You will be using tables &/or figures to display and help explain your data results. Tables and figures MUST be fully and properly labeled, with a written legend & an explanation of the results in the main body of the text.
* Please include page numbers in the main body of the report.
* Target audience: write so that a peer with some basic scientific knowledge, but who has not yet taken this course, could understand.
* This is a scientific record of your journey during lab. It is a record of what YOU did, your particular experimental procedure, the results of the experiments performed, and your interpretation of your results. This may often include deviations from the lab manual directions as instructed by your TA or may indicate if/where mistakes were made. It needs to be written objectively, with no personal feelings or bias.
* Remember, a hypothesis is a proposed answer or explanation that is testable. There should be rationale for forming your hypothesis, based on what you know and/or a previous observation. It is NOT a random guess. Through your experiments, you will test your hypothesis. Your experimental findings may or may not support your hypothesis, this is what you seek to determine.

A formal laboratory report includes all of the following sections. *Please pay attention to individual lab report guidelines. Lab report 1 is submitted as a partial report. Lab report 2 will be submitted first as a partial report draft AND then as a full lab report.*

Title Page: The title of the experiment, your name, your group members' names, the date, and the course title with section number should be included.

Introduction: Clearly state the purpose of the experiment(s), identify the subject matter, and explain the major biological or genetic principles being examined so that the reader can understand the work presented. Define the problem/question addressed and provide rationale and clear statement of the hypothesis leading into the study. Introduce the experimental model system utilized to test the hypothesis and justify the experimental design. State the anticipated results given your hypothesis and discuss the rationale (reason) for expecting this outcome. Your goal is to clearly and concisely write the information in ~2 pages. ***You will need frequent in-text citations in this section of the report as most material will be sourced from your references.***

Materials and Methods: This section should be divided into sub-sections with sub-headings and written in paragraph form using concise sentences (Methods & Materials is <u>not</u> a bulleted list of what you did). It is a narrative of your procedure performed in lab. Do not include unnecessary detail, but be thorough enough so that someone else could understand and repeat the procedure. For example, provide reagents used, concentrations, incubation times and other specific conditions that apply. You should include any modifications or deviations from the protocol presented in the laboratory manual. ***The lab manual will be cited here, but this section must be written in your own words – not directly copied from the lab manual!***

Results: Results reflect what was actually done in the lab and should be presented clearly and concisely in **written text**, composed of complete paragraphs. Briefly remind the reader what experiments were performed to obtain each result presented. In the text, call attention to the important parts of the data and relevant tables or figures. Avoid in-depth discussion of the meaning or interpretation of your results in this section, but be sure to clearly state all results obtained (for experimental and control samples). Data should also be presented in properly labeled and organized tables and figures where appropriate. These should be labeled, both numerically and with titles. Data figures and tables should have legends containing sufficient information so that they can stand alone. Be sure to number tables and figures consecutively, and refer to them in the text of this section. <u>If you use a figure/table you must MUST describe it in the results text</u>. ***You may find that you need to in-text cite occasionally in this section, whenever mentioning information you have learned from another source.***

Formatting examples of in-text referral to data provided in figures/tables:

- As shown in Figure 2, the plasmid contained a 2kb insert……

- The plasmid contained a 2kb insert (Figure 2).

How to write a figure legend: The first sentence of a figure legend is the title for the figure. Other information to include (depending on the nature of the data displayed in the figure):

- What is the result/analysis shown, what experimental procedure was used?
- What material/sample are we looking at?
- What important conditions should be mentioned?
- Any definition of abbreviations or additional labels to note/describe?
- Error bars, statistical tests, and significance should be defined (where applicable).
- If figures include multiple parts (e.g. A, B, C), define what is presented in each.

Look at examples from relevant research articles and think about what you need to include, in concise a way as possible, for the reader to understand what is displayed from the figure and figure legend alone.

Discussion: This section is used to explain the meaning or significance of your results and interpretation of the data. In addition, did your results support your hypotheses? Why or why not? Did the results from any controls utilized help you to assess your results and how? Any speculation here when discussing unexpected findings should be based on logical rationale and your understanding of the techniques or genetic principles involved. Unexpected results often arise from explainable causes and should be discussed as best as possible. For this section, even though you are using your own words/thoughts to discuss your experiment, you will still need to refer to background sources to discuss your results & its significance. ***You will need in-text citations in this section of the report as some material here is often sourced from your references.***

References: This section provides an alphabetical list of all reference material that has been cited in your paper, by first author last name. You should include any sources you used to write your report, including the lab manual. **All in-text references should be listed in the reference list, and all references in the list should be cited somewhere in the body of the report.** References should adhere to the following specific formatting style:

Reference list formatting:

Book/Textbook:

Alexander, J. M. 1970. *The Occurrence of Starches in Plants*. Martin Press, New York. 486 pp.

Book chapter:

Alexander, J. M. 1972. The occurrence of starches in higher plants. In: R. R. Jordon and M. M. Taylor (eds.), *Carbohydrate Reserves*. Martin Press, New York. 276 pp.

Journal articles:

Alexander, J. M. 1968. Occurrence of starches in bryophytes. Bot. Gaz. *127*: 176-199.

Alexander, J. M., B. J. Martin, and T. R. Smith. 1969. Occurrence of starches in ferns. Am. J. Bot. *47*: 9-18.

Lab manual example (**be sure to update for your specific semester and year**):

MBS Department. 2023. *Mendelian and Molecular Genetics: Investigation of Wing Development and*

Pigment Biosynthesis Pathways in Drosophila melanogaster. Genetics Laboratory Manual PCB 3063L, Fall 2023. University of South Florida. Pro-Copy, Tampa, FL. Print.

In-text citation (in the body of the report) formatting:
…suggested that carbohydrates are the preferred energy source utilized (Alexander, 1968).
…found starch in all ferns examined (Alexander, 1968; Alexander *et al*., 1969).

One author: Alexander
Two authors: Alexander and Smith
Three or more authors: Alexander *et al*.

In-text lab manual citation format: (MBS, year)

Important Notes: You MUST upload your reports in the proper format to Turnitin through Canvas by the deadline to avoid late penalties. You MUST include citations throughout your text in the report and all writing must be in your own words. DO NOT COPY what is in the lab manual, textbook, etc. Working together with group members, sharing of data, and discussion of results are permitted, but your report MUST BE IN YOUR OWN WORDS. DO NOT USE QUOTATIONS. If you have any questions, please discuss with your TA/Instructor **before** turning in your report.

PCB3063L Lab Report 2: Rough Draft (25pts)

Prior to submitting your Lab Report 2 full lab report, you will be handing in a rough draft of the following sections: **Title Page, Introduction, Materials & Methods, and References**. The purpose of this rough draft is to help you begin writing and provide the opportunity to receive feedback from your TA/Instructor that will help you with the final full draft of the report. **The rough draft should not exceed 4 pages (excluding the title page and reference section).** In addition to the details provided below, follow the general guidelines for lab reports and review the syllabus for turnitin and plagiarism policies.

1) **Title page**: The current working title of your paper, your name, your group members' names, the date, and the course title with section number should be included. You are expected to generate your own title that is complete, accurate, and descriptive of the work. This will be a working title that you may update upon receipt of feedback and collection/analysis of data.

2) **Introduction**: Introduce the research question and purpose of experiments performed, providing sufficient background for the reader to understand the project, including the major biological principles being examined. Once defining the research question your experiments address, provide ***an overview*** of the experimental model and approach (techniques) utilized. State the hypothesis, providing rationale (reason/justification) for the hypothesis based on pertinent observations and information already known from the literature. State the anticipated results given your hypothesis and discuss the rationale for expecting this outcome.

   Your goal is to clearly and concisely write this information, ultimately in ~2 pages. Take a look at some of the research articles you are using for background information to see examples of types and flow of information typical in an introduction. Try to keep the length of the Introduction draft to no more than 3 pages.

   Any information from other sources, including any figures you did not generate yourself, must be properly cited in-text/below the figure AND in the reference section.

3) **Materials & Methods:** The rough draft will include only a few of the sub-sections completed by the time the draft is due. However, keep in mind that the feedback on this style writing from your TA/Instructor can also be applied to other sub-sections not yet completed that will be included in the final full report. Concisely describe the procedures you have carried out thus far, including genomic DNA extraction and PCR (you may choose to include a separate section on gel electrophoresis as well). Each sub-section should include a sub-heading and brief synopsis of what you did in your own words in the past tense, including necessary details for someone else to be able to repeat the experiment. Include anything that you did differently from that presented in the lab manual.

   Look at research articles you have found for examples of methods/materials style research paper writing. It is detailed, but very concise, in short paragraph form. **The lab manual will be cited in-text here, and writing must be paraphrased, in your own words, not copied directly from the manual.**

   Just for this section only, you may choose to include a brief statement at the start of the materials and methods to note that the following procedures were adapted from the MBS

lab manual (MBS, year) instead of providing in-text citations throughout the materials and methods sections.

4) **References**: You should include any sources you have used so far to write your draft, including the lab manual. You should use at *least* **3 scientific** sources in your draft, with at least 4 total being required for the final report (**at least two of your sources being journal articles**). Carefully follow the formatting rules for in-text citations and reference section listings.

   Websites are typically not appropriate sources.

   Reminder that you have several relevant, suggested review and primary research article references provided in the course powerpoint slides.

Important Notes: Upload your report to Turnitin by the deadline to avoid late penalties. Your lab report will not be graded unless uploaded to Turnitin through Canvas. You MUST include citations throughout your text in the report and all writing must be in your own words. DO NOT COPY what is written in the lab manual, textbook, online resources, etc. Working together with group members, sharing of data, and discussion of results are permitted, but your report MUST BE IN YOUR OWN WORDS. DO NOT USE QUOTATIONS. If you have any questions, please discuss with your TA/Instructor **before** turning in your report.

Take a look at the relevant sections of the grading rubric for the final full report (at the end of this document). The rough draft will be graded using similar criteria.

Here is the grading breakdown for the draft:

- Complete and accurate content at time of draft (15pt)
- Writing and mechanics: clarity, logical flow of information, spelling/grammar (5pt)
- Support and sufficient references (5pt)

PCB3063L Lab Report 2: Full Lab Report (70pts)

You will be writing a full lab report based on the Molecular Genetics project investigating mutations in the fly genome. It will include a **Title Page, Introduction, Materials & Methods, Results, Discussion, and Reference section**. The total length of your paper should be 8-10 pages of text. **Please do not exceed 12 pages (not counting the title page, figures/tables & legends, and reference page).** In addition to the details provided below, follow the general guidelines for lab reports and review the syllabus for Turnitin and plagiarism policies.

For this lab report, you will also be assessed on your ability to properly address general issues or suggestions from Instructor/TA edits/feedback on the previous report and draft. See the grading rubric for additional details and point distributions for this report.

5) **Title page**: The title of the paper, your name, your group members' names, the date, and the course title with section number should be included.

6) **Introduction**: Introduce the research question and purpose of experiments performed, providing sufficient background for the reader to understand the project, including the major biological principles being examined. Once defining the research question your experiments address, provide ***an overview*** of the experimental model and approach (techniques) utilized. State the hypothesis, providing rationale (reason/justification) for the hypothesis based on pertinent observations and information already known from the literature. State the anticipated results given your hypothesis and discuss the rationale for expecting this outcome.
   Your goal is to clearly and concisely write this information in ~2 pages. Take a look at some of the research articles you are using for background information to see examples of types and flow of information typical in an introduction.
   Any information from other sources, including any figures you did not generate yourself, must be properly cited in-text/below the figure AND in the reference section.

7) **Materials & Methods:** Concisely describe the procedures you carried out to investigate DNA mutations in the fly genome. It should be a brief synopsis of what you did in your own words in the past tense, including necessary details for someone else to be able to repeat the experiment themselves. Include anything that you did differently from that presented in the lab manual. You should break up this section into different sub-sections with subheadings, including a sequence analysis section in addition to the hands-on laboratory procedures.
   For any procedural steps your group/lab section did not perform directly, you may note the samples and basic procedures that were performed for you as "described previously (MBS, year)", citing the lab manual.
   For example: Inoculation of mini prep cultures from colonies was performed as described previously (MBS, 2023).
   Look at research articles you have found for examples of methods/materials style research paper writing. It is detailed, but very concise, in short paragraph form. **The lab manual will be**

cited in-text here, and writing must be paraphrased, in your own words, not copied directly from the manual.

Just for this section only, you may choose to include a brief statement at the start of the materials and methods to note that the following procedures were adapted from the MBS lab manual (MBS, year) instead of providing in-text citations throughout the materials and methods sections.

8) **Results**: Concisely state the results you obtained at each experimental step where observations, measurements, and/or analyses were done. Be sure to include the following: all gel images, calculations and observations, DNA concentrations/purity results, and DNA sequencing data analysis. Remember this section should be written in paragraph form with tables/figures and calculations to support the text. Figures and written text legends are in addition to Results paragraph text writing and should be detailed enough to stand alone.

9) **Discussion**: In this section, you will discuss the results/data you obtained and interpret their meaning. Were you able to draw any definitive conclusions from your analysis? Discuss how the results may or may not support your hypothesis and why. Also include discussion of how any controls utilized may or may not have supported/given you confidence in your results. Identify potential sources of error or any experimental issues that arose and suggest how these may have impacted the results. Are there any changes or improvements you would make if you were to repeat any of these experiments and why/future directions to consider? In this section, you will want to discuss the results/data your group/class obtained.

   You will **need to cite** within this section as you do with all other sections of your report. Look at other research articles you have found for additional examples of information to include in this section of your paper. The Discussion section is typically ~2 pages.

10) **References**: You should include any items you used to write your report, including the lab manual. You should use at *least* 4 **scientific** sources, with **at least two of your sources being journal articles**. Carefully follow the formatting guidelines for in-text citations and reference section listings.

   Websites are typically not appropriate sources.

Important Notes: Upload your report to Turnitin by the deadline to avoid late penalties. Your lab report will not be graded unless uploaded to Turnitin through Canvas. You MUST include citations throughout your text in the report and all writing must be in your own words. DO NOT COPY what is written in the lab manual, textbook, online resources, etc. Working together with group members, sharing of data, and discussion of results are permitted, but your report MUST BE IN YOUR OWN WORDS. DO NOT USE QUOTATIONS. If you have any questions, please discuss with your TA/Instructor **before** turning in your report.

Grading Rubric: Genetics Lab PCB 3063L Lab Report 2

Points in between values below are possible when appropriate.

	3 points	2 points	1 points	0 points
**Title Page**	All required information is present. The title is complete, accurate, and descriptive of the work.	All required information is present. The title is imprecise, but relevant to the work.	Most required information is present. The title is imprecise or not entirely relevant to the work.	Title page is missing.

	12 points	8 points	4 points	0 points
**Introduction**	Clear understanding of the purpose of the experiments done. Discussion of research question, experimental approach, and relevant background is complete and accurate. Hypothesis and expected results are accurately and fully represented.	The experimental purpose and hypothesis are included, though not entirely clear. Discussion of research question and experimental approach is included, but is not entirely accurate or background information is incomplete. Expected results include inaccuracies or are incomplete.	The content is lacking a full description of the purpose and hypothesis for the experiments. Discussion of research question and experimental approach is incomplete and some description is not accurate. Expected results are missing.	There is no clear understanding of the purpose and hypothesis for the experiments. The techniques utilized and expected results are not discussed.

Grading Rubric: Genetics Lab PCB 3063L Lab Report 2

	12 points	8 points	4 points	0 points
**Materials and Methods**	Experimental methods are clear, concise, and accurate with sufficient details provided. Methods are divided into sections with accurate and relevant subheadings. Writing reflects what was done in the lab.	Experimental methods are mostly clear, concise, and accurate. However, there are one or two pieces of information that are not clear or accurate. A few details are missing to be able to fully understand the experimental steps taken. Methods are divided into sections with accurate and relevant subheadings. Writing reflects what was done in the lab.	Experimental methods are unclear or too wordy, containing several errors or omissions. Methods are not properly divided into sections or subheadings are not accurate/relevant. Writing may reflect what is outlined in the manual, but does not include any updates or details specific to the student's own experience in the lab.	Experimental methods are unclear and inaccurate with many important details omitted such that it is difficult to follow what was done. Sections with subheadings are not utilized or are not clear nor relevant.

	12 points	8 points	4 points	0 points
**Results**	Experimental results are clear, concise, and accurate. Results are written in paragraph form and well supported by figures/tables and all required data, including gel images, observations, calculations, and analyses from the work completed. Writing and data provided are complete and reflect what was done in the lab.	Experimental results are mostly clear, concise, and accurate. However, there is a result that is unclear, inaccurate, and/or missing. Proper format is utilized, and all required tables/figures and data are included. However, there is an occasional error or mislabel present. Writing reflects what was done in the lab.	Experimental results are unclear or incomplete, containing several errors and/or omissions in the writing, tables/figures, or calculations. Results are not written in paragraph form. Writing does not fully reflect all that was done in the lab.	Experimental results are unclear and inaccurate with many important details and data omitted such that it is difficult to follow what was done. Tables/figures contain many errors and/or are inappropriate. Appropriate formatting is not followed.

Grading Rubric: Genetics Lab PCB 3063L Lab Report 2

	12 points	8 points	4 points	0 points
**Discussion**	Accurate, clear interpretation of all data obtained and conclusions drawn, supported by any controls utilized. If/how results support your hypothesis is evaluated. Potential sources of error/experimental issues are discussed, including suggestions for if/how these may have impacted the results and could be improved upon if experiments were repeated.	Discussion is mostly clear and accurate, but one piece of information is incorrect or misinterpreted. All data and conclusions drawn are discussed. Conclusions drawn are supported by any experimental controls utilized. How results relate to the hypothesis is not entirely clear. Potential sources of error/experimental issues that occurred are mentioned, but not clearly discussed.	The discussion of data and conclusions drawn is not entirely clear and contains a few omissions. There are a few errors in the interpretation of the gene analyzed or controls used. How results relate back to the hypothesis is unclear and potential sources of error/experimental issues that arose are not mentioned.	The discussion of data and conclusions drawn is not clear and discussion of several pieces of data is missing. Interpretation of controls, how results relate back to the hypothesis, and potential sources of error are missing.

	10 points	7 points	4 points	0 points
**Writing and Mechanics**	Writing is clear, presented in a logical order, and easy to follow. The text is written with no errors in grammar, capitalization, punctuation, and spelling. Tables and figures are complete, are correctly labeled, include relevant and complete legends, and are fully described in the text. Instructor/TA edits/suggestions from previous lab reports are all addressed.	Writing is clear, but order of information makes it more difficult to follow the logic presented. Text is written with several errors in grammar, capitalization, punctuation, and spelling. Tables and figures are labeled, but several labels are inaccurate or text description and/or legend is inaccurate or incomplete in places. Most instructor/TA edits/suggestions from previous lab reports are addressed.	Information is not presented in a logical order. Spelling, punctuation, and grammar errors distract or impair readability. Some tables and figures are missing labels/legends or have many inaccurate labels. Text description of tables and figures is not clear. Some instructor/TA edits/suggestions from previous lab reports are addressed.	Writing is not clear and therefore difficult to follow. Errors in spelling, capitalization, punctuation, usage and grammar repeatedly distract the reader and major editing and revision is required. Labels, legends, and text description for figures/tables is missing. Instructor/TA edits/suggestions from previous lab reports are not addressed.

Grading Rubric: Genetics Lab PCB 3063L Lab Report 2

	9 points	6 points	3 points	0 points
**References**	At least 4 scientific sources are used and are relevant, current, credible, and accurately interpreted. In-text and reference section citations are properly formatted.	At least 4 scientific sources are used, but one of the sources is inappropriate or misinterpreted. There are one or two errors in the in-text or reference section formatting.	An insufficient number of relevant, credible, and current sources is utilized. There are places where additional references could be useful. There are multiple errors in the in-text or reference section formatting.	No references other than the lab manual are utilized or other references used are inappropriate. Formatting guidelines are not properly followed.

70 points total

For all written assignments, be sure to **WRITE IN YOUR OWN WORDS AND APPROPRIATELY REFERENCE ALL SOURCES UTILIZED** (using in-text citations and listing references on the reference page). *Text quotations are not utilized in scientific writing*. Writing about someone else's work/ideas, even in your own words, but failing to cite the source is considered plagiarism. Please refer to the syllabus and USF's academic policies for further details on plagiarism policies in this course. If any of these points are unclear, please discuss with your TA before turning in your report.
