# Supplementary material for: Identifying DNA mutations in Sepia, White, Yellow, and Vestigial genes in D. melanogaster - Integrating inquiry-based experiential learning into undergraduate Genetics Laboratory curriculum
Source: PLoS One. 2026 Sep 1;21(9):e0356998. doi: 10.1371/journal.pone.0356998 (PMC13533379; doi:10.1371/journal.pone.0356998)
Supplement: Supplementary file 5 [file pone.0356998.s006.pdf]

# PCB3063L Fall 2023 Laboratory Schedule

| Week of | Exercise | What's Due |
|---|---|---|
| | | |
| **8/22**<br>**Week 1** | Course Intro and Safety Guidelines<br>Introduction to *Drosophila* | *Bring safety goggles* |
| | | |
| **8/29**<br>**Week 2** | Quiz #1<br>Determination of an Unknown Genotype<br>Experimental design activity<br>Review Lab Report I guidelines | **Labster Mendelian Sim**<br>*Bring safety goggles* |
| | | |
| **9/5**<br>**Week 3** | Quiz #2<br>Determination of an Unknown Genotype, continued<br>Introduction to Mol. Bio. Techniques<br>Pipetting skill check activity | *Bring safety goggles* |
| | | |
| **9/12**<br>**Week 4** | Determination of an Unknown Genotype, continued<br>Investigation of Mendelian Patterns of Inheritance<br>Research Question and Experimental Design Overview<br>Genomic DNA Extraction<br>Hypothesis Draft 1<br>Citation activity | **Labster PCR Sim**<br>*Bring safety goggles* |
| | | |
| **9/19**<br>**Week 5** | Quiz #3<br>Genomic DNA Extraction, continued<br>Amplification of genomic regions of interest by PCR<br>Data analysis activity #1<br>Hypothesis Draft 2 | **Lab Report 1**<br>**Notebook Check 1**<br>*Bring safety goggles* |
| | | |
| **9/26**<br>**Week 6** | **Midterm**<br>PCR, continued<br>Hypothesis Draft 3<br>Review Lab Report 2 guidelines | *Bring safety goggles* |
| | | |
| **10/3**<br>**Week 7** | Quiz #4<br>PCR, continued<br>Ligation of PCR products and bacterial transformation | **Lab Report 2: Rough Draft**<br>*Bring safety goggles* |
| | | |
| **10/10**<br>**Week 8** | Quiz #5<br>Ligation and Transformation, continued<br>Miniprep of plasmid DNA<br>Quantification of plasmid DNA<br>Restriction analysis of plasmid DNA | *Bring safety goggles* |
| | | |
| **10/17**<br>**Week 9** | Quiz #6<br>Restriction analysis, continued<br>Data analysis activity #2<br>DNA Sequencing of plasmid DNA<br>Intro to genome databases and computational analysis | *Bring safety goggles* |
| | | |

| | | |
|---|---|---|
| **10/24**<br>**Week 10** | Sequencing analysis cancer case study<br>Data analysis activity #3<br>Rough draft feedback and Report 2 guideline reminders | **Notebook Check 2**<br>*Bring laptop where possible* |
| | | |
| **10/31**<br>**Week 11** | Review of Molecular Experimental Design<br>DNA Sequencing Analysis<br>Data analysis activity #4 | *Bring laptop where possible* |
| | | |
| **11/7** | *No labs – Veterans Day Holiday* | |
| | | |
| **11/14**<br>**Week 12** | **Comprehensive Final Exam** | **Lab Report 2: Full Report** |
| | | |
| **11/21** | *No labs – Thanksgiving Holiday* | |
| | | |
| **11/28** | *No labs – Test Free Week* | |
| | | |

# PCB3063L Spring 2024 Laboratory Schedule

| Week of | Exercise | What's Due |
|---|---|---|
| | | |
| 1/8<br>Week 1 | Course Intro and Safety Guidelines<br>Introduction to *Drosophila* | *Bring safety goggles* |
| | | |
| 1/15 | *No labs – Dr. Martin Luther King, Jr. holiday* | |
| | | |
| 1/22<br>Week 2 | Quiz #1<br>Determination of an Unknown Genotype<br>Experimental design activity<br>Review Lab Report I guidelines | **Labster Mendelian Sim**<br>*Bring safety goggles* |
| | | |
| 1/29<br>Week 3 | Quiz #2<br>Determination of an Unknown Genotype, continued<br>Introduction to Mol. Bio. Techniques<br>Pipetting skill check activity | *Bring safety goggles* |
| | | |
| 2/5<br>Week 4 | Determination of an Unknown Genotype, continued<br>Investigation of Mendelian Patterns of Inheritance<br>Research Question and Experimental Design Overview<br>Genomic DNA Extraction<br>Hypothesis Draft 1<br>Citation activity | **Labster PCR Sim**<br>*Bring safety goggles* |
| | | |
| 2/12<br>Week 5 | Quiz #3<br>Genomic DNA Extraction, continued<br>Amplification of genomic regions of interest by PCR<br>Data analysis activity #1<br>Hypothesis Draft 2 | **Lab Report 1**<br>**Notebook Check 1**<br>*Bring safety goggles* |
| | | |
| 2/19<br>Week 6 | **Midterm**<br>PCR, continued<br>Hypothesis Draft 3<br>Review Lab Report 2 guidelines | *Bring safety goggles* |
| | | |
| 2/26<br>Week 7 | Quiz #4<br>PCR, continued<br>Ligation of PCR products and bacterial transformation | **Lab Report 2: Rough Draft**<br>*Bring safety goggles* |
| | | |
| 3/4<br>Week 8 | Quiz #5<br>Ligation and Transformation, continued<br>Miniprep of plasmid DNA<br>Quantification of plasmid DNA | *Bring safety goggles* |

| | | |
|---|---|---|
| | Restriction analysis of plasmid DNA | |
| | | |
| 3/11 | *No labs – Spring Break* | |
| | | |
| 3/18<br>Week 9 | Quiz #6<br>Restriction analysis, continued<br>Data analysis activity #2<br>DNA Sequencing of plasmid DNA<br>Intro to genome databases and computational analysis | *Bring safety goggles* |
| | | |
| 3/25<br>Week 10 | Sequencing analysis cancer case study<br>Data analysis activity #3<br>Rough draft feedback and Report 2 guideline reminders | **Notebook Check 2**<br>*Bring laptop where possible* |
| | | |
| 4/1<br>Week 11 | Review of Molecular Experimental Design<br>DNA Sequencing Analysis<br>Data analysis activity #4 | *Bring laptop where possible* |
| | | |
| 4/8<br>Week 12 | *No lab meeting – Complete & Submit Final Lab Report* | **Lab Report 2: Full Report** |
| | | |
| 4/15<br>Week 13 | **Comprehensive Final Exam** | |
| | | |
| 4/22 | *No labs – Test Free Week* | |
| | | |

# PCB3063L Summer 2024 Laboratory Schedule

| Week of | Exercise | What's Due |
|---|---|---|
| | | |
| **5/14**<br>**Week 1** | Course Intro and Safety Guidelines<br>Introduction to *Drosophila* | *Bring safety goggles* |
| | | |
| **5/21**<br>**Week 2** | Quiz #1<br>Determination of an Unknown Genotype<br>Experimental design activity<br>Review Lab Report I guidelines | **Labster Mendelian Sim**<br>*Bring safety goggles* |
| | | |
| **5/28**<br>**Week 3** | Quiz #2<br>Determination of an Unknown Genotype, continued<br>Introduction to Mol. Bio. Techniques<br>Pipetting skill check activity | *Bring safety goggles* |
| | | |
| **6/4**<br>**Week 4** | Determination of an Unknown Genotype, continued<br>Investigation of Mendelian Patterns of Inheritance<br>Research Question and Experimental Design Overview<br>Genomic DNA Extraction<br>Hypothesis Draft 1<br>Citation activity | **Labster PCR Sim**<br>*Bring safety goggles* |
| | | |
| **6/11**<br>**Week 5** | Quiz #3<br>Genomic DNA Extraction, continued<br>Amplification of genomic regions of interest by PCR<br>Data analysis activity #1<br>Hypothesis Draft 2 | **Lab Report 1**<br>**Notebook Check 1**<br>*Bring safety goggles* |
| | | |
| **6/18**<br>**Week 6** | **Midterm**<br>PCR, continued<br>Ligation of PCR products and bacterial transformation<br>Hypothesis Draft 3<br>Review Lab Report 2 guidelines | *Bring safety goggles* |
| | | |
| **6/25**<br>**Week 7** | Quiz #4<br>PCR, continued<br>Miniprep of plasmid DNA<br>Quantification of plasmid DNA<br>Restriction analysis of plasmid DNA | **Lab Report 2: Rough Draft**<br>*Bring safety goggles* |
| | | |
| **7/2**<br>**Week 8** | Restriction analysis, continued<br>Data analysis activity #2<br>DNA Sequencing of plasmid DNA<br>Intro to genome databases and computational analysis<br>Sequencing analysis cancer case study<br>Data analysis activity #3 | **Notebook Check 2**<br>*Bring laptop where possible* |
| | | |

| | | |
|---|---|---|
| **7/9**<br>**Week 9** | Quiz #5<br>Review of Molecular Experimental Design<br>DNA Sequencing Analysis<br>Data analysis activity #4<br>Rough draft feedback and Report 2 guideline reminders | *Bring laptop where possible* |
| | | |
| **7/16**<br>**Week 10** | **Comprehensive Final Exam** | **Lab Report 2: Full Report** |
| | | |
