# Supplementary material for: Identifying DNA mutations in Sepia, White, Yellow, and Vestigial genes in D. melanogaster - Integrating inquiry-based experiential learning into undergraduate Genetics Laboratory curriculum
Source: PLoS One. 2026 Sep 1;21(9):e0356998. doi: 10.1371/journal.pone.0356998 (PMC13533379; doi:10.1371/journal.pone.0356998)
Supplement: Supplementary file 6 [file pone.0356998.s007.pdf]

Genetics Laboratory Manual
PCB 3063L

Mendelian and Molecular Genetics: Investigation of Wing Development and Pigment Biosynthesis Pathways in *Drosophila melanogaster*

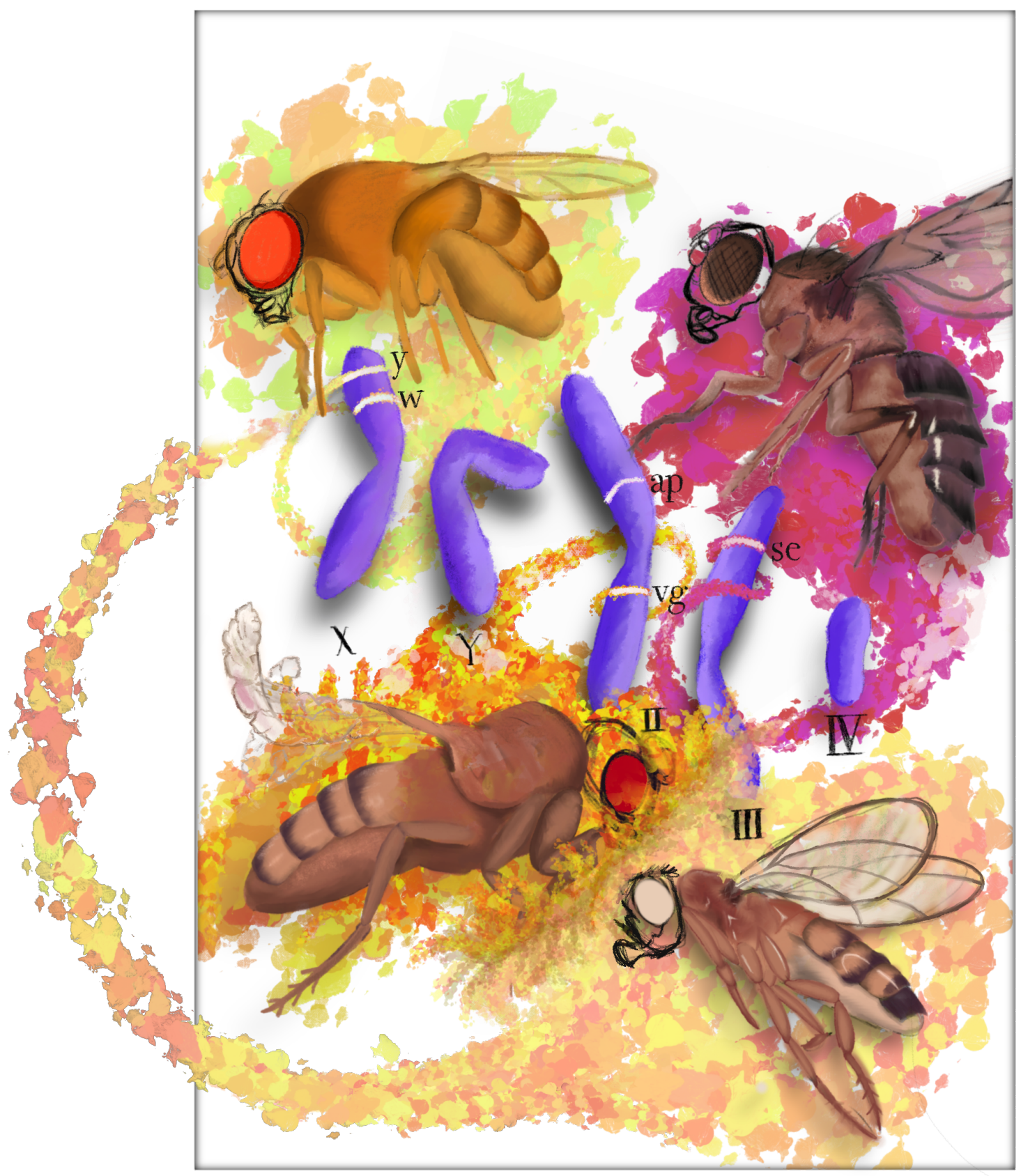

Department of Molecular Biosciences
University of South Florida, Tampa

Fall 2024

Name:______________________________ Section #:____________
