# Supplementary material for: Identifying DNA mutations in Sepia, White, Yellow, and Vestigial genes in D. melanogaster - Integrating inquiry-based experiential learning into undergraduate Genetics Laboratory curriculum
Source: PLoS One. 2026 Sep 1;21(9):e0356998. doi: 10.1371/journal.pone.0356998 (PMC13533379; doi:10.1371/journal.pone.0356998)
Supplement: Supplementary file 7 [file pone.0356998.s008.pdf]

PCB 3063L: General Genetics Laboratory

Syllabus – Fall 2023

Department of Molecular Biosciences
University of South Florida, Tampa

Course Supervisor

Krishna Rentachintala krishnar@usf.edu

**When e-mailing, please include your course and section number.

Lab Times

Section	CRN	Day	Time	TA/Instructor	TA/Instructor email
003	82138	Tuesday	8:35am-11:25am		
004	82145	Tuesday	11:50am-2:40pm		
005	82157	Tuesday	3:05pm-5:55pm		
902	82181	Tuesday	6:05pm-8:55pm		
006	82166	Wednesday	9:30am-12:20pm		
007	82168	Wednesday	12:30pm-3:20pm		
008	82170	Wednesday	3:30pm-6:20pm		
903	82182	Wednesday	6:30pm-09:20pm		
009	82171	Thursday	8:35am-11:25am		
010	82172	Thursday	11:50am-2:40pm		
011	82173	Thursday	3:05pm-5:55pm		
904	82183	Thursday	6:05pm-8:55pm		
012	82174	Friday	8:35am-11:25am		
013	82175	Friday	11:50am-2:40pm		

- PCB3063L is a 1 credit hour laboratory course. Students must have completed or be currently enrolled in the General Genetics lecture course (PCB 3063).
- **All sections of this course meet in person, in the ISA 3023 Genetics lab.**
- TA/Instructor office hours will be held in BSF 221 when meeting in person or held remotely through Microsoft Teams. Your TA/Instructor's office hours will be announced during the first week of class.

Materials Required

- Laboratory coat
- Safety goggles (wraparound or classic style)
- **Required Text**: Fall 2023 Genetics Lab Manual, MBS Department, USF printed by Pro-Copy. The lab notebook is bundled with the lab manual and is available for purchase at the USF bookstore. ***Please have your Fall 2023 edition lab manual and notebook no later than week 2 of class,*** **preferably by the first week. We will be using the manual and notebook the first week of class.**
- Computer and Internet Access

  - Links to and integration of software, videos, and tools used in the lab course will be provided through Canvas. These include, but are not limited to, Labster, JoVE, NEB Cutter, NCBI BLAST, T-COFFEE, and Expasy Translate.

Student Learning Outcomes

- Utilize Mendelian and molecular genetics principles, along with the current literature, to form hypotheses, design and perform experiments, and interpret results in the lab
- Read and reference scientific research articles
- Discuss examples of model organisms, techniques, and equipment utilized in genetics research
- Collect, analyze, and present experimental data in a laboratory notebook and scientific reports
- Determine if data support the hypothesis and suggest potential experimental sources of error
- Predict the outcome of genetic crosses and determine genotypes and allele frequencies
- Perform statistical analysis to evaluate data sets
- Amplify and sequence genomic regions of interest to investigate DNA mutations responsible for mutant phenotypes in the fly
- Use online resources and databases to analyze sequencing data, locate restriction enzyme cutting sites, and predict how DNA mutations may impact protein function
- Generate predicted mRNA/cDNA and protein sequences from genomic DNA sequences
- Connect genotype to phenotype through Mendelian and molecular analysis
- Identify real-world applications for the laboratory techniques and analyses covered, for example in the fields of medicine and pharmacology

General Course Description

Laboratory investigation techniques in general genetics including Mendelian and non-Mendelian relationships, and gene interactions.

Class Format

This genetics lab course will cover both Mendelian inheritance and molecular genetics through the use of guided inquiry laboratory activities and a hypothesis-driven guided research project using *Drosophila melanogaster* as our research model organism. We will review the concepts, techniques, and analyses of each lab exercise and then work together in groups to carry out the experiments. Our goal is to support an inclusive and equitable learning environment that values diversity of all classroom community members.

Strategies for Success

- Read each lab exercise and complete any pre-lab assignments before coming to class so that you will be prepared when arriving to lab each week.
- Review the previous lab exercises before class so that you will be prepared to fully participate in class discussions and group activities.
- Take notes, record data in your lab notebook, and note any updates to the lab manual procedures specific to your lab section’s experiments so you have the details needed to complete your lab reports and to review content for quizzes and exams.

- Lab reports are long-term assignments that you should work on week by week as you complete relevant procedures and data analysis. They are a significant grade component in this course. Work in advance of deadlines to avoid late penalties, ensure timely receipt of feedback, and to make sure completion of work due at the start of class does not risk a late arrival.
- Save and backup work frequently. Please also check that you have successfully uploaded the correct file by the deadline for each assignment submission.
- Take advantage of your TA/Instructor office hours to review performance on graded assessments and ask any remaining questions you have relevant to the lab exercises.

Attendance Policy

The following is a Cell Biology, Microbiology, and Molecular Biology Departmental laboratory course attendance policy.

Lab attendance is **mandatory** and arrival to lab is expected to be **on time**. Laboratory exercises build upon each other, so missing a lab can cause learning gaps and students to fall behind. Students who miss a laboratory without a documented, valid excuse will lose **5% of the possible total score for the first occurrence and 10% of the possible total score for each subsequent occurrence**. Due to the hands-on nature of laboratory courses, *more than 3 absences, whether excused or unexcused, result in a failing grade (F) for this course.* If you find that you are absent for several classes due to illness or other circumstances, it is recommended that you reach out to the lab supervisor and determine if dropping the class should be considered.

Absences will be excused for documented, legitimate reasons according to university guidelines (unforeseen: e.g. medical emergency, death in the immediate family; and special circumstances: e.g. jury duty, day of religious observance, USF-sponsored academic conference participation). Documentation may be a medical doctor's note, hospital admission papers, legal or a funeral notice that documents the specific date & time of the missed lab period including the student's name. Lab absences due to work obligations or inclement weather, unless classes are canceled as a result of university closure, do not represent excusable circumstances.

Please notify the TA/Instructor **at the beginning of the semester (by the second week of class) for foreseen excusable absences** (special circumstances) and **within 48 hours of the missed lab period for unforeseen excusable absences** to make arrangements for turning in any in-person assignments due/completed during the missed lab period. The proper supporting **documentation** for an excused absence should be presented to the TA/instructor **within one week** of the absence. Where possible, you may only attend a different lab section **with the specific direction** from the lab supervisor or TA/Instructor. *Laboratories cannot be made up beyond the week that a particular lab is scheduled, and it is not guaranteed that there will be space available to schedule attendance in another lab section.* Only documented, excused absences are considered for rescheduling.

Students who anticipate the necessity of being absent from class due to a major religious observance are asked to provide notice of the date(s) to their TA/Instructor, in writing, by the second week of classes. If an exact date for a major religious observance is not known in advance, the student should instead provide notice of the anticipated week of the religious observance by the second week of classes.

Late arrival or early departure is disruptive to the class, group work, and can often result in students missing important safety information and assignments. **Late arrival up to 15 minutes** after the lab start time is permitted, but will count against the student's participation points. If quizzes are given at the beginning of class, late arrivals will be given any remaining time to complete the quiz. If students arrive after or as quizzes are being collected, the student will receive a 0 for that quiz. **Students leaving early, within 15 minutes of completion of lab activities** will lose participation points for that day.

Please do not come to class if you are more than 15 minutes late*. This will count as an unexcused absence because it is disruptive to your TA/Instructor and classmates to arrive during lab activities and too much will have been missed to easily catch up.* If you encounter an unforeseen circumstance and are going to be late, please notify the TA/Instructor as soon as possible. **You unfortunately will not be permitted to stay** unless you have obtained prior permission from your TA/Instructor for an excusable, documented circumstance. **Students leaving more than 15 minutes prior to completion of the lab activities** without prior permission for an excusable, documented circumstance will also receive the point penalty for an unexcused absence due to too much work being missed.

Laboratory Grade

Component	Percent of Final Grade
Lab Participation (11)	10%
Labster Virtual Pre-Lab Simulations (2)	4%
Lab Notebook Check Assignments (2)	6%
Activities and Skill Checks (7)	14%
Quizzes (6)	12%
Lab Report Assignments (3)	29%
Exams (2)	25%
	**100%**

All posted grades are final unless they are the result of a miscalculation**.** If a student wishes to question a grade, they are asked to first speak with the TA/Instructor within 1 week of receiving the grade. For problems/questions that cannot be resolved through the TA/Instructor, the student may then contact the course supervisor by e-mail within 2 weeks of receiving the grade. When contacting the course supervisor, please provide all pertinent information: your name, course, section, and details of the grade issue including the assignment, specific assignment question #/graded component, and rationale for the specific dispute.

Grading Scale

Letter Grade	& %
A+	96.5 - 100
A	89.5 – 96.49
B+	86.5 – 89.49
B	79.5 – 86.49
C+	76.5 – 79.49
C	69.5 – 76.49
D+	66.5 – 69.49
D	59.5 – 66.49

F 0 – 59.49

Late drops, "S/U" and "I" grades will not be granted except under those circumstances specified in the USF undergraduate catalogue and College of Arts and Sciences policy. An "I" grade may only be considered under qualifying circumstances when a small portion of the student's work is incomplete and only when the student is otherwise earning a passing grade. There is no extra credit and no curve provided in this course.

Participation: Your TA/Instructor will evaluate your participation during each lab exercise. A total of 5 participation points is possible for each lab period during which you perform laboratory exercises. The average grade for all participation scores across the semester will contribute to **10% of your final grade in the course**. Factors determining participation grades include but are not limited to:

- Arriving on time
- Arriving prepared to the lab with all necessary supplies
- Proper conduct, staying on task, and keeping cell phones put away for the duration of lab
- Contributing fully to the work assigned and cooperation with other students and TA/Instructor
- Clean-up of your bench space and supplies

Students with an unexcused absence, as defined in the attendance policy above, will lose participation points for that day and points for any missed in-class graded component (eg. quiz, activity, notebook check), in addition to a point penalty, totaling a loss of 5% of the total course grade for the first unexcused absence and 10% of the total course grade for a second or third unexcused absence.

Students with a valid excusable absence with proper notification and documentation, who are unable to make up the lab exercises, will receive a participation grade for that week equal to their average participation grade for the remainder of the semester (for up to the three-absence limit).

Labster Virtual Pre-Lab Simulations: These simulations are **individual, untimed assignments** completed through the links provided in Canvas. **You must fully complete each simulation to receive credit**. You will receive unlimited attempts and the best score will be recorded. Simulation scores are based on on-time completion of the full case study and accuracy of answers to embedded theory questions. The Labster simulations are <u>not</u> covered in lab report assignments. These simulations will instead help you build background knowledge, reinforce key concepts, discover additional real-world applications, and provide familiarity with several hands-on techniques we use in the lab. The average grade for the 2 simulations assigned will contribute to **4% of your final grade in the course**, 2% each.

Lab Notebook: You will receive a Genetics lab notebook with your purchase of the lab manual. Data and procedures should be recorded and questions should be answered directly in this notebook. Keep any data analysis and figure production that you complete outside of lab in this lab notebook as well. Notebook checks are **individual assignments**. Your TA/Instructor will collect and grade your lab notebook during lab. Notebooks are due at the start of each class where notebook checks are assigned. The average grade for the 2 notebook checks will contribute to **6% of your final grade in the course**, 3% each. Notebook checks are graded on completeness, accuracy, and clarity.

Activities and Skill Checks: Activities and skill checks will be performed during lab to help you design experiments, perform basic techniques and calculations, determine expected results, and interpret and present data. In-class activities are completed **in groups** of up to 4 students. Some skill check activity

components are completed **individually**. Students missing an in-class activity/skill check with an excusable, documented absence may complete a makeup assignment within 1 week of the absence, during TA office hours or on their own, depending on the activity missed. Makeup assignments may be different in style and length than the version given in class. The average grade for the 7 activities and skill checks across the semester will contribute to **14% of your final grade in the course**, 2% each.

Quizzes: Quizzes are **individual** assessments held at the start of lab and consist of four to five questions on material covered during the previous week's lab as well as the material you were to prepare for that day. Each quiz is worth a total of 10pts, including but not limited to short answer, fill-in, or multiple-choice style questions. Electronic devices, including but not limited to laptops, tablets, smart watches, and cell phones, are not permitted during in-person quizzes. Use of a non-graphing, simple or scientific calculator is permitted. Students missing a quiz for an excused, documented absence may schedule to take a makeup quiz during their TA's office hours within 1 week of the absence. Makeup quizzes may be different in question style than version given in class. The average grade for the 6 quizzes across the semester will contribute to **12% of your final grade in the course**, 2% each.

Lab Reports: You will be writing and submitting a total of 3 lab report assignments to help you learn to present scientific data and write in the style of scientific literature in the field. Lab report assignments are **individual**, **one-time single file submission**, and will be submitted **electronically** in typed word doc format through Canvas, **Turnitin-enabled**. Reports are long-term assignments completed week by week leading up to each assignment deadline. Lab report 1 will focus on the Mendelian genetics experiments/statistical analyses and is a partial report (Methods & Materials, Results, Discussion, and References) worth 10% of the final course grade. Lab report 2 will focus on the molecular genetics research project and is a full lab report worth 14% of the final course grade. A rough draft, worth 5% of the final course grade, will be submitted for feedback prior to completing Lab report 2. The rough draft will include the Introduction and Materials & Methods completed thus far, with References. The feedback you receive from your TA/Instructor on Lab report 1, the Lab report 2 draft, and figures/legends included in Notebook check 2 will be helpful in providing guidance for writing the full Lab report 2. Specific directions for each report component, along with grading rubrics, will be provided by your TA/Instructor and on the Canvas course website. Together, these report assignments will contribute to **29% of your final grade in the course.**

Exams: A midterm and a final exam will be given during this course. These are **individual** assessments. The midterm will include questions on material covered prior to the exam, and the final exam will be a cumulative exam. Exams will include, but not be limited to, fill-in, multiple-choice, short answer, and essay style questions. Electronic devices, including but not limited to laptops, tablets, smart watches, and cell phones, are not permitted during in-person exams. Use of a non-graphing, simple or scientific calculator is permitted. TA/Instructors will retain all exam papers. Students are strongly encouraged to review exams with their TA/Instructor during office hours within 1 week of the grade posting to get feedback on any content missed. Students missing an exam with an excused, documented absence may schedule to take a makeup exam during their TA's office hours within 1 week of the absence. Makeup exams may be different in question style and length than the version given in-class. Together, these exams will contribute to **25% of your final grade in the course**, with the midterm worth 10% and the final worth 15% of the final grade.

Submitting Assignments and Late Work Policy

Quizzes, activities, and exams are completed in-class during the time designated. Labster pre-lab simulations, notebook checks, and lab report assignments are due by the **start of class time** during the weeks designated, unless specified otherwise.

While extensions are not provided on long-term assignments (e.g. lab reports), late submission of simulations and lab report assignments is permitted, albeit at a **10% per day late penalty** based on the total point value of the assignment (10% if submitted during the first 24hr after the deadline, 20% if submitted 24-48hr after the deadline, etc.). A late penalty exceeding the score earned will result in a zero on the assignment.

If you experience any difficulty loading a Labster simulation:

- Give it time, some simulations take ~10min to open
- Check that you are using Chrome or Firefox
- Close any other programs/browsers running on your computer
- Clear cache and cookies

Please reach out promptly to your TA/Instructor with a screen shot of the error and contact Labster support in advance of the assignment deadline for further help if these above troubleshooting efforts don't resolve the issue.

If you encounter a technical issue submitting a lab report word doc file through Canvas, please email the file to your TA/Instructor to note the time of completion, reach out to the IT help desk (https://www.usf.edu/it/services/client-support) to resolve the Canvas issue so that you can submit your work through Turnitin, and provide your TA/Instructor with the IT ticket number. It is helpful to screen shot and send images of errors received to help troubleshoot issues encountered. For this reason, please do not wait until the last minute to submit work so that you do not risk incurring a late penalty. Any file submitted by email before the deadline will only be graded without late penalty if it is the same file uploaded through Canvas to Turnitin once the technical issue is resolved.

Academic Integrity

Academic integrity is the foundation of the University of South Florida's commitment to the academic honesty and personal integrity of its university community. Academic integrity is grounded in certain fundamental values, which include honesty, respect, and fairness. Broadly defined, academic honesty is the completion of all academic endeavors and claims of scholarly knowledge as representative of one's own efforts. Academic dishonesty will not be tolerated. This includes, but is not limited to, cheating, plagiarism, fabrication, complicity, and multiple submissions, be it intentional or careless violations. The process for faculty reporting of academic misconduct, as well as the student's options for appeal, are outlined in detail in USF System Regulation 3.027.

While students may use digital communication tools to communicate with fellow students, it is important to remember that academic integrity policies still apply in these environments. Informing others about the contents of quizzes and tests is prohibited, as is receiving unauthorized information about an examination. Students are expected and required to immediately report instances of such violations to the instructor.

Plagiarism and Turnitin

During the semester, many lab activities will be completed in groups of up to 4. Discussing & working together with your group on in-class data analysis and group assignments is allowed, but individual makeup activities, simulations, quizzes, exams, notebook entries, and lab report materials, including figures and figure legends, must be completed by the individual student. Copying directly from another source (another student, the lab manual materials, the internet, journal articles, text books, or other sources), allowing another student access to copy your work, or using another student's work, from previous semesters or the current semester, is prohibited. When submitting lab report assignments, you must always **write in your own words AND reference the source of all information utilized.** References are to be provided both in-text and in the reference section of the report.

The use of generative AI tools for any course-related submissions is prohibited. This includes both direct use (e.g., submitting content generated by AI) and indirect use (e.g., using AI to generate ideas or outlines which are then built upon). Students are responsible for ensuring that all work submitted for this course is their own and does not involve the use of generative AI tools. The purpose of this policy is to encourage original thought, creativity, and to ensure that the learning outcomes for the course are achieved by the student themselves.

A copy of USF's Academic Plagiarism Policies is available in the Undergraduate Handbook. If you are unclear what plagiarism is or are not sure if you have plagiarized, please discuss with your TA/Instructor before turning in your report. Students found to have plagiarized or cheated will be penalized following University Policy. This includes and is not limited to: no credit for the assignment, failure in the course, or a grade of FF (a double FF denotes an F for Academic Dishonesty).

This course utilizes Turnitin. Turnitin is an anti-plagiarism program integrated into Canvas. You must upload all report files through Turnitin, which will then compare your work to a wide range of sources, including Internet sources, online text, the lab manual, and all other student papers submitted. A detailed originality report will be provided to your TA/Instructor showing any sources that have high degrees of similarity (i.e., have been plagiarized). Your assignment **will not be graded** unless it has been uploaded to Turnitin in Canvas. In order to comply with privacy laws, students are not required to include personal identifying information, such as name, in the body of the file submitted to Turnitin.

Multiple Submissions

Students who have previously taken this course are not permitted to turn in work from a previous semester. For example, the current semester report must be **completely** re-written using new material and figures reflecting the new methods/data/results from this semester. Students are not able to receive credit for work showing a high degree of similarity to work submitted in a previous semester.

Conduct in the course

Disruptive students in the academic setting hinder the educational process. Disruption of the academic process (USF Regulation 3.025) is defined as the act, words, or general conduct of a student in a classroom or other academic environment which in the reasonable estimation of the instructor: (a) directs attention away from the academic matters at hand, such as noisy distractions, persistent, disrespectful or abusive interruption of lecture, exam, academic discussion, or general University operations, or (b) presents a danger to the health, safety, or well-being of self or other persons.

The student will first receive a warning from the TA/Instructor. If the conduct is serious enough that the TA/Instructor feels the behavior endangers themselves and/or other students present, the TA/Instructor will have the right to ask the student to leave the lab, which will be considered an unexcused absence and could lead to dismissal from the course. Please refer to the following website for university polices regarding conduct and disruption of the academic process: http://regulationspolicies.usf.edu/ .

General Conduct and Communication Guidelines

- Act professionally in the way you communicate. Treat your instructors, TAs, and peers with respect. Respect other people's ideas and be constructive when explaining your views about points you may not agree with.
- Be sensitive. Be respectful and sensitive when sharing your ideas and opinions. There will be people in your class with different linguistic backgrounds, political and religious beliefs, and/or other cultural differences.
- Proofread and check spelling. Doing this before sending an email will allow you to make sure your message is clear and thoughtful.
- Keep your emails and postings related to the course content. You should not post anything personal on a discussion board, unless is requested by the TA/Instructor.
- Any personal, course or confidential issues should be directly communicated to the TA/Instructor/Lab supervisor via email.
- When sending emails to your TA/Instructor/Lab supervisor, please include your course and section number.

Laboratory Safety

Some of the reagents that are used in the lab are hazardous. Please follow all procedural and waste disposal instructions carefully. Those students who do not follow safety rules endanger themselves and their fellow students. Violations of safety rules can result in dismissal from the lab. If you are asked to leave, you will not receive credit for that day's session. Closed-toe shoes must be worn in the laboratory and feet and legs must be entirely covered. **No open-toed or open back shoes or sandals are permitted. Shorts and short skirts are not allowed.** Students should wear long pants or jeans, which provide better protection for the legs in case of a spill. Gloves must be worn at all times unless noted by the TA/Instructor. The fume hood must be used when appropriate under direction of your lab instructor. You must wear a lab coat at all times and wear safety goggles to protect yourself when working with corrosive or toxic chemicals and when there is any risk of splash or objects impacting the eye. No eating, drinking, or smoking is permitted in the lab at any time. No friends or children are permitted in the lab. Wash your hands after lab to prevent carrying any contamination home with you. If a student has any kind of mishap in the lab, an accident or near miss, the student is to immediately inform the TA/Instructor.

Laptop Usage

Laptop and tablet usage is not permitted at the lab bench during hands-on wet lab experiments due to risk of contamination with laboratory reagents. However, students are encouraged to bring laptops, as noted on the lab schedule, where possible during the two designated weeks when lab groups will be using computer programs to perform sequence analysis.

Use of Learning Assistants in the course

Where scheduling permits, lab sections may be assigned a peer learning assistant (LA) who will be present in the lab on designated days to facilitate in-class activities. If assigned, the LA will always be providing guidance with the TA/Instructor present. The LA will have no role in assigning grades, so any questions related to grades received must be directed to the TA/Instructor.

Lecture/Lab drop policy

When taken together with the Genetics lecture (PCB 3063), the lecture serves as a co-requisite for the lab. Withdrawal from the lecture component will therefore automatically drop you from the lab as well. However, a student may petition to withdraw from the lecture and remain in the lab. BioAdvise provides a petition form for this purpose (https://secure.cas.usf.edu/depts/bio/bioadvise/forms/co-req-petition.aspx). Once the form has been submitted and the permit has been issued, it is the student's responsibility to process the drop online before the withdrawal deadline. **Please be sure the proper permit is in place prior to dropping the lecture if you wish to stay in the lab.** Students may withdraw from the PCB 3063 lab and remain in the PCB 3063 lecture without need for a permit. Please email bioadvise@usf.edu if you are receiving an error that states you must drop both before proceeding with the drop.

Academic Accommodations

Students in need of academic accommodations may consult with the office of Students Accessibilities Services to arrange appropriate accommodations: www.usf.edu/SAS. SAS encourages students to notify instructors of accommodation needs at least five (5) business days prior to needing the accommodation. A letter from SAS must accompany this request. Please reach out to **the lab course supervisor** for all accommodation requests.

Academic Support and Wellness Services

The USF Office of Student Success coordinates and promotes university-wide efforts to enhance undergraduate and graduate student success. For a comprehensive list of academic support and wellness services available to all USF students, please visit the Office of Student Success website: http://www.usf.edu/student-success/ and the Student Affairs Wellness website: https://www.usf.edu/student-affairs/departments/wellness.aspx.

COVID-19 Policies

For the most recent communication regarding COVID-19 protocols, please visit:
https://www.usf.edu/coronavirus/**.** Students should not attend class if they are ill, particularly if they have fever and/or gastrointestinal symptoms and/or respiratory symptoms such as a sneezing, runny nose, sore throat or coughing. Students experiencing any of these symptoms should contact Student Health Services immediately (813-974-2331) for appropriate medical guidance and to obtain a *verification of care letter.*

Additional University Deadlines, Policies, and Procedures:

Withdrawal Deadline for the Fall 2023 semester: October 28, 2023

Policies about disability access, religious observances, academic grievances, academic integrity and misconduct, academic continuity, food insecurity, and sexual harassment are governed by a central set of policies that apply to all classes at USF. These may be accessed at: https://www.usf.edu/provost/faculty/core-syllabus-policy-statements.aspx

It is the student's responsibility to be aware of their academic standing and grade status. In an attempt to assist the student in evaluating his/her academic status mid-term, instructors will submit midterm grades electronically for each student.

All classes at USF make use of an online system for students to provide feedback to the University regarding the course. These surveys will be made available at the end of the semester, and the University will notify you by email when the response window opens. Your participation is highly encouraged and valued. The results of student feedback are sent to departments and faculty members after semester grades are already submitted, and student responses are reported only anonymously and in the aggregate to faculty.

USF considers the diversity of its students, faculty, and staff to be a strength and critical to its educational mission. USF expects every member of the university community to contribute to an inclusive and respectful culture for all in its classrooms, work environments, and at campus events. Dimensions of diversity can include sex, race, age, national origin, ethnicity, gender identity and expression, intellectual and physical ability, sexual orientation, income, faith and non-faith perspectives, socio-economic class, political ideology, education, primary language, family status, military experience, cognitive style, and communication style. The individual intersection of these experiences and characteristics must be valued in our community. Our goal is to help you be successful in a classroom where everyone feels safe and welcome.

USF is committed to providing an environment free from sex discrimination, including sexual harassment and sexual violence (USF Policy 0-004). The USF Center for Victim Advocacy is a confidential resource where you can talk about incidents of sexual harassment and gender-based crimes including sexual assault, stalking, and domestic/relationship violence. This confidential resource can help you without having to report your situation to the Title IX Office unless you request that they make a report. Contact the USF Center for Victim Advocacy: 813-974-5757, https://www.usf.edu/student-affairs/victim-advocacy/. Please be aware that in compliance with Title IX and under the USF Policy, educators must report incidents of sexual harassment and gender-based crimes including sexual assault, stalking, and domestic/relationship violence. **If you disclose any of these situations personally to an educator, they are required to report it to the Title IX Office**. For more information about Title IX, a full list of resources, or to report incidents of sexual harassment, sexual violence, relationship violence or stalking visit: www.usf.edu/title-ix/.

In the event of an emergency, it may be necessary for USF to suspend normal operations. During this time, USF may opt to continue delivery of instruction through methods that include but are not limited to: Canvas, Skype, and email messaging and/or an alternate schedule. **It's the responsibility of the student to monitor the Canvas course website for each class for course specific communication**, and

the main USF, College, and department websites, emails, and MoBull messages for important general information. For additional guidance on emergency protective actions and hazards that affect the University, please visit www.usf.edu/em

Finally, please note that we reserve the right to make changes to this syllabus, with notification to the students, during the course of the semester as situations may dictate.

PCB3063L Fall 2023 Laboratory Schedule

Week of	Exercise	What's Due
8/22 Week 1	Course Intro and Safety Guidelines<br>Introduction to *Drosophila*	*Bring safety goggles*
8/29 Week 2	Quiz #1<br>Determination of an Unknown Genotype<br>Experimental design activity<br>Review Lab Report I guidelines	**Labster Mendelian Sim**<br>*Bring safety goggles*
9/5 Week 3	Quiz #2<br>Determination of an Unknown Genotype, continued<br>Introduction to Mol. Bio. Techniques<br>Pipetting skill check activity	*Bring safety goggles*
9/12 Week 4	Determination of an Unknown Genotype, continued<br>Investigation of Mendelian Patterns of Inheritance<br>Research Question and Experimental Design Overview<br>Genomic DNA Extraction<br>Hypothesis Draft 1<br>Citation activity	**Labster PCR Sim**<br>*Bring safety goggles*
9/19 Week 5	Quiz #3<br>Genomic DNA Extraction, continued<br>Amplification of genomic regions of interest by PCR<br>Data analysis activity #1<br>Hypothesis Draft 2	**Lab Report 1**<br>**Notebook Check 1**<br>*Bring safety goggles*
9/26 Week 6	**Midterm**<br>PCR, continued<br>Hypothesis Draft 3<br>Review Lab Report 2 guidelines	*Bring safety goggles*
10/3 Week 7	Quiz #4<br>PCR, continued<br>Ligation of PCR products and bacterial transformation	**Lab Report 2: Rough Draft**<br>*Bring safety goggles*
10/10 Week 8	Quiz #5<br>Ligation and Transformation, continued<br>Miniprep of plasmid DNA<br>Quantification of plasmid DNA<br>Restriction analysis of plasmid DNA	*Bring safety goggles*
10/17 Week 9	Quiz #6<br>Restriction analysis, continued<br>Data analysis activity #2<br>DNA Sequencing of plasmid DNA<br>Intro to genome databases and computational analysis	*Bring safety goggles*

**10/24 Week 10**	Sequencing analysis cancer case study<br>Data analysis activity #3<br>Rough draft feedback and Report 2 guideline reminders	**Notebook Check 2**<br>*Bring laptop where possible*
**10/31 Week 11**	Review of Molecular Experimental Design<br>DNA Sequencing Analysis<br>Data analysis activity #4	*Bring laptop where possible*
**11/7**	*No labs – Veterans Day Holiday*	
**11/14 Week 12**	**Comprehensive Final Exam**	**Lab Report 2: Full Report**
**11/21**	*No labs – Thanksgiving Holiday*	
**11/28**	*No labs – Test Free Week*	

PCB 3063L Genetics Laboratory Policies:

Attendance in this class is Mandatory.

Students who anticipate the necessity of being absent from class due to a religious observance or other foreseen excusable absence are expected to provide notice of the date(s) to their TA/Instructor, in writing, by the second week of classes. Your instructor should be contacted within 48hr of an excused, unforeseeable absence and permission to attend another section must be sought prior to attendance in that laboratory and is not guaranteed. Excused absences (as described in the syllabus) must have supporting documentation.

Unexcused absences will result in a 5-10% reduction of the total possible grade in the course per occurrence as detailed in the syllabus**.** *More than 3 absences in the course, whether excused or unexcused, result in a failing grade (F).*

You may only attend the lab section that you are registered for unless specifically advised by your TA/Instructor.

Failure to come to class on time, prepared, and/or not following the safety rules will lead to lab participation points being deducted and/or dismissal from class, as outlined in the syllabus.

Assignments, including Labster simulations, lab report assignments, and notebook checks, are **due at the beginning of your lab section** unless specified otherwise**.** Any assignments submitted after the deadline will be subject to a **10% deduction in points for every day that they are late.**

Lab reports must be typed and conform to the format described in the guidelines sheet. **Lab reports must be submitted electronically to Turnitin through Canvas and are subject to plagiarism checking.**

I understand that plagiarism is a serious offense and if I am caught cheating/plagiarizing I will be penalized by receiving no credit for the assignment, failure in the course, or a FF.

The following are not permitted in the lab at any time: Eating/drinking, smoking, and cell phone use. Children are also not allowed in the lab at any time.

By signing below, you acknowledge that you understand the policies for this course and agree to abide by them:

Your signature______________________________________ Date______________

Your name (print) ____________________________________ Section #__________

STUDENT COPY: Please sign & return to your TA the duplicate copy provided to you. This copy serves as your record of the policies you are signing.

Teaching Laboratory Safety Guidelines

The following Safety Guidelines are to be strictly adhered to in all teaching laboratories. These rules apply to students, teaching assistants, and instructors. The Teaching Lab Manager must maintain records of all the signed documents.

- No food, drinks, smoking, chewing gum, or applying cosmetics and contact lenses in labs/studios/shops or clinics.
- Splash goggles are to be worn when there is a risk of chemical or biological material splashing. Eye protection needs to be worn when there is the possibility of an object impacting the eye.
- Footwear that covers the entire foot must be worn at all times. Therefore sandals, flip-flops, ballet-flats, backless and open-toed shoes are not acceptable.
- Clothing appropriate for laboratory safety must be worn. Clothing (pants or skirt) must be worn which completely covers the entire leg from the waist to the ankle. Clothing (shirt, blouse, etc.) must be worn which completely covers the torso from the waist to the neck. Shoulders must be completely covered and sleeves must be worn that cover the arm from the shoulder to at least halfway to the elbow. Therefore, tank tops, halters, shorts, cutoffs, etc. are not acceptable. Some lab courses may require the use of a lab coat/apron and/or gloves.
- Long hair should be tied back.
- Long, dangling jewelry, such as necklaces or earrings with the potential to interfere with or be contaminated by an experiment, should not be worn.
- Backpacks should be placed in the shelving units provided and not in the walkway.

Safety Suggestions

- Waste materials are to be disposed of immediately after use in the designated containers.
- All containers are to be labeled completely with full chemical names and a hazard description. For example: "6M Hydrochloric Acid: CORROSSIVE"
- Never leave an experiment unattended.
- Never leave a solution on a hot plate unattended.
- Hotplates that have been turned off, but are still hot, should have a warning note in front of them.
- Working alone is not recommended.

IF THERE IS A SERIOUS ACCIDENT, CALL 911 OR UNIVERSITY POLICE IMMEDIATELY. NOTIFY THE TEACHING LAB MANAGER.

I have read the safety guidelines listed above and understand that non-compliance will result in my dismissal from the laboratory until I do comply, and I will not be allowed to make-up missed work resulting from that dismissal.

Sign____ Course #________________

Print Name____ Section #________________

STUDENT COPY**:** Please sign & return to your TA the duplicate copy provided to you. This copy serves as your record of the policies you are signing.
