# Supplementary material for: Identifying DNA mutations in Sepia, White, Yellow, and Vestigial genes in D. melanogaster - Integrating inquiry-based experiential learning into undergraduate Genetics Laboratory curriculum
Source: PLoS One. 2026 Sep 1;21(9):e0356998. doi: 10.1371/journal.pone.0356998 (PMC13533379; doi:10.1371/journal.pone.0356998)
Supplement: Supplementary file 8 [file pone.0356998.s009.pdf]

Annotated *sepia* gene sequence

Drosophila melanogaster se FBgn0086348
Location: 3L:8,520,552-8,521,489

Exons

Protein coding

Primer set 1: Forward and Reverse Primer binding sites

Primer set 2: Forward and Reverse Primer binding sites

CTGTATCCACATGGCTTGGTATGTGTGTCAGCCGCAAGCCACCAAGTTCAGCCGCGTAAG

CGAAGCCAACCGGCGAAGGGCAGTGAGCTGCACTTTATAAAT**CACTCGAATGACAATCGC**

TATCACCACTTGCATCTCTGGACCCGAGCACGCACATCATGAGTAACGGCAGGCATTTGG

CAAAAGGTGGGTAGAGCCAGGAAACCAGGATAATCATAATTGATAATAAATGCGCCCACT

AG**GCTCACCCATGCCGGATGTTCCCGAAGATGGTATCCTTCGCCTGTACTCGATGCGCTT**

CTGCCCATTTGCCCAACGGGTGCATCTGGTCCTGGACGCCAAGCAGATCCCGTATCACAG

CATCTACATTAATCTCACAGACAAGCCGGAGTGGCTGCTGGAGAAGAATCCACAGGGCAA

GGTGCCGGCTCTAGAAATCGTGCGAGAACCTGGACCACCTGTGCTCACAGAGTCGCTACT

GATTTGTGAATATCTGGACGAGCAGTATCCATTGCGACCACTCTATCCACGTGATCCGCT

GAAGAAAGTGCAGGACAAGTTACTAATCGAGCGATTTAGAGCGGTGTTAGGTGCCTTCTT

CAAGGCATCCGATGGCGGTGATCTGGAGCCCTTCTGGAGCGGCCTGGACATCTACGAAAG

GGAGCTGGCTCGACGTGGTACGGAATTCTTTGGTGGCGAGCAGACGGGCATTCTGGACTA

TATGATTTGGCCCTGGTGTGAGCGCCTCGAGCTCCTTAAGTTGCAGCGTGGAGAGGATTA

TAACTACGATCAGAGTCGCTTCCCCCAGCTGGTGAGCAGGTCCGTGTATCCCAGGAGTGA

TGAAGTACATTAGTATACCATTTTTCAG**ACCCTTTGGCTGGAACGCATGAAACGAGATCC**

GGCTGTGATGGCCTTCTACATGGAGGCCGAGGTTCAGGCGGAGTTCCTGCGTACACGGAG

CCTGGGTCGACCCAATTACAATCTGCTGGTCAAGGATGCCTGATGCACTTGGTTTATTGA

ATTTTTCGCATGGAATGAATAAAACATAAATGCAGTCCATAACCGGTTCCAAAGATTTGA

TCTTGTCTAAACCAGTTGTCGTTCTATGGTGTACCCTTGAAGGCAATGTCGTACTGGGGA

TTTCCAAGGGTCTTCGATTTCTGGAACTCAGCATGCAACTGGACATCCAAAGCTGTCTTC

TGCACAACCTCATCCTGGGT**CATCAAATCGCGCCACTTGAG**CTGTAATGGATTTTTGATG
