# Supplementary material for: Identifying DNA mutations in Sepia, White, Yellow, and Vestigial genes in D. melanogaster - Integrating inquiry-based experiential learning into undergraduate Genetics Laboratory curriculum
Source: PLoS One. 2026 Sep 1;21(9):e0356998. doi: 10.1371/journal.pone.0356998 (PMC13533379; doi:10.1371/journal.pone.0356998)
Supplement: Supplementary file 9 [file pone.0356998.s010.pdf]

Annotated Yellow gene sequence

Drosophila melanogaster y FBgn0004034

Location: Chromosome X: 356,509-361,245 forward strand.

Exons

Protein Coding

Primer Set 1: Forward and Reverse Primer binding sites

Primer Set 2: Forward and Reverse Primer binding sites

Primer Set 3: Forward and Reverse Primer binding sites

Wildtype sequence:

ACCCGAATCACTAAAACCACCGAAGTTGGCGCGCCTTCGTTTTCATTTTCATTGGCCT

GTCTTCGTCTTCGGAGAAAAAAAACTTCATATAAAACGCGGCCGACATATTATGGCCACC

AGTCGTTACCGCGCCACGGTCCACAGAAGAGGATTAAAAAAATATCACACAGCCGAAGGC

TAGAGAAGAACCCCCTATAGCTGAACATATATAAACAAATATATTTTTTTTTATTGCCAA

CACACTTTGGCTTAAGTGTTAAGAGTGATTGTCAGCTTAGAGCTAAGTGCAATGTTCCAG

GACAAAGGGTGGATCCTTGTGACCCTGATCACCTTGGTGACGCCGTCTTGGGCTGCTTAC

AAACTTCAGGAGCGATATAGTTGGAGCCAGCTGGACTTTGCTTTCCCGAATACCCGACTA

AAGGACCAAGCTCTGGCTAGTGGAGATTATATTCCGCAAAATGCTCTACCTGTTGGAGTC

GAACACTTTGGCAATCGGTTATTCGTCACTGTTCCCCGCTGGCGTGATGGTAAGTGGAAG

TTAAATATGAAGCCCTTGGGGAGATCGTAAATGGGACATTCTTACTTAGGGCATCAGAGA

TATCTGATTGAGTGGTTGACAGTTTTATATGGCTTGTTTGACATGATGTAAAAACACAAA

ATTCATTTAGTTTAGGTATTCAAATAAGAGCTTGTTATTTATTTTAGAATTTGGAGAACA

TTTTTTTGTCTTTCTACCCTTCTTAGAAAATAATATTGTTTTGTACAATTTAATTTTAAA

CTAGTACAGACGAAAAATGTATTTTTTTATTTGTATGCCTTTTCACCATTTTGGCAGAGG

AATAAATATGACAATATATTTTGAGAGCACCCTCATGTAAAGGTTTTAGCGTGGCGACCT

CTCATAAATCCGGTTGGTACCTGCGCGTTATTTTAACATTTTAAACAATTAACCGTTGTA

AAATCGAAGCCAATAGCATGGCATTGGCTTTATACTGTATTAAATTGTATTATATTACCA

TCCGAATTGTAAAGACTTCTTCAGGGCCGCCACATAGAAATGGAAATCCAATCACAAACA

....

TACATATTATCCAGAAAACAGACAGCGATAACTTGCAACATTGCTTAGTATAATAATCCA

AAGAAGGAATTTAGGCAGAAATTCCAGTTAATTAAATATTCAAAACAAACTTTATTTAGT

GCCTCAATAATAGTTTGGCCCTGCTAATTCTCCTATTTTATTTTTTAG**GGATTCCGGCCA**

CTCTGACCTATATAAACATGGACCGCAGTTTGACGGGTTCACCGGAGCTAATTCCGTATC

CAGATTGGCGCTCAAATACAGCTGGAGATTGCGCCAACAGTATTACCACTGCCTACCGCA

TTAAAGTGGATGAGTGTGGTCGGCTGTGGGTTTTGGACACTGGAACCGTGGGCATCGGCA

ATACCACCACTAATCCGTGCCCCTATGCGGTAAATGTCTTTGACTTGACCACGGATACGC

GAATTCGGAGATACGAGCTACCTGGCGTGGACACAAATCCAAATACTTTCATAGCTAACA

TTGCCGTGGATATAGGCAAAAATTGCGATGATGCATATGCCTATTTTGCCGATGAATTGG

GATACGGCTTGATTGCTTACTCCTGGGAACTGAACAAGTCCTGGAGATTCTCGGCACATT

CGTATTTTTTCCCCGATCCATTGAGGGGCGATTTCAATGTCGCTGGTATTAACTTCCAAT

GGGGCGAGGAGGGTATATTTGGTATGTCCCTTTCGCCCATTCGATCGGATGGTTATCGTA

CCCTGTACTTTAGTCCGTTAGCAAGTCATCGACAATTTGCCGTATCCACGAGGATTTTGA

GGGATGAAACCAGGACGGAAGATAGCTATCATGACTTTGTTGCCTTAGATGAACGGGGTC

CAAACTCCCATACCACTTCACGTGTGATGAGCGATGATGGAATTGAGCTGTTCAATTTAA

TAGATCAAAATGCAGTGGGTTGCTGGCACTCATCAATGCCGTACTCACCGCAATTTCATG

GCATTGTGGATCGCGATGACGTTGGCTTAGTTTTTCCGGCCGATGTGAAAATTGATGAGA

ACAAAAACGTTTGGGTTCTATCCGATAGGATGCCCGTTTTCTTGCTGTCTGACTTGGATT

ATTCAGATACTAATTTCCGAATTTACACGGCTCCCTTGGCCACTTTAATTGAGAATACTG

TGTGTGATTTGAGGAATAACGCCTATGGGCCGCCAAATACCGTTTCAATACCAAAACAAG

CCGTTTTGCCAATGGGTCCACCGTTATATACGAAACAATATCGTCCTGTCTTGCCACAGA

AACCTCAGACCAGCTGGGCTTCCTCGCCGCCTCCTCCAAGTCGCACTTATTTGCCCGCCA

ATTCAGGCAATGTAGTCTCCAGTATTAGTGTCTCTACAAATTCTGTGGGTCCTGCAGGAG

TGGAGGTGCCAAAGGCCTATATTTTCAACCAGCACAACGGCATAAATTACGAGACAAGTG

GTCCCCATCTATTTCCCACCCATCAACCCGCCCAACCGGGTGGCCAGGATGGTGGGTTAA

AAACTTATGTGAATGCCCGCCAATCTGGGTGGTGGCATCATCAGCATCAAGGTTAACATA

ATCCTACACACGGTACTTGGGTATATTCTCACACACTCGATTGATGTAAAGAATATTTAA

AGACAACAACATAGGGCAACAGCGGTTAAAAAAACCACATGACGTATGAGCAAGTGGCAA

ATCAATACTTTATCTAGTTATGTTAAGCAAAAAATAACAATAAATCAACTTTTTTTTGAA

GGTTAAGAGTTTACGCAATTTTCTTGAGCGGAAAAAGCGGAAAAAATGTAAGTATGCATA

AATTCTAAATATATCAACAACTGTACATTTTCTGGAGTACTACTACCAGGCAAGAAAGTA

GGTTGATAAAGCTATGCACAAGATCTGTCATATGGATGGGTTTCGCATCTTCTGGATCAT
