# Supplementary material for: Identifying DNA mutations in Sepia, White, Yellow, and Vestigial genes in D. melanogaster - Integrating inquiry-based experiential learning into undergraduate Genetics Laboratory curriculum
Source: PLoS One. 2026 Sep 1;21(9):e0356998. doi: 10.1371/journal.pone.0356998 (PMC13533379; doi:10.1371/journal.pone.0356998)
Supplement: Supplementary file 10 [file pone.0356998.s011.pdf]

Annotated *white* gene sequence

Drosophila melanogaster w FBgn0003996
Location: X: 2,790,599-2,796,466

Exons

Protein coding

Doc Insertion (MUT)

Primer set 1: Forward and Reverse Primer binding sites

Primer set 2: Forward and Reverse Primer binding sites

Primer set 3: Forward and Reverse Primer binding sites

>X02974.2 D. melanogaster white locus database consensus seq (WT)
GGGGAGTGCGGAATGCGCATCATCAACAGTCGCCGTTTGATAAATCATGCATGCAAAGTA
CAGTGCAAACCCCCGAAACGGGACGACAACAGGCGGATTAACAAGAACTCTCTTATTCAC
GATAAGAAGACGCTTCCCACTCAACCTAATCAGTATTCAAAGACAGCCACTCAGCTTATG
AGTACTGCCCAGGTGGGGTACTATCATATTGGTGCAAAGGTGGTCGAATTTTTAATTATT
TTGAACGGAACACATTAGCTAAACATAAACATGTTGTCACTAGTATGTATGTAAGTTAAT
AAAACCCATTTTTGCGGAAAGTAGATAAAAAAAACATTTTTTTTTTTTACTGCACTGGAT
ATCATTGAACTTATCTGATCAGTTTTAAATTTACTTCGATCCAAGGGTATTTGATGTACC
AGGTTCTTTCGATTACCTCTCACTCAAAATGACATTCCACTCAAAGTCAGCGCTGTTTGC
CTCCTTCTCTGTCCACAGAAATATCGCCGTCTCTTTCGCCGCTGCGTCCGCTATCTCTTT
CGCCACCGTTTGTAGCGTTACGTAGCGTCAATGTCCGCCTTCAGTTGCATTTTGTCAGCG
GTTTCGTGACGAAGCTCCAAGCGGTTTACGCCATCAATTAAACACAAAGTGCTGTGCCAA
AACTCCTCTCGCTTCTTATTTTTGTTTGTTTTTTGAGTGATTGGGGTGGTGATTGGTTTT
GGGTGGGTAAGCAGGGGAAAGTGTGAAAAATCCCGGCAATGGGCCAAGAGGATCAGGAGC
TATTAATTCGCGGAGGCAGCAAACACCCATCTGCCGAGCATCTGAACAATGTGAGTAGTA
CATGTGCATACATCTTAAGTTCACTTGATCTATAGGAACTGCGATTGCAACATCAAATTG
TCTGCGGCGTGAGAACTGCGACCCACAAAAATCCCAAACCGCAATTGCACAAACAAATAG
TGACACGAAACAGATTATTCTGGTAGCTGTTCTCGCTATATAAGACAATTTTTGAGATCA
TATCATGATCAAGACATCTAAAGGCATTCATTTTCGACTATATTCTTTTTTACAAAAAAT
ATAACAACCAGATATTTTAAGCTTACCATGAAGTCCTCATTTCTTCCACCTTTCATTCTC
AAATATTTTCTTGCTACACTACACTACACTACACTACATTATACATCGACCCCAAATAGT
TGGATGTAGTAGATCGTAATTAGGGACGCATAACCAGTGGTGGCGTGGGAGGAGTCGGCT
TAAGTTGGCCAACAACATTGCTGGGTGTCTATAACTCTAGGCTTGCCAAGATACTAGATA
CTGTATCCGTATCCATTTCTGGTTGTGTACTCGCATCTTCTACCTGATCTTAATACCTCG
TTGTTTGCACGTCTCGCTCGACGAAAAATGTACAATCTAGTCTTATCTGGGTCATTATTT
GGCTAGACGAATGCTTTGGGCTCAGCATCTGATATCTAGGTATCTTCGTGCGTATCTTGC
TTTAAATTCTTAGCACCTCGGCTTGTATAACAAAATAAATAAGTGAGTACGATTTGCATA
TCTAGCCCCGGGCTCTTTGAAACAATTTTGAAAAGTCTCAAAAAGTTATACAAGGAGATA
AGAACTTTAATTCTTTTGGGAAGTAAGTAACGCAGTAAAGGTAACAAAGTATTGAAAAAT
ATGATATGTATGGAATATTTGAAGCCATCTTTAATTATATGTTCGTTGCATATATGTACA
TATTGGGCCGTTTACGCTCTGATATTTCCTTAATAATATCGAGTGGTCGTCAGCTTTTCC
GCTGAATAATTGCGCCTCCTTCAGTATTTCGTTCTCATCAAAGTGAATGACTTTTGAGTG
CACTCATTAGCCACTCGCACAATCGACAAAACGGGAGGAACAACAAATTGAGTCGCTTCT
TTATTCAGTCATTTCAATTTGTCAAGTTCATCACGATACTCCTTCGCCTCCCTGCACAGC
GCCATCTAGCGCCCACTCCTCGAGTTTCCTTCGTATTTCGTAAGTAATTCAAAAATAGCA
CGCAAAGTAAATAAAAAAAAAAGTAAGCCAAAAAATTAAATAAATATGAAATGTAGCATC
TTTCGCTACTTATTTCAACAAAATACGAAAATAGGGAAAAGGTGGGAGAGTAAACCAGAG
GAGGCAGGGAGATAAGAAAACCCAGTGGGAAAGGGACGGCGAAAACAGAAAAGAAAAGAA
AACTTTTTAATAACATTTTTCGGCCTCATTTGTTGTGAAAACAATTTCGCCATGTCCCTC
TATCTCTTTCCCAGCCATATGGCCATCTCGCTCTGCTCGAGGAGTGCCTGAAAACGGATA
TTACATTTTTTCGCGTCTACCTCTCGTAACGTCACCAATTGTGCTTTTTTTTTGGTAACA
GGCCAAAAACTGGACACTGAGTGGCTAGAAGAATAAGCTGTCAAAGCATCGCAGCAGAAG
ATGAGCAGTGAAGCCTCGCTAAGGAGGAAATGCAACAGTTTTCAGGCTTTGGTCTTTTAG
CTAGATTTTATATGTAGCAATGTAAACGATAAATCCAAATCCCCCAATCGTTTTGAAAAA
TAAATTTAATTTTAAATAGTTCAAAAAATGAAAGTTCTTAGCAGGGCTCACTGTAGCAAG
GACCGCCAGGCGCTGAGTGCAAGGTGGACATTATTTTTTCTTGCTTTTATTGAATGTCAG
CTTTTTATTTGGCCCTCTTCTTCGGATTTTGCCTTTCTCATTTGCTTGCATTTTTTTGCT

TAATTTTTTTTATGATTATTTATACAAATTATTATGAGAGCAGCGGCGGATAAAATGCTT
TCCATATTGTGGCCATCATTTTGTCTTGTTTTTTCCATTTTTTTTTTTTTTTGCCTCGAC
GCCAATTAGTGGATCCCTGTTATTTATGCTTTGCCGCAGGCCGCTGCGATGGCAAGAAAC
CCCACAAATGGAAAACGGAAAATTGTTTATGAAAATGAAAATATGCGCGAACGGAAGTGG
TATGGGGCGAAAAGTTGGGGCGGGGAGGATGGCTTGATTTGACCATACCGCAAACGTCTT
CGGTTACCGCCATTTCGAAAAGGGAAGCCCCACCTGGGCGAAAACTGACAGCTCAGTGGT
TTTGGAAATCACTTTACCCGTAAACAAAATAACGTAAATAAGAATTTGTATTTCGGATTT
ATAAACTTAAACTCAATTGCAGAAGTAGTAGTTTGAACACTATTGCGTTCAACATAATTG
GATCAAGTAAATGGACTGCTTTATACTGCTCATTGCACTTATCTACAAGAGATTGAGTTT
TCCCACCACCCAAAAAAAGCCAAAGTATCCTTAAAGTTTTATATTTAATAAAAACAAAAC
TGGCGAAGCTCAAAGTCCCCAAACGAAAGCCAAAGATGCCAGCGATTTACACTCGCAATT
GTCTTATGAGGTGGCAACATGGTGGAGGAACCTTGCCACTGCCACCACCCATCGTCCCAT
CGCCCCATCGTCCATCGCCCAACGACCTGTGACCCCGTCAACCGTCCACCGCCCACTTTC
AAGGCCATCCACCAAGTGCCAGCCCTAATTTGCACATTTTAAATAGGCCTCGCCATCTCC
TTTTTGGGAAACTTGGAAAACTCGGATCTTGGGCATTTTTATTAAACAGATGGGCTCCTT
CTTTATAAAGCCGAGGGGGGGGGGGGGGGTTAGTGCCCGGAAACAGGGACGGCGGAGCAG
GGATTGGCCAGGCTGGGCTAGATTTATGCACAGACGCCTTCATTTTTATGGACGAACGCC
GTGAAATTGAAAAGTTTTCAGCTTGAAACCTTTTCTAGATGCACAAAAAATAAATAAAAG
TATAAACCTACTTCGTAGGATACTTCGGGGTACTTTTTGTTCGGGGTTAGATGAGCATAA
CGCTTGTAGTTGATATTTGAGATCCCCTATCATTGCAG**GGTGACAGCGGAGCGGCTTCGC**
AGAGCTGCATTAACCAGGGCTTCGGGCAGGCCAAAAACTACGGCACGCTCCGGCCACCCA
GTCCGCCGGAGGACTCCGGTTCAGGGAGCGGCCAACTAGCCGAGAACCTCACCTATGCCT
GGCACAATATGGACATCTTTGGGGCGGTCAATCAGCCGGGCTCCGGATGGCGGCAGCTGG
TCAACCGGACACGCGGACTATTCTGCAACGAGCGACACATACCGGCGCCCAGGAAACATT
TGCTCAAGAACGGTGAGTTTCTATTCGCAGTCGGCTGATCTGTGTGAAATCTTAATAAAG
GGTCCAATTACCAATTTGAAACTCAG**TTTGCGGCGTGGCCTATCCGGGCGAACTTTTGGC**
CGTGATGGGCAGTTCCGGTGCCGGAAAGACGACCCTGCTGAATGCCCTTGCCTTTCGATC
GCCGCAGGGCATCCAAGTATCGCCATCCGGGATGCGACTGCTCAATGGCCAACCTGTGGA
CGCCAAGGAGATGCAGGCCAGGTGCGCCTATGTCCAGCAGGATGACCTCTTTATCGGCTC
CCTAACGGCCAGGGAACACCTGATTTTCCAAGCCATGGTGCGGATGCCACGACATCTGAC
CTATCGGCAGCGAGTGGCCCGCGTGGATCAGGTGATCCAGGAGCTTTCGCTCAGCAAATG
TCAGCACACGATCATCGGTGTGCCCGGCAGGGTGAAAGGTCTGTCCGGCGGAGAAAGGAA
GCGTCTGGCATTCGCCTCCGAGGCTCTAACCGATCCGCCGCTTCTGATCTGCGATGAGCC
CACCTCCGGACTGGACTCCTTTACCGCCCACAGCGTCGTCCAGGTGCTGAAGAAGCTGTC
GCAGAAGGGCAAGACCGTCATCCTGACCATTCATCAGCCGTCTTCCGAGCTGTTTGAGCT
CTTTGACAAGATCCTTCTGATGGCCGAGGGCAGGGTAGCTTTCTTGGGCACTCCCAGCGA
AGCCGTCGACTTCTTTTCCTAGTGAGTTCGATGTGTTTATTAAGGGTATCTAGTATTACA
TAACATCTCAACTCCTATCCAG**CGTGGGTGCCCAGTGTCCTACCAACTACAATCCGGCGG**
ACTTTTACGTACAGGTGTTGGCCGTTGTGCCCGGACGGGAGATCGAGTCCCGTGATCGGA
TCGCCAAGATATGCGACAATTTTGCCATTAGCAAAGTAGCCCGGGATATGGAGCAGTTGT
TGGCCACCAAAAATCTGGAGAAGCCACTGGAGCAGCCGGAGAATGGGTACACCTACAAGG
CCACCTGGTTCATGCAGTTCCGGGCGGTCCTGTGGCGATCCTGGCTGTCGGTGCTCAAGG
AACCACTCCTCGTAAAAGTGCGACTTATTCAGACAACGGTGAGTGGTTCCAGTGGAAACA
AATGATATAACGCTTACAATTCTTGGAAACAAATTCGCTAGATTTTAGATAGAATTGCCT
GATTCCACACCCTTCTTAGTTTTTTTCAATGAGATGTATAGTTTATAGTTTTGCAGAAGA
TAAATAAATTTCATTTAACTCGCGAATATTAATGAGATGCGAGTAACATTTTAATTTGCA
G**ATGGTTGCCATCTTGATTGGCCTCATCTTTTTGGGCCAACAACTCACGCAAGTGGGTGT**
GATGAATATCAACGGAGCCATCTTCCTCTTCCTGACCAACATGACCTTTCAAAACGTCTT
TGCCACGATAAATGTAAGTCATGTTTAGAATACATTTGCATTTCAATAATTTACTAACTT
TCTAATGAATCGATTCGATTTAG**GTGTTCACCTCAGAGCTGCCAGTTTTTATGAGGGAGG**
CCCGAAGTCGACTTTATCGCTGTGACACATACTTTCTGGGCAAAACGATTGCCGAATTGC
CGCTTTTTCTCACAGTGCCACTGGTCTTCACGGCGATTGCCTATCCGATGATCGGACTGC
GGGCCGGAGTGCTGCACTTCTTCAACTGCCTGGCGCTGGTCACTCTGGTGGCCAATGTGT
CAACGTCCTTCGGATATCTAATATCCTGCGCCAGCTCCTCGACCTCGATGGCGCTGTCTG
TGGGTCCGCCGGTTATCATACCATTCCTGCTCTTTGGCGGCTTCTTCTTGAACTCGGGCT
CGGTGCCAGTATACCTCAAATGGTTGTCGTACCTCTCATGGTTCCGTTACGCCAACGAGG
GTCTGCTGATTAACCAATGGGCGGACGTGGAGCCGGGCGAAATTAGCTGCACATCGTCGA
ACACCACGTGCCCCAGTTCGGGCAAGGTCATCCTGGAGACGCTTAACTTCTCCGCCGCCG
ATCTGCCGCTGGACTACGTGGGTCTGGCCATTCTCATCGTGAGCTTCCGGGTGCTCGCAT
ATCTGGCTCTAAGACTTCGGGCCCGACGCAAGGAGTAGCCGACATATATCCGAAATAACT
GCTTGTTTTTTTTTTTTACCATTATTACCATCGTGTTTACTGTTTATTGCCCCCTCAAAAA
GCTAATGTAATTATATTTGTGCCAATAAAAACAAGATATGACCTATAGAATACAAGTATT
TCCCCTTCGAACATCCCCACAAGTAGACTTTGGATTTGTCTTCTAACCAAAAGACTTACA

CACCTGCATACCTTACATCAAAAACTCGTTTATCGCTACATAAAACACCGGGATATATTT
TTTATATACAAACTTTTCAAATCGCGCGCCCTCTTCATAATTCACCTCCACCACACCACG
TTTCGTAGTTGCTCTTTCGCTGTCTCCCACCCGCTCTCCGCAACACATTCACCTTTTGTT
CGACGACCTTGGAGCGACTGTCGTTAGTTCCGCGCGATTCGGTTCGCTCAAATGGTTCCG
AGTGGTTCATTTCGTCTCAATAGAAATTAGTAATAAATATTTGTATGTACAATTTATTTG
CTCCAATATATTTGTATATATTTCCCTCACAGCTATATTTATTCTAATTTAATATTATGA
CTTTTTAAGGTAATTTTTTGTGACCTGTTCGGAGTGATTAGCGTTACAATTTGAACTGAA
AGTGACATCCAGTGTTTGTTCCTTGTGTAGATGCATCTCAAAAAAATGGTGGGCATAATA
GTGTTGTTTATATATATCAAAAATAACAACTATAATAATAAGAATACA

>X02974.2 and X17551 Drosophila melanogaster white locus with Doc Insertion (MUT)
TGCGCATCATCAACAGTCGCCGTTTGATAAATCATGCATGCAAAGTACAGTGCAAACCCCCGAAACGGGACGACAACAGGCGGATT
AACAAGAACTCTCTTATTCACGATAAGAAGACGCTTCCCACTCAACCTAATCAGTATTCAAAGACAGCCACTCAGCTTATGAGTAC
TGCCCAGGTGGGGTACTATCATATTGGTGCAAAGGTGGTCGAATTTTTAATTATTTTGAACGGAACACATTAGCTAAACATAAACA
TGTTGTCACTAGTATGTATGTAAGTTAATAAAACCCTTTTTTGGAGAATGTAGATTTAAAAAAACATATTTTTTTTTTTATTTTTTA
CTGCACTGGACATCATTGAACTTATCTGATCAGTTTTAAATTTACTTCGATCCAAGGGTATTTGAAGTACCAGGTTCTTTCGATTA
CCTCTCACTCAAAATGACATTCCACTCAAAGTCAGCGCTGTTTGCCTCCTTCTCTGTCCACAGAAATATCGCCGTCTCTTTCGCCG
CTGCGTCCGCTATCTCTTTCGCCACCGTTTGTAGCGTTACCTAGCGTCAATGTCCGCCTTgacattcggcattccacagtcttcgg
gtggagacgtgtttctttcaagctacgaatagcaagttctaaaaactacaacagtatagtgaaagttaaacacaaagtgtaaagtg
cagtttgcacaactaacaattattgactatagtaattatttactaaaataaataattattccatattgttctggtaattgttatat
gtggacttagaacaatgaatcaaaacgacatacgttctcagcgacaatgtgaacaagacgagcgccggctctctttacaacgcaac
aatgcatactttttctttcgtctcaccgcaaatcggtgatcgagcaccctcaccttcaactaactcgaaacttttgccctcagcgaa
cgacagaccgcgttcttgctctccctctctgcctgcttcggctcacaagtcgtggagcgaagagaccgcctctcctaccccgctcc
tctcgcagcgccaaacgaccgtcccgggtaactgtaacactgcaataacgagtgcagtgacctcactggcaactgccacaacatca
acttcgtcagcggcccaactaattatcgctgtgccagctgtaaataattcagcagcactgaccgtttgcaacaacaataatgcacg
taaagaagaatcaaaacaaaagcagaagtcgatttcgactgtgcagactggcatggatcgctacatccaaatcaagagaaagctca
gccctcaaaacaataaggcaggtaatcaacccaaaatcaatcgaaccaacaacggcaatgaaaactctgcagtaaataattcaaac
cgatatgctatcttggctgattctgcgaccgaacaacccaacgaaaaaacggtaggggaaccaaaaaagaccaggcctccaccaat
tttcatacgagaacaaagtacaaatgcacttgtaaataaactcgttgctttgattggtgacagcaagttccacattatcccactta
aaaaaggaaatattcatgaaataaaactacagatccaaacagaagcagaccaccgtatagtgactaaatacctaaatgatgctggt
aaaaactactacacataccaattaaaaagttgcaaagggctacaggtagtacttaagggcattgaagcaacagtgacaccagctga
gataattgaggctctgaaggccaaaaactttctgcaaagacagctattaatattttaaacaaagacaaagttccgcagccactat
tcaaaatagaactcgaaccagagctccaggcactaaagaaaaacgaagtgcacccaatatacaatttacagtacttgctacatcgg
aggatcaccgtggaggagccgcacaaacgtatcaatccagttcaatgtactaattgccaagaatacggccacaccaaggcatactg
caccccttaagtccgtatgtgttgtctgtagcgaacctcatactaccgcaaactgccccaaaaacaaggacgataagtctgtgaaga
aatgcagtaactgcggggaaaaacatactgcaaactacagaggctgtgtggtgtacaaagaattgaagagccgcctaaacaaacgt
attgccacagcacatacatacaacaaagtcaatttctactctccgcaaccgatttttcaaccacccctaactgtcccaagcactac
tccaacaatttctttcgctagcgccctaaaatccggactagaagtgcccgccccaccgacaagaactgctcattccgaacatacac
cgacaaacatccaacaaacacaacaaagtggcatcgaagctatgatgctatccctacagcaaagcatgaaagacttcatgacgttc
atgcaaaatactttgcaagagctcatgaaaaaccaaaatatcctgattcaacttcttgtatcttcaaaatccccataatggcttcc
ctacggatatctctgtggaacgcaaatggcgtttcacggcatacacaagagctcacacagttcatttacgaaaaaaacatcgacgt
aatgctactatcagaaacgcacctcacaaataaaaacaattttcatataccaggatacttgttctatggtacaaatcatccagatg
gtaaagctcatggaggcactggaatactcatcagaaatcgcataaaacaccaccacttaaacaattttgacaaaaactacttacaa
tctacgtccatagccttacaactcaacaatggttcaacgactctagccgcagtctactgcccaccgcgctttccaatctctgagga
tcaattcatggaattctttaacacactaggtgacaggttcatcgcagcgggtgactataacgccaagcacacccattggggatctc
gacttgtgtcgccaaagggtaagcaattgtacaatgcgcttacgaagccagaaaacaagctagactatgtatccccgggtaagcct
acatactggccagcagacccaagaaaaatcccagacctgatcgattttgcaattactaaacatgtcccccgcaacatggtcaccgc
cgaagcactagcagatttatcatcagatcactcacctgtttttctaaatatgctaactcgcccccacatcgtcgacccaccgtata
gactcacaaattttagaacaaactggccaaggtatcaaaagtatgtctgttcacacatagaactaacgacggcattatctacaaag
gaggatatagacaagtcaacggaaactcttgaaaacatttagtttcggctgcaaaggcttcaaccccgccagtgacgtatgcaaa
accaaactacatcaaaactaatcgcgaaatcgagcggctggtattagataaacgacgcctacgaagggattggcagtctaatagat
caccaattactaagcacatgcttaagatagccacacgcaggcttaccaatgctctcaaacaagaggaaaaaaacagccaacgttca
tatatcgagcaactctctcccaccagcactaagtaccctctttggagagctcacagaaacctaaagactccaatagcgccaattat
gccactccgaagtccctctggcacctggtttcgaagtgatgaagaaagagccagtgctttcgctgaccatttacaaaatgtattcc
gaccaaatccctctaccaacacatttattctccctcctttaatagcagccaatctagatcctcaagaaccctttgaattccgacca
tgtgaactagcaaaggttatcaaagagcaactgaacccaagaaaatcgcctggctacgacctaataactccaagaatgctcattga
actcccaaagtgtgctattcttcacatctgcctgttgttcaacgcaatcgccaagcttggatacttccctcaaaaatggaaaaagt
cgaccatagtaatgattccaaagccaggaaaagataaaacgcagccatcatcatatagaccgataagcttactaacatgtctttca
aagctgtttgaaaaaatgctactccttcggattagccctcatcttagaataaacaacacacttccaacacatcaatttggctttag
agaaaaacatggaaccatcgaacaggtcaaccgaatcacgtcagaaattcgtactgcttttgaacatcgagaatactgcacagcca

tttttctagacgtcgcgcaggcatttgacagagtgtggctcgatggacttttgtttaaaataatcaagctgttgccccaaaacaca
cataagctactgaagtcatacctatataacagagtgtttgcaataagatgcgatacaagcacttcacgcgattgcgcaatcgaagc
tggagtgccgcaaggcagtgtactgggtccaatcttatacaccctgtatacggcggatttccccatagactacaatctaacaacct
ccacgttcgctgatgataccgcgatactcagtcgctcgaaatgcccaataaaagccacggcactcctatcccgacacttaacatct
gtagaacgatggcttgccgactggagaatttcaataaatgttcaaaaatgcaagcaggttacctttaccttaaacaaacaaacatg
cccaccactggtcttgaataacatatgcattccacaagccgacgaggtaacatatctgggagttcatctggacaggcggctcactt
ggcgcaaacatatagaagccaaatcgaaacatcttaaacttaaagcaaggaacctccactggctcataaatgctcgctctccactt
agtctggagttcaaagctcttctatacaactccgtcttaaaacctatctggacttatggctccgagctgtggggcaacgcatccag
aagtaacatagacattattcagcgagcacagtcaagaattctgagaattatcactggagcgccgtggtaccttcgaaacgaaaaca
tacacagagacctaaaaatcaaattagtaatcgaagtaatagctgagaaaaaaacgaagtataacgaaaagctgaccacccataca
aatcccctcgcaagaaaactaatccgagtatgcagtcaaagccggctgcaccgcaacgacctcccagcccagcaataaacttatta
gggcattaatgaaaaaaaaaaaactatcactaagtgaaagttaattaagttagattaagatttgaacacttattgttagtctcttaa
cacaaagggaagattcaataaataataaaaattaaaaaaaaaaaaaaaaaaaaaaAATGTCCGCCTTCAGTTGCACTTTGTCAGCG
GTTTCGTGACGAAGCTCCAAGCGGTTTACGCCATCAATTAAACACAAAGTGCTGTGCCAAAACTCCTCTCGCTTCTTATTTTTGTT
TGTTTTTTTGAGTGATTGGGGTGGTGATTGGTTTTGGGTGGGTAAGCAGGGGAAAGTGTGAAAAATCCCGGCAATGGCCAAGAGGA
TCAGGAGCTATTAATTCGCGGAGGCAGCAAACACCCATCTGCCGAGCATCTGAACAATGTGAGTAGTACATGTGCATACATCTTAA
GTTCACTTGATCTATAGGAACTGCGATTGCAACATCAAATTGTCTGCGGCGTGAGAACTGCGACCCACAAAAATCCCAAACCGCAA
TCGCACAAACAAATAGTGACACGAAACAGATTATTCTGGTAGCTGTGCTCGCTATATAAGACAATTTTTAAGATCATATCATGATC
AAGACATCTAAAGGCATTCATTTTCGACTACATTCTTTTTTACAAAAAATATAACAACCAGATATTTTAAGCTTACCATGAAGTCC
TCATTTCTTCCACCTTTCATTCTCAAATATTTTTTTGCTACACTACACTACACTACACTACGTTATACATCGACCCCAAATAGTTG
GATGTAGTAGATCGTAATTAGGGACGCATAACCAGTGGGGGCGTGGGAGGAGTCGGCTTAAGTTGGCCAACAACATTGCTGGGTGT
CTATAACTCTAGGCTTGCCAAGATACTAGATACTGTATCCGTATCCATTTCTGGTTGTGTACTCGCATCTTCTACCTGATCTTAAT
ACCTCGTTGTTTGCACGTCTCGCTCGACGAAAAATGTACAATCTAGTCTTATCTGGGTCATTATTTGGCTAGACGAATGCTTTGGG
CTCAGCATCTGATATCTAGGTATCTTCGTGCGTATCTTGCTTTAAATTCTTAGCACCTCGGCTTGTATAACAAAATAAATAAGTGA
GCACGATATGCATATCTAGCCCGGGCTCTTTGAAACAATTTTGAAAAGTCTCAAAAAGTTATA
