# Supplementary material for: Identifying DNA mutations in Sepia, White, Yellow, and Vestigial genes in D. melanogaster - Integrating inquiry-based experiential learning into undergraduate Genetics Laboratory curriculum
Source: PLoS One. 2026 Sep 1;21(9):e0356998. doi: 10.1371/journal.pone.0356998 (PMC13533379; doi:10.1371/journal.pone.0356998)
Supplement: Supplementary file 11 [file pone.0356998.s012.pdf]

Section: ______

Please tell us about your experience in the **Molecular Module** of this course (specifically regarding the **white gene analysis**, which was covered from week 3 - week 12 of PCB3023L).

Your responses are anonymous.

1. What was the most interesting or useful thing (e.g. content knowledge, laboratory, analytical, or communication skill) you learned from this lab course **and why**?

2. What do you think you'll remember 5 years from now?
