# Supplementary material for: Identifying DNA mutations in Sepia, White, Yellow, and Vestigial genes in D. melanogaster - Integrating inquiry-based experiential learning into undergraduate Genetics Laboratory curriculum
Source: PLoS One. 2026 Sep 1;21(9):e0356998. doi: 10.1371/journal.pone.0356998 (PMC13533379; doi:10.1371/journal.pone.0356998)
Supplement: Supplementary file 12 [file pone.0356998.s014.pdf]

The manuscript “Identifying DNA Mutations in Sepia, White, Yellow, and Vestigial Genes in *D. melanogaster* – Integrating Hypothesis-Driven Research into Undergraduate Genetics Laboratory Curriculum” by Rentachintala et al., describes an undergraduate genetics laboratory curriculum that integrates semester-long experimentation into a course (using a CURE model) using the well-established model organism *Drosophila melanogaster*. Overall, this curriculum is thoughtfully designed and will be of interest to instructors seeking to incorporate hands-on research-based learning into their genetics laboratory courses. The authors present a solid rationale for the course, articulate clear learning goals and objectives, and provide sufficient logistical and pedagogical support to justify its adoption by instructors at other institutions.

The manuscript adequately places the curriculum within the broader literature on course-based undergraduate research experiences (CUREs) and inquiry-driven experiential learning. The authors should include a bit more on this, however, as this is really the “meat” of the curriculum since the experimental approaches and questions are all well-established. In fact, I am not entirely comfortable with the work being called “hypothesis-driven” and would suggest the authors change the language to “inquiry-based” since the answers to the questions that the students are addressing in the study are well-known. Future investigations by these authors should be placed on the impacts on students and assessing their gains in the topic (genetics) and/or research (e.g., experimental design, data analysis, independence, confidence, sense of belonging, etc.).

The course structure engages students in the scientific process, from hypothesis generation through experimental design, data analysis, and interpretation. The emphasis on connecting genotype to phenotype through molecular and sequencing-based approaches is particularly well-suited for a genetics laboratory and provides students with valuable, transferable skills. On the contrary, since much is already known about the pathways that control eye and body pigmentation and development and wing development in *Drosophila*, these experiments (and the results from them) are not novel and do not contribute to the scientific understanding of the biological processes targeted in this study. Because of this, the work generated by these students will not be publishable, which is a downside of this curriculum since many other CUREs do novel hypothesis-driven work with their students so that students gain authorship on publications as undergraduates. On a positive note, however, students can base and/or supplement their hypotheses and data analysis with published research in the field, which will guide and enrich their learning. Because of this, I strongly think that the title and repeated uses of the phrase “hypothesis-driven research” are misleading and should be changed to “inquiry-based experiential learning,” as this is more appropriate for how these terms are used in the pedagogical literature. The authors spend quite a bit of time discussing how students develop hypotheses, but I think this misleading.

While the students may be designing their experiments with a hypothesis-centered approach, they are really engaging in validation of known tools using the literature, which seems to fall under the classification of "inquiry based experiential learning" according to the field.

The course is appropriately designed for upper-level undergraduate genetics students with limited prior exposure to core molecular biology concepts and laboratory techniques. At my institution, this work would be suitable for a majors-level introductory biology class, so I think the authors should consider broadening the scope for its use in non-Genetics, but yet still molecular biology-focused, laboratory courses. Prerequisite knowledge and skills are clearly stated, and the inclusion of background resources for instructors are helpful, particularly for faculty who may be new to *Drosophila* or the techniques used. This makes this curriculum especially attractive to new laboratory instructors or faculty, or for those who are interested in breaking away from "cookie cutter" labs. Although this curriculum is still fairly limited in scope and techniques (and feels a bit like the kits and curricula available from miniPCR and BIO-RAD), the approaches could easily apply to study mutant lines that have yet to be molecularly mapped or characterized. This would make the study more authentically hypothesis-driven, too. A nice spin-off or addition to this curriculum could involve students designing CRISPR-based alleles of the genes of interest to address specific follow-up questions that they develop (i.e., knockout to look at compare allele and phenotype severity, complementation analysis, etc.). It would also be great to see gene expression analyses of the alleles with the addition of approaches like qPCR or Western blotting, especially since those tools are feasible and often used in Genetics or Molecular Biology lab courses. On a positive note, the molecular biology experiments are very well-designed and certainly enrich the curriculum by providing students with experience in more "modern" cloning techniques. The discussion of potential modifications and extensions is a strength, demonstrating the flexibility of the curriculum and its applicability across a range of institutional contexts and course formats. I would encourage the authors to add a bit more here, especially for more advanced Genetics, Molecular Biology, or Developmental Biology upper-level undergraduate laboratory courses.

The course schedule (supplemental file S4) and supporting materials are logically organized. Although this isn't for a more comprehensive course design journal, it would be helpful if the authors provided introductory PowerPoints, worksheets, quizzes and other course materials to make the curriculum even more attractive to new adopters. Nonetheless, the provided logistical information regarding materials, organism sourcing, and workflow is especially helpful.

Although integration of assignments to assess student learning was not a major focus of this manuscript, the authors provide timepoints and topics for lab reports throughout the curriculum. It would be ideal if there was also a component that allowed for students to present their work – either to each other or to others outside of their course (in an oral presentation or poster presentation format). While written communication is important in science, so is verbal communication, and it would be ideal if the curriculum could include this.

The "Program evaluation - course feedback design and analysis" section (and related components) of the manuscript are the weakest as they do not investigate student perspectives as is typical of a pedagogical study. The authors are strongly encouraged to collect more data (perhaps at different types of institutions) and to use published instruments to evaluate student gains for this manuscript to really be considered as "research". The Discussion section addresses course effectiveness, drawing on student feedback and outcomes to support the value of the approach, but this is mostly qualitative. Similarly, the authors do not connect the work to the pedagogical literature as much as they should for this work to meet the expectations of the journal for original research. Finally, the authors convey an awareness of inclusive teaching practices and emphasize broad access to authentic research experiences, which aligns well with current best practices in biology education.

<u>Major Revisions</u>

1. The repeated use of the phrase *"hypothesis-driven research"* is misleading in the context of this curriculum and should be reframed. While students design experiments and engage in hypothesis-centered thinking, the biological questions addressed (eye color, pigmentation, wing development) are well-established, and the expected outcomes are already known. This work therefore aligns more closely with inquiry-based experiential learning rather than authentic hypothesis-driven research as defined in the pedagogical literature.
    - The authors should revise the title, abstract, and manuscript text to replace "hypothesis-driven research" with terminology such as inquiry-based, guided inquiry, or experiential research-based learning, as appropriate.
    - The discussion of hypothesis generation should be reframed to emphasize validation, interpretation, and application of known genetic tools, rather than discovery-driven outcomes.

2. The “Program evaluation – course feedback design and analysis” section (lines 620-626) and related components are currently the weakest aspect of the manuscript. As written, it does not meet typical expectations for educational research, as it relies largely on qualitative reflections and limited feedback. Figures S11 and 15 seem like they should be the “meat” of this study; instead, the authors spend much of the manuscript presenting the results of analyzing mutant alleles of genes (as the students would do, by providing sample data), but these data are already known and do not provide additional scientific knowledge to the field. Instead, the study should be reframed to focus on the curriculum and student gains. The main figures (Figure 1-14) and associated text are helpful for an instructor who wants to adopt the curriculum for their course, but these components take up the bulk of the manuscript and detract from the main purpose of the manuscript, which should be the impact of the curriculum on student gains/learning outcomes. Instead, must of these figures should be moved to the supplement and compact those results to perhaps just focus on 1 mutant allele with a point mutation and 1 mutant allele with an insertion in the gene. The repetitive nature of the work presented is distracting and ultimately not helpful to address the learning goals of the course (and the purpose of this manuscript).
    - The authors are strongly encouraged to incorporate published/validated assessment instruments (e.g., measures of student gains in genetics content knowledge, experimental design skills, confidence, independence, or sense of belonging) and use those to assess student gains, perhaps using a standard pre-/post-test model.
    - Ideally, the authors can collect data across multiple course iterations or institution types; this would further strengthen the claims and elevate the manuscript from a course description to a true pedagogical research contribution.
    - The Introduction and Discussion sections should more explicitly connect findings to the existing biology education literature (also mentioned in Minor Revisions).

Minor Revisions

1. The authors should more explicitly acknowledge that the experimental outcomes are not biologically novel, and that student-generated data are unlikely to be publishable. While this does not diminish the educational value of the curriculum, it is an important distinction, especially given the current landscape of CUREs that

emphasize student co-authorship on publications. The manuscript would benefit from a clearer articulation of the educational tradeoffs of this approach and how it compares to more discovery-driven CUREs.

2. Gene names are not consistently formatted properly throughout the manuscript. Standard *Drosophila* nomenclature conventions should be followed (gene names lowercase and italicized and protein names capitalized and non-italicized). This should be corrected throughout the text, figures, and supplementary materials.
3. While the manuscript minimally cites relevant CURE literature, this is the core of the manuscript for publication (otherwise it doesn't really conduct research) and should be expanded. A deeper synthesis of how this curriculum compares to other inquiry-based or research-based genetics labs would strengthen the framing.
4. Although the course is presented as an upper-level Genetics laboratory, the content and techniques would be suitable for majors-level introductory biology laboratory courses at some institutions, such as mine. The authors should consider broadening the stated audience and explicitly discussing this flexibility.
5. The suggested modifications and extensions are a good starting point, but could be expanded further, particularly for advanced courses. Potential additions might include:
    - Molecularly mapping uncharacterized mutant lines (perhaps from forward genetic screens), particularly through PCR and sequence analysis
    - Design of CRISPR/Cas-generated alleles
    - Gene expression analyses (e.g., qPCR or Western blotting) to increase authenticity and better support hypothesis-driven extensions
6. While written communication is assessed through three lab reports in the proposed curriculum, it would be strengthened by incorporating oral or poster-based presentations, either within the course to peers or to a broader audience. This would better align with core scientific communication skills (i.e., AAAS Vision and Change).
7. The curriculum would be even more accessible to new instructors/adopters, especially those not familiar with *Drosophila* genetics or the techniques used herein, if additional materials (e.g., introductory slides, worksheets, quizzes, or example rubrics/assessments) were provided as supplementary files.
