# Supplementary material for: Identifying DNA mutations in Sepia, White, Yellow, and Vestigial genes in D. melanogaster - Integrating inquiry-based experiential learning into undergraduate Genetics Laboratory curriculum
Source: PLoS One. 2026 Sep 1;21(9):e0356998. doi: 10.1371/journal.pone.0356998 (PMC13533379; doi:10.1371/journal.pone.0356998)
Supplement: Supplementary file 13 [file pone.0356998.s016.pdf]

Ref: PONE-D-25-61409

Revisions Overview Statement:

Dear Editors,

We sincerely thank you for your consideration of our manuscript "Identifying DNA mutations in Sepia, White, Yellow, and Vestigial genes in D. melanogaster - Integrating Hypothesis-Driven Research into Undergraduate Genetics Laboratory Curriculum". We have addressed all points to the best of our ability and have provided a point-by-point response to the reviewer comments below.

The majority of the revisions we have made are within the main text, such as **1)** added a new figure (Fig 6, Roles in Implementing Curriculum Updates), **2)** adapted Fig 5 with minor formatting as well as addressing the copy right concerns expressed by the editor (please see additional comments regarding this is below), **3)** adapted Fig 16 to connect student learning outcomes as outlined in the syllabus back to the student reflections collected at the end of the course, **4)** added Supplementary File S1 to include original uncropped and unadjusted images for all gel results (as requested by the editor), **5)** added Supplementary File S5 to make the lab report guidelines and rubrics available (as requested by reviewer 1), **6)** revised and clarified the use of the phrase "hypothesis-driven" throughout the manuscript (as requested by reviewer 1), **7)** included additional discussion regarding CUREs as compared to our curriculum (as requested by reviewer 1), updated the gene and protein name formatting throughout the paper (as requested by reviewer 1), **8)** added discussion regarding the flexibility of the course and broadening the stated audience (as requested by reviewer 1), **9)** added discussion regarding the potential adaptions and companion courses (as requested by reviewer 1), **10)** included details regarding oral and poster presentation opportunities for extracurricular research students, as well as publishing opportunities and limitations pertaining to authorship (as requested by reviewer 1), **11)** provided a transition for the sepia gene results (requested by reviewer 2), **12)** updated the text to specify the replicates of sequencing under methods (as requested by reviewer 2), **13)** referred to Fig 15 within the manuscript text (as requested by the editor), **14)** rearranged the text and adjusted the figure numbers as well as the supplementary file numbers throughout the manuscript and supporting document file names.

Regarding Fig 5, there was a note that it contains copyrighted images. We made this figure ourselves using PowerPoint for the majority of the figure components (using shapes to form figures) and any remaining components using Procreate (to draw the bacterial cell outline). We did use a microscope and DNA shapes/clipart from PowerPoint under the "phenotypic screening" and "DNA extraction" boxes, which we have now removed and replaced with figures that we have created using Procreate (PNG and Procreate files attached). Other than that, since our work is about integrating this into the curriculum, we have adapted a small portion of this figure to include in the lab manuals for our students. This adaptation is also our own work and not copyrighted since it is only printed each semester for our students taking the lab. We have provided a copy of the lab manual to show the adapted figure being used in that context, which has since been updated with major revisions before being included in our manuscript. All of the supporting files are uploaded as "Figure 5 Supporting Documents". Please let us know if there is any further element of this figure that may be in question that we may provide additional supporting documentation.

Reviewer 1:

Summary of Review:

In summary, the curriculum proposed by the authors in the associated manuscript is well designed and pedagogically valuable. However, reframing the work as inquiry-based experiential learning and strengthening the educational research and assessment components are necessary for the manuscript to fully meet the expectations of a pedagogical research study.

We sincerely appreciate the reviewer's comments to improve the quality of the manuscript.

Major Revisions

1. The repeated use of the phrase "hypothesis-driven research" is misleading in the context of this curriculum and should be reframed. While students design experiments and engage in hypothesis-centered thinking, the biological questions addressed (eye color, pigmentation, wing development) are well-established, and the expected outcomes are already known. This work therefore aligns more closely with inquiry-based experiential learning rather than authentic hypothesis-driven research as defined in the pedagogical literature. The authors should revise the title, abstract, and manuscript text to replace "hypothesis-driven research" with terminology such as inquiry-based, guided inquiry, or experiential research-

based learning, as appropriate. The discussion of hypothesis generation should be reframed to emphasize validation, interpretation, and application of known genetic tools, rather than discovery-driven outcomes.

We thank the reviewer for this suggestion. We have updated the manuscript to reflect this change in the context of the curriculum in the title, abstract, and within the manuscript text. We have reframed the hypothesis-driven aspect to more accurately represent the work done by extracurricular research students throughout the manuscript and have created Fig 6 to more clearly show the workflow for curriculum development.

The inquiry-based aspect of the course has been further explained in the methods section, under the subheading "Curriculum Design", on page 16, starting on line 606:

"With respect to the inquiry-based aspect of the course, students are encouraged to review existing literature and form three drafts of their hypothesis statement. They are provided feedback in class and on their lab report rough draft as they continue to generate their working hypothesis based on information they have gathered and the specific details for each lab section. This process offers the instructors to check student understanding and correct misconceptions, while giving students an opportunity to think carefully about the experimental design. We expect the hypothesis to change slightly across course iterations, between sections and student groups, as students collectively work toward uncovering the DNA mutations responsible for the phenotypes under investigation for their population of flies. Instructors facilitate hypothesis formation by ensuring that students are addressing the research question, that the hypothesis is testable, clear, concise, has sufficient rationale, that students have access to fundamental information that is known about the project, and that they know what remains unknown."

2. The "Program evaluation – course feedback design and analysis" section (lines 620- 626) and related components are currently the weakest aspect of the manuscript. As written, it does not meet typical expectations for educational research, as it relies largely on qualitative reflections and limited feedback. Figures S11 and 15 seem like they should be the "meat" of this study; instead, the authors spend much of the manuscript presenting the results of analyzing mutant alleles of genes (as the students would do, by providing sample data), but these data are already known and do not provide additional scientific knowledge to the field. Instead, the study should be reframed to focus on the curriculum and student gains. The main figures (Figure 1- 14) and associated text are helpful for an instructor who wants to adopt the curriculum for their course, but these components take up the bulk of the manuscript and detract from the main purpose of the manuscript, which should be the impact of the curriculum on student gains/learning outcomes. Instead, must of these figures should be moved to the supplement and compact those results to perhaps just focus on 1 mutant allele with a point mutation and 1 mutant allele with an insertion in the gene. The repetitive nature of the work presented is distracting and ultimately not helpful to address the learning goals of the course (and the purpose of this manuscript). The authors are strongly encouraged to incorporate published/validated assessment instruments (e.g., measures of student gains in genetics content knowledge, experimental design skills, confidence, independence, or sense of belonging) and use those to assess student gains, perhaps using a standard pre-/post-test model. Ideally, the authors can collect data across multiple course iterations or institution types; this would further strengthen the claims and elevate the manuscript from a course description to a true pedagogical research contribution. The Introduction and Discussion sections should more explicitly connect findings to the existing biology education literature (also mentioned in Minor Revisions).

We thank the reviewer for this suggestion. We have reframed and included additional details in the methods (under "Curriculum Design" subheading), results (under "Overall Organization for Curriculum Development", "Student Engagement and Outcomes in the Genetics Laboratory Program", and "Extracurricular Student Outcomes" subheadings), and the discussion sections to focus on curriculum development and student gains. We have added a new figure (Fig 6, Roles in Implementing Curriculum Updates). Additionally, we have adapted Fig 16 to connect the student learning outcomes as outlined in the syllabus back to the student reflections collected at the end of the course.

Further, we recognize the value in collecting data across multiple course iterations or institution types. While we are not able to realize this at this moment, this is our long-term aim of this manuscript. Additionally, we have edited the introduction and discussion sections to clarify what is currently known in literature in addition to the novel findings presented in this manuscript.

Minor Revisions

1. The authors should more explicitly acknowledge that the experimental outcomes are not biologically novel, and that student-generated data are unlikely to be publishable. While this does not diminish the educational value of the curriculum, it is an important distinction, especially given the current landscape of CUREs that emphasize student co-authorship on publications. The manuscript would benefit from a clearer articulation of the educational tradeoffs of this approach and how it compares to more discovery-driven CUREs.

We thank the reviewer for this comment. We have updated the manuscript in the introduction and discussion sections to make it more clear what is known and unknown for each gene under investigation by extracurricular research students.

We have clarified that laboratory course student data may not be published, but that their work may be referenced in the acknowledgments section of work published by extracurricular research students. Furthermore, we have clarified that laboratory course students interested in molecular biology research and education have joined as extracurricular research students, which has given them the opportunity to be involved in publishable works. These details are included in the manuscript in the results section, under the "Extracurricular Student Outcomes" on page 7, line 287:

"Graduate and undergraduate research students were involved in the investigation of genes that are part of the curriculum described above, as shown in Fig 6. Students who previously engaged in the laboratory course, who shared their interest in research and education, have joined as extracurricular research students. This enabled them to provide valuable insight from their experiences in the course and contribute to investigation of new genes for integration into the course curriculum. Students engaged in oral and poster presentations at various conference localities (e.g., Undergraduate Research Colloquium and USF Health Research Day hosted by University of South Florida, as well as the Florida Undergraduate Research Conference) to showcase their efforts.

Furthermore, students had an opportunity to engage in manuscript preparation to publish their findings. However, authorship for such a large consortium is associated with some ethical issues, and we are cognizant of the publication challenges that may be associated with this [32]. The students may not merit authorship if they did not participate in the writing, but they may be referenced in acknowledgments where their valuable contributions can be acknowledged."

Additionally, we have elaborated on the benefits of laboratory course student work with respect to reproducibility of the findings and the value they bring in the robustness of the data generated, on page 8, starting on line 321:

"Reproducibility and transparency have been a growing focus for the scientific community, with the US National Institutes of Health (NIH) outlining plans to enhance current processes in the biomedical research field [34]. Underscoring this importance begins at the classroom level, and the structure of our curriculum development design takes into consideration the reproducibility of student findings. The data generated by both the course students and extracurricular research students could be described as a consortium, which is a collaborative pool supporting the reproducibility of these findings. The robustness of the data generated by students is shown in replicates across the many sections and course semester iterations."

2. Gene names are not consistently formatted properly throughout the manuscript. Standard Drosophila nomenclature conventions should be followed (gene names lowercase and italicized and protein names capitalized and non-italicized). This should be corrected throughout the text, figures, and supplementary materials.

We thank the reviewer for this comment. The formatting for gene names and protein names has been corrected throughout the manuscript.

3. While the manuscript minimally cites relevant CURE literature, this is the core of the manuscript for publication (otherwise it doesn't really conduct research) and should be expanded. A deeper synthesis of how this curriculum compares to other inquiry-based or research-based genetics labs would strengthen the framing.

We thank the reviewer for this suggestion. We have created Fig 6 to further make our proposed curriculum updates and roles involved more clear to the audience. Additionally, we have expanded discussion regarding our curriculum and have compared it to relevant CUREs literature, on page 8, starting on line 315:

“A variety of learning models describing course-based undergraduate research experiences (CUREs) have shown to enhance student learning, many of which focus on small class sizes that offer research opportunities for undergraduate students [33]. Our proposed experiential learning curriculum has similar shared student gains, while also being unique from standard CUREs in that it can be applied and adapted to large-scale courses. This has value in being able to reach a large number of students while offering a similar introduction to research and collaborative learning environment.”

4. Although the course is presented as an upper-level Genetics laboratory, the content and techniques would be suitable for majors-level introductory biology laboratory courses at some institutions, such as mine. The authors should consider broadening the stated audience and explicitly discussing this flexibility.

We thank the reviewer for this comment. We have explicitly discussed this flexibility in the discuss section under the subheading “Potential Adaptations and Companion Courses”, provided on page 11, starting on line 457:

“Thus far, we have focused on the curriculum design for the General Genetics Laboratory course at our institution. Primarily, this course curriculum covers DNA level analysis by offering students a specific project that allows students to follow their experiment from phenotypic screening and DNA extraction to DNA sequencing and analysis, ultimately to understand how DNA mutations may impact protein function. This course design can be adapted for lower-level introductory Biology laboratories as well as upper-level Molecular Biology laboratories, depending on the institution and course structure.”

5. The suggested modifications and extensions are a good starting point, but could be expanded further, particularly for advanced courses. Potential additions might include: o Molecularly mapping uncharacterized mutant lines (perhaps from forward genetic screens), particularly through PCR and sequence analysis o Design of CRISPR/Cas-generated alleles o Gene expression analyses (e.g., qPCR or Western blotting) to increase authenticity and better support hypothesis-driven extensions

We thank the reviewer for this suggest and have expanded on this in the discussion regarding potential adaptations and companion courses, provided on page 11, starting on line 463:

“In addition to this DNA level analysis, we suggest potential companion courses that can perform investigations at the RNA and protein level. These courses could be taken concurrently or sequentially, to build upon the techniques and concepts learned in this course. For example, at our institution, three different courses are designed to cover DNA level analysis, RNA level analysis (including RNA extraction, primer design, gene expression analysis), and protein level analysis (including protein isolation, quantification, western blot, and functional analysis). Each of these courses is designed around an overarching experimental design that is followed by students throughout the course of one semester. For advanced courses, potential adaptations can focus on clinical relevance or perhaps molecularly mapping uncharacterized mutant lines and designing CRISPR/Cas9-generated alleles.”

6. While written communication is assessed through three lab reports in the proposed curriculum, it would be strengthened by incorporating oral or poster-based presentations, either within the course to peers or to a broader audience. This would better align with core scientific communication skills (i.e., AAAS Vision and Change).

We thank the reviewer for this suggestion. Graduate and undergraduate extracurricular research students involved in curriculum development, including students who previously engaged in the laboratory course, have participated in oral and poster-based presentation, as well as engaged in manuscript preparation. We have included these details in the manuscript in the results section, under the “Extracurricular Student Outcomes” on page 7, line 288:

“Graduate and undergraduate research students were involved in the investigation of genes that are part of the curriculum described above, as shown in Fig 6. Students who previously engaged in the laboratory course, who shared their interest in research and education, have joined as extracurricular research students. This enabled them to provide valuable insight from their experiences in the course and contribute to investigation of new genes for integration into the course curriculum. Students engaged in oral and poster presentations at various conference localities (e.g., Undergraduate

Research Colloquium and USF Health Research Day hosted by University of South Florida, as well as the Florida Undergraduate Research Conference) to showcase their efforts.

Furthermore, students had an opportunity to engage in manuscript preparation to publish their findings. However, authorship for such a large consortium is associated with some ethical issues, and we are cognizant of the publication challenges that may be associated with this [32]. The students may not merit authorship if they did not participate in the writing, but they may be referenced in acknowledgments where their valuable contributions can be acknowledged."

7. The curriculum would be even more accessible to new instructors/adopters, especially those not familiar with Drosophila genetics or the techniques used herein, if additional materials (e.g., introductory slides, worksheets, quizzes, or example rubrics/assessments) were provided as supplementary files.

We thank the reviewer for this suggestion. We have provided an additional supplementary file (Supplementary File S5, Lab Report Guidelines and Rubric) to provide the general lab report guidelines, along with lab report 2 guidelines and rubric pertaining to the Molecular component of the curriculum.

At this time, we are not able to publicly share the worksheets and quizzes in case they become available for students to access prior to taking the course. However, we are happy to share these materials with new instructors/adopters privately if they are able to reach out to us. We have included the following statement on page 16, line 605:

"Instructors are welcome to reach out to us for any additional course related components and materials."

Reviewer 2:

Summary of Review:

The manuscript is written very well and presents their findings as well as the lay out of an undergraduate upper level Genetic laboratory curriculum in a very organized manner and is overall well put together. It provides an excellent model for other undergraduate courses to incorporate hypothesis driven research and project based activities in their biology laboratory courses. Apart from confirming previously known Drosophila mutants: white gene and yellow mutation, the manuscript also provides novel finding about the molecular nature of the sepia mutant. This work shows that novel findings can arise through undergraduate research based lab curriculum. Students have an opportunity to connect the techniques they learn while working on their projects to real world applications. Further these experiences will lead to even greater impact on their career choices. Students will gain experience in genomic approaches and will be able to apply in future careers such as personalized medicine. I recommend this manuscript and am very enthusiastic for its publication in PlosOne.

We sincerely appreciate the reviewer's comments to improve the quality of the manuscript.

Minor Comments:

#1. The transition from describing Fig7A and &B in results section on pg 6 is abrupt. The first paragraph describes amplifying the sepia gene using PS1 and PS2 from genomic DNA. The next paragraph switches to restriction digestion of plasmids (Fig7B) containing wild type sepia gene and the mutant gene. It is not clear if these plasmids were made in this study from the above amplified wild type and mutant sepia gene. However as you read the rest of the manuscript however their strategy becomes clear.

We thank the reviewer for this observation and agree that the transition is abrupt without further context. This has now been amended in the manuscript on page 4, line 131:

"These resulting PCR products were ligated into TOPO vectors by extracurricular research students to generate plasmids for use in the laboratory course. The resulting recombinant plasmids were subjected to EcoRI restriction digestion."

#2 and #3. pg 24 line 543, should be across replicate sequence data and specify how many replicates of sequencing were used in this same section under methods

We thank the reviewer for their comments, and have updated the text on page 15, line 572:

“...consensus sequences were generated from data obtained by following highest quality chromatogram reads across greater than 50 replicate sequence data (for fall or spring semesters) per fragment of interest.”
