# Supplementary material for: Identifying DNA mutations in Sepia, White, Yellow, and Vestigial genes in D. melanogaster - Integrating inquiry-based experiential learning into undergraduate Genetics Laboratory curriculum
Source: PLoS One. 2026 Sep 1;21(9):e0356998. doi: 10.1371/journal.pone.0356998 (PMC13533379; doi:10.1371/journal.pone.0356998)
Supplement: Peer review history

# Author comment: Author response to Decision Letter 0

**DOI:** 10.1371/journal.pone.0356998.r001

**Transfer Alert**

This paper was transferred from another journal. As a result, its full editorial history (including decision letters, peer reviews and author responses) may not be present.

14 Nov 2025

# Peer review: Decision Letter 0

**DOI:** 10.1371/journal.pone.0356998.r002

**Authors:** Krishna Bhat

17 May 2026

Dear Dr. Rentachintala,

Thank you for submitting your interesting manuscript to PLOS ONE. After careful consideration, we feel that it has strong merit but does not fully meet PLOS ONE’s publication criteria as it currently stands. Therefore, we invite you to submit a revised version of the manuscript that addresses the points raised during the review process.

As you can see the two reviewers like your work, one requests major changes, and the other requests response to critiques. I think it will eb good for authors to revise the work as much as possible, and respond to critiques as best as can. Include changes to the manuscript by adding the responses to critiques in the manuscript so the revisions represent a quality work.

Please submit your revised manuscript by Jul 01 2026 11:59PM. If you will need more time than this to complete your revisions, please reply to this message or contact the journal office at plosone@plos.org . When you're ready to submit your revision, log on to https://www.editorialmanager.com/pone/ and select the 'Submissions Needing Revision' folder to locate your manuscript file.

- A letter that responds to each point raised by the academic editor and reviewer(s). You should upload this letter as a separate file labeled 'Response to Reviewers'.
- A marked-up copy of your manuscript that highlights changes made to the original version. You should upload this as a separate file labeled 'Revised Manuscript with Track Changes'.
- An unmarked version of your revised paper without tracked changes. You should upload this as a separate file labeled 'Manuscript'.

If you would like to make changes to your financial disclosure, please include your updated statement in your cover letter. Guidelines for resubmitting your figure files are available below the reviewer comments at the end of this letter.

If applicable, we recommend that you deposit your laboratory protocols in protocols.io to enhance the reproducibility of your results. Protocols.io assigns your protocol its own identifier (DOI) so that it can be cited independently in the future. For instructions see: https://journals.plos.org/plosone/s/submission-guidelines#loc-laboratory-protocols . Additionally, PLOS ONE offers an option for publishing peer-reviewed Lab Protocol articles, which describe protocols hosted on protocols.io. Read more information on sharing protocols at https://plos.org/protocols?utm_medium=editorial-email&utm_source=authorletters&utm_campaign=protocols .

As the corresponding author, your ORCID iD is verified in the submission system and will appear in the published article. PLOS supports the use of ORCID, and we encourage all coauthors to register for an ORCID iD and use it as well. Please encourage your coauthors to verify their ORCID iD within the submission system before final acceptance, as unverified ORCID iDs will not appear in the published article. *Only*  the individual author can complete the verification step; PLOS staff *cannot*  verify ORCID iDs on behalf of authors.

We look forward to receiving your revised manuscript.

Kind regards,

Krishna Moorthi Bhat, M.D., Ph.D.

Academic Editor

PLOS One

Journal Requirements:

When submitting your revision, we need you to address these additional requirements.

1. Please ensure that your manuscript meets PLOS ONE's style requirements, including those for file naming. The PLOS ONE style templates can be found at

https://journals.plos.org/plosone/s/file?id=wjVg/PLOSOne_formatting_sample_main_body.pdf and

https://journals.plos.org/plosone/s/file?id=ba62/PLOSOne_formatting_sample_title_authors_affiliations.pdf

2. Please ensure that you refer to Figure 15 in your text as, if accepted, production will need this reference to link the reader to the figure.

3. PLOS ONE now requires that authors provide the original uncropped and unadjusted images underlying all blot or gel results reported in a submission’s figures or Supporting Information files. This policy and the journal’s other requirements for blot/gel reporting and figure preparation are described in detail at https://journals.plos.org/plosone/s/figures#loc-blot-and-gel-reporting-requirements and https://journals.plos.org/plosone/s/figures#loc-preparing-figures-from-image-files. When you submit your revised manuscript, please ensure that your figures adhere fully to these guidelines and provide the original underlying images for all blot or gel data reported in your submission. See the following link for instructions on providing the original image data: https://journals.plos.org/plosone/s/figures#loc-original-images-for-blots-and-gels.

In your cover letter, please note whether your blot/gel image data are in Supporting Information or posted at a public data repository, provide the repository URL if relevant, and provide specific details as to which raw blot/gel images, if any, are not available. Email us at plosone@plos.org if you have any questions.

4. We note that Figure 5 in your submission contain copyrighted images. All PLOS content is published under the Creative Commons Attribution License (CC BY 4.0), which means that the manuscript, images, and Supporting Information files will be freely available online, and any third party is permitted to access, download, copy, distribute, and use these materials in any way, even commercially, with proper attribution. For more information, see our copyright guidelines: http://journals.plos.org/plosone/s/licenses-and-copyright.

We require you to either (1) present written permission from the copyright holder to publish these figures specifically under the CC BY 4.0 license, or (2) remove the figures from your submission:

1. You may seek permission from the original copyright holder of Figure 5 to publish the content specifically under the CC BY 4.0 license.

We recommend that you contact the original copyright holder with the Content Permission Form (http://journals.plos.org/plosone/s/file?id=7c09/content-permission-form.pdf) and the following text:

“I request permission for the open-access journal PLOS ONE to publish XXX under the Creative Commons Attribution License (CCAL) CC BY 4.0 (http://creativecommons.org/licenses/by/4.0/). Please be aware that this license allows unrestricted use and distribution, even commercially, by third parties. Please reply and provide explicit written permission to publish XXX under a CC BY license and complete the attached form.”

Please upload the completed Content Permission Form or other proof of granted permissions as an "Other" file with your submission.

In the figure caption of the copyrighted figure, please include the following text: “Reprinted from [ref] under a CC BY license, with permission from [name of publisher], original copyright [original copyright year].”

2. If you are unable to obtain permission from the original copyright holder to publish these figures under the CC BY 4.0 license or if the copyright holder’s requirements are incompatible with the CC BY 4.0 license, please either i) remove the figure or ii) supply a replacement figure that complies with the CC BY 4.0 license. Please check copyright information on all replacement figures and update the figure caption with source information. If applicable, please specify in the figure caption text when a figure is similar but not identical to the original image and is therefore for illustrative purposes only.

5. Please update your submission to use the PLOS LaTeX template. The template and more information on our requirements for LaTeX submissions can be found at http://journals.plos.org/plosone/s/latex.

6. If the reviewer comments include a recommendation to cite specific previously published works, please review and evaluate these publications to determine whether they are relevant and should be cited. There is no requirement to cite these works unless the editor has indicated otherwise.

**Additional Editor Comments:**

Kindly address all the critiques by the reviewers with as many experiments as possible if experiments are involved/necessary, and also write a detailed response to teh critiques, point-by-point when resubmitting the work.

[Note: HTML markup is below. Please do not edit.]

Reviewers' comments:

Reviewer's Responses to Questions

**Comments to the Author**

1. Is the manuscript technically sound, and do the data support the conclusions?

Reviewer #1: Partly

Reviewer #2: Yes

**********

2. Has the statistical analysis been performed appropriately and rigorously?

Reviewer #1: N/A

Reviewer #2: N/A

**********

3. Have the authors made all data underlying the findings in their manuscript fully available?

The PLOS Data policy

Reviewer #1: Yes

Reviewer #2: Yes

**********

4. Is the manuscript presented in an intelligible fashion and written in standard English?

Reviewer #1: Yes

Reviewer #2: Yes

**********

Reviewer #1: Please see the attached file for more information. In summary, the curriculum proposed by the authors in the associated manuscript is well designed and pedagogically valuable. However, reframing the work as inquiry-based experiential learning and strengthening the educational research and assessment components are necessary for the manuscript to fully meet the expectations of a pedagogical research study.

Reviewer #2: The manuscript is written very well and presents their findings as well as the lay out of an undergraduate upper level Genetic laboratory curriculum in a very organized manner and is overall well put together. It provides an excellent model for other undergraduate courses to incorporate hypothesis driven research and project based activities in their biology laboratory courses.

Apart from confirming previously known Drosophila mutants: white gene and yellow mutation, the manuscript also provides novel finding about the molecular nature of the sepia mutant. This work shows that novel findings can arise through undergraduate research based lab curriculum.

Students have an opportunity to connect the techniques they learn while working on their projects to real world applications. Further these experiences will lead to even greater impact on their career choices. Students will gain experience in genomic approaches and will be able to apply in future careers such as personalized medicine.

I recommend this manuscript and am very enthusiastic for its publication in PlosOne.

I only have few minor comment:

1. The transition from describing Fig7A and &B in results section on pg 6 is abrupt. The first paragraph describes amplifying the sepia gene using PS1 and PS2 from genomic DNA.

The next paragraph switches to restriction digestion of plasmids (Fig7B) containing wild type sepia gene and the mutant gene. It is not clear if these plasmids were made in this study from the above amplified wild type and mutant sepia gene.

However as you read the rest of the manuscript however their strategy becomes clear.

2. pg 24 line 543, should be across replicate sequence data

3. specify how many replicates of sequencing were used in this same section under methods

**********

what does this mean? ). If published, this will include your full peer review and any attached files.

If you choose “no”, your identity will remain anonymous but your review may still be made public.

**Do you want your identity to be public for this peer review?**  For information about this choice, including consent withdrawal, please see our Privacy Policy

Reviewer #1: No

Reviewer #2: No

**********

[NOTE: If reviewer comments were submitted as an attachment file, they will be attached to this email and accessible via the submission site. Please log into your account, locate the manuscript record, and check for the action link "View Attachments". If this link does not appear, there are no attachment files.]

To ensure your figures meet our technical requirements, please review our figure guidelines: https://journals.plos.org/plosone/s/figures

You may also use PLOS’s free figure tool, NAAS, to help you prepare publication quality figures: https://journals.plos.org/plosone/s/figures#loc-tools-for-figure-preparation.

NAAS will assess whether your figures meet our technical requirements by comparing each figure against our figure specifications.

Attachment Submitted filename: Rentachintala et al, PLoS ONE.pdf

# Author comment: Author response to Decision Letter 1

**DOI:** 10.1371/journal.pone.0356998.r003

29 Jul 2026

Revisions Overview Statement:

Dear Editors,

We sincerely thank you for your consideration of our manuscript “Identifying DNA Mutations in Sepia, White, Yellow, and Vestigial Genes in D. melanogaster - Integrating Hypothesis-Driven Research into Undergraduate Genetics Laboratory Curriculum”. We have addressed all points to the best of our ability and have provided a point-by-point response to the reviewer comments below.

The majority of the revisions we have made are within the main text, such as 1) added a new figure (Fig 6, Roles in Implementing Curriculum Updates), 2) adapted Fig 5 with minor formatting as well as addressing the copy right concerns expressed by the editor (please see additional comments regarding this is below), 3) adapted Fig 16 to connect student learning outcomes as outlined in the syllabus back to the student reflections collected at the end of the course, 4) added Supplementary File S1 to include original uncropped and unadjusted images for all gel results (as requested by the editor), 5) added Supplementary File S5 to make the lab report guidelines and rubrics available (as requested by reviewer 1), 6) revised and clarified the use of the phrase “hypothesis-driven” throughout the manuscript (as requested by reviewer 1), 7) included additional discussion regarding CUREs as compared to our curriculum (as requested by reviewer 1), updated the gene and protein name formatting throughout the paper (as requested by reviewer 1), 8) added discussion regarding the flexibility of the course and broadening the stated audience (as requested by reviewer 1), 9) added discussion regarding the potential adaptions and companion courses (as requested by reviewer 1), 10) included details regarding oral and poster presentation opportunities for extracurricular research students, as well as publishing opportunities and limitations pertaining to authorship (as requested by reviewer 1), 11) provided a transition for the sepia gene results (requested by reviewer 2), 12) updated the text to specify the replicates of sequencing under methods (as requested by reviewer 2), 13) referred to Fig 15 within the manuscript text (as requested by the editor), 14) rearranged the text and adjusted the figure numbers as well as the supplementary file numbers throughout the manuscript and supporting document file names.

Regarding Fig 5, there was a note that it contains copyrighted images. We made this figure ourselves using PowerPoint for the majority of the figure components (using shapes to form figures) and any remaining components using Procreate (to draw the bacterial cell outline). We did use a microscope and DNA shapes/clipart from PowerPoint under the "phenotypic screening" and "DNA extraction" boxes, which we have now removed and replaced with figures that we have created using Procreate (PNG and Procreate files attached). Other than that, since our work is about integrating this into the curriculum, we have adapted a small portion of this figure to include in the lab manuals for our students. This adaptation is also our own work and not copyrighted since it is only printed each semester for our students taking the lab. We have provided a copy of the lab manual to show the adapted figure being used in that context, which has since been updated with major revisions before being included in our manuscript. All of the supporting files are uploaded as “Figure 5 Supporting Documents”. Please let us know if there is any further element of this figure that may be in question that we may provide additional supporting documentation.

Reviewer 1:

Summary of Review:

In summary, the curriculum proposed by the authors in the associated manuscript is well designed and pedagogically valuable. However, reframing the work as inquiry-based experiential learning and strengthening the educational research and assessment components are necessary for the manuscript to fully meet the expectations of a pedagogical research study.

We sincerely appreciate the reviewer’s comments to improve the quality of the manuscript.

Major Revisions

1. The repeated use of the phrase “hypothesis-driven research” is misleading in the context of this curriculum and should be reframed. While students design experiments and engage in hypothesis-centered thinking, the biological questions addressed (eye color, pigmentation, wing development) are well-established, and the expected outcomes are already known. This work therefore aligns more closely with inquiry-based experiential learning rather than authentic hypothesis-driven research as defined in the pedagogical literature. The authors should revise the title, abstract, and manuscript text to replace “hypothesis-driven research” with terminology such as inquiry-based, guided inquiry, or experiential research-based learning, as appropriate. The discussion of hypothesis generation should be reframed to emphasize validation, interpretation, and application of known genetic tools, rather than discovery-driven outcomes.

We thank the reviewer for this suggestion. We have updated the manuscript to reflect this change in the context of the curriculum in the title, abstract, and within the manuscript text. We have reframed the hypothesis-driven aspect to more accurately represent the work done by extracurricular research students throughout the manuscript and have created Fig 6 to more clearly show the workflow for curriculum development.

The inquiry-based aspect of the course has been further explained in the methods section, under the subheading “Curriculum Design”, on page 16, starting on line 606:

“With respect to the inquiry-based aspect of the course, students are encouraged to review existing literature and form three drafts of their hypothesis statement. They are provided feedback in class and on their lab report rough draft as they continue to generate their working hypothesis based on information they have gathered and the specific details for each lab section. This process offers the instructors to check student understanding and correct misconceptions, while giving students an opportunity to think carefully about the experimental design. We expect the hypothesis to change slightly across course iterations, between sections and student groups, as students collectively work toward uncovering the DNA mutations responsible for the phenotypes under investigation for their population of flies. Instructors facilitate hypothesis formation by ensuring that students are addressing the research question, that the hypothesis is testable, clear, concise, has sufficient rationale, that students have access to fundamental information that is known about the project, and that they know what remains unknown.”

2. The “Program evaluation – course feedback design and analysis” section (lines 620- 626) and related components are currently the weakest aspect of the manuscript. As written, it does not meet typical expectations for educational research, as it relies largely on qualitative reflections and limited feedback. Figures S11 and 15 seem like they should be the “meat” of this study; instead, the authors spend much of the manuscript presenting the results of analyzing mutant alleles of genes (as the students would do, by providing sample data), but these data are already known and do not provide additional scientific knowledge to the field. Instead, the study should be reframed to focus on the curriculum and student gains. The main figures (Figure 1- 14) and associated text are helpful for an instructor who wants to adopt the curriculum for their course, but these components take up the bulk of the manuscript and detract from the main purpose of the manuscript, which should be the impact of the curriculum on student gains/learning outcomes. Instead, must of these figures should be moved to the supplement and compact those results to perhaps just focus on 1 mutant allele with a point mutation and 1 mutant allele with an insertion in the gene. The repetitive nature of the work presented is distracting and ultimately not helpful to address the learning goals of the course (and the purpose of this manuscript). The authors are strongly encouraged to incorporate published/validated assessment instruments (e.g., measures of student gains in genetics content knowledge, experimental design skills, confidence, independence, or sense of belonging) and use those to assess student gains, perhaps using a standard pre-/post-test model. Ideally, the authors can collect data across multiple course iterations or institution types; this would further strengthen the claims and elevate the manuscript from a course description to a true pedagogical research contribution. The Introduction and Discussion sections should more explicitly connect findings to the existing biology education literature (also mentioned in Minor Revisions).

We thank the reviewer for this suggestion. We have reframed and included additional details in the methods (under “Curriculum Design” subheading), results (under “Overall Organization for Curriculum Development”, “Student Engagement and Outcomes in the Genetics Laboratory Program”, and “Extracurricular Student Outcomes” subheadings), and the discussion sections to focus on curriculum development and student gains. We have added a new figure (Fig 6, Roles in Implementing Curriculum Updates). Additionally, we have adapted Fig 16 to connect the student learning outcomes as outlined in the syllabus back to the student reflections collected at the end of the course.

Further, we recognize the value in collecting data across multiple course iterations or institution types. While we are not able to realize this at this moment, this is our long-term aim of this manuscript. Additionally, we have edited the introduction and discussion sections to clarify what is currently known in literature in addition to the novel findings presented in this manuscript.

Minor Revisions

1. The authors should more explicitly acknowledge that the experimental outcomes are not biologically novel, and that student-generated data are unlikely to be publishable. While this does not diminish the educational value of the curriculum, it is an important distinction, especially given the current landscape of CUREs that emphasize student co-authorship on publications. The manuscript would benefit from a clearer articulation of the educational tradeoffs of this approach and how it compares to more discovery-driven CUREs.

We thank the reviewer for this comment. We have updated the manuscript in the introduction and discussion sections to make it more clear what is known and unknown for each gene under investigation by extracurricular research students.

We have clarified that laboratory course student data may not be published, but that their work may be referenced in the acknowledgments section of work published by extracurricular research students. Furthermore, we have clarified that laboratory course students interested in molecular biology research and education have joined as extracurricular research students, which has given them the opportunity to be involved in publishable works. These details are included in the manuscript in the results section, under the “Extracurricular Student Outcomes” on page 7, line 287:

“Graduate and undergraduate research students were involved in the investigation of genes that are part of the curriculum described above, as shown in Fig 6. Students who previously engaged in the laboratory course, who shared their interest in research and education, have joined as extracurricular research students. This enabled them to provide valuable insight from their experiences in the course and contribute to investigation of new genes for integration into the course curriculum. Students engaged in oral and poster presentations at various conference localities (e.g., Undergraduate Research Colloquium and USF Health Research Day hosted by University of South Florida, as well as the Florida Undergraduate Research Conference) to showcase their efforts.

Furthermore, students had an opportunity to engage in manuscript preparation to publish their findings. However, authorship for such a large consortium is associated with some ethical issues, and we are cognizant of the publication challenges that may be associated with this [32]. The students may not merit authorship if they did not participate in the writing, but they may be referenced in acknowledgments where their valuable contributions can be acknowledged.”

Additionally, we have elaborated on the benefits of laboratory course student work with respect to reproducibility of the findings and the value they bring in the robustness of the data generated, on page 8, starting on line 321:

“Reproducibility and transparency have been a growing focus for the scientific community, with the US National Institutes of Health (NIH) outlining plans to enhance current processes in the biomedical research field [34]. Underscoring this importance begins at the classroom level, and the structure of our curriculum development design takes into consideration the reproducibility of student findings. The data generated by both the course students and extracurricular research students could be described as a consortium, which is a collaborative pool supporting the reproducibility of these findings. The robustness of the data generated by students is shown in replicates across the many sections and course semester iterations.”

2. Gene names are not consistently formatted properly throughout the manuscript. Standard Drosophila nomenclature conventions should be followed (gene names lowercase and italicized and protein names capitalized and non-italicized). This should be corrected throughout the text, figures, and supplementary materials.

We thank the reviewer for this comment. The formatting for gene names and protein names has been corrected throughout the manuscript.

3. While the manuscript minimally cites relevant CURE literature, this is the core of the manuscript for publication (otherwise it doesn’t really conduct research) and should be expanded. A deeper synthesis of how this curriculum compares to other inquiry-based or research-based genetics labs would strengthen the framing.

We thank the reviewer for this suggestion. We have created Fig 6 to further make our proposed curriculum updates and roles involved more clear to the audience. Additionally, we have expanded discussion regarding our curriculum and have compared it to relevant CUREs literature, on page 8, starting on line 315:

“A variety of learning models describing course-based undergraduate research experiences (CUREs) have shown to enhance student learning, many of which focus on small class sizes that offer research opportunities for undergraduate students [33]. Our proposed experiential learning curriculum has similar shared student gains, while also being unique from standard CUREs in that it can be applied and adapted to large-scale courses. This has value in being able to reach a large number of students while offering a similar introduction to research and collaborative learning environment.”

4. Although the course is presented as an upper-level Genetics laboratory, the content and techniques would be suitable for majors-level introductory biology laboratory courses at some institutions, such as mine. The authors should consider broadening the stated audience and explicitly discussing this flexibility.

We thank the reviewer for this comment. We have explicitly discussed this flexibility in the discuss section under the subheading “Potential Adaptations and Companion Courses”, provided on page 11, starting on line 457:

“Thus far, we have focused on the curriculum design for the General Genetics Laboratory course at our institution. Primarily, this course curriculum covers DNA level analysis by offering students a specific project that allows students to follow their experiment from phenotypic screening and DNA extraction to DNA sequencing and analysis, ultimately to understand how DNA mutations may impact protein function. This course design can be adapted for lower-level introductory Biology laboratories as well as upper-level Molecular Biology laboratories, depending on the institution and course structure.”

5. The suggest

Attachment Submitted filename: Response Letter to Reviewers and Editor 7_29_2026.pdf

# Editor report: Decision Letter 1

**DOI:** 10.1371/journal.pone.0356998.r004

**Authors:** Krishna Bhat

12 Aug 2026

Identifying DNA mutations in Sepia, White, Yellow, and Vestigial genes in D. melanogaster - Integrating inquiry-based experiential learning into undergraduate Genetics Laboratory curriculum

PONE-D-25-61409R1

Dear Dr. Rentachintala,

We’re pleased to inform you that your manuscript has been judged scientifically suitable for publication and will be formally accepted for publication once it meets all outstanding technical requirements.

Within one week, you’ll receive an e-mail detailing the required amendments. When these have been addressed, you’ll receive a formal acceptance letter and your manuscript will be scheduled for publication.

An invoice will be generated when your article is formally accepted. Please note, if your institution has a publishing partnership with PLOS and your article meets the relevant criteria, all or part of your publication costs will be covered. Please make sure your user information is up-to-date by logging into Editorial Manager at Editorial Manager® and clicking the ‘Update My Information' link at the top of the page. For questions related to billing, please contact billing support .

If your institution or institutions have a press office, please notify them about your upcoming paper to help maximize its impact. If they’ll be preparing press materials, please inform our press team as soon as possible -- no later than 48 hours after receiving the formal acceptance. Your manuscript will remain under strict press embargo until 2 pm Eastern Time on the date of publication. For more information, please contact onepress@plos.org.

Kind regards,

Krishna Moorthi Bhat, M.D., Ph.D.

Academic Editor

PLOS One

Additional Editor Comments (optional):

Reviewers' comments:

# Editor report: Acceptance letter

**DOI:** 10.1371/journal.pone.0356998.r005

**Authors:** Krishna Bhat

PONE-D-25-61409R1

PLOS One

Dear Dr. Rentachintala,

I'm pleased to inform you that your manuscript has been deemed suitable for publication in PLOS One. Congratulations! Your manuscript is now being handed over to our production team.

At this stage, our production department will prepare your paper for publication. This includes ensuring the following:

* All references, tables, and figures are properly cited

* All relevant supporting information is included in the manuscript submission,

* There are no issues that prevent the paper from being properly typeset

You will receive further instructions from the production team, including instructions on how to review your proof when it is ready. Please keep in mind that we are working through a large volume of accepted articles, so please give us a few days to review your paper and let you know the next and final steps.

Lastly, if your institution or institutions have a press office, please let them know about your upcoming paper now to help maximize its impact. If they'll be preparing press materials, please inform our press team within the next 48 hours. Your manuscript will remain under strict press embargo until 2 pm Eastern Time on the date of publication. For more information, please contact onepress@plos.org.

You will receive an invoice from PLOS for your publication fee after your manuscript has reached the completed accept phase. If you receive an email requesting payment before acceptance or for any other service, this may be a phishing scheme. Learn how to identify phishing emails and protect your accounts at https://explore.plos.org/phishing.

If we can help with anything else, please email us at customercare@plos.org.

Thank you for submitting your work to PLOS One and supporting open access.

Kind regards,

PLOS ONE Editorial Office Staff

on behalf of

Professor Krishna Moorthi Bhat

Academic Editor

PLOS One
